# A Molecular Phylogeny of *Plesiorycteropus* Reassigns the Extinct Mammalian Order ‘Bibymalagasia’

**DOI:** 10.1371/journal.pone.0059614

**Published:** 2013-03-26

**Authors:** Michael Buckley

**Affiliations:** Computational and Evolutionary Biology, Faculty of Life Sciences, Manchester Institute of Biotechnology, Manchester, United Kingdom; Zoological Society of London, United Kingdom

## Abstract

Madagascar is well known for its diverse fauna and flora, being home to many species not found anywhere else in the world. However, its biodiversity in the recent past included a range of extinct enigmatic fauna, such as elephant birds, giant lemurs and dwarfed hippopotami. The ‘Malagasy aardvark’ (*Plesiorycteropus*) has remained one of Madagascar’s least well-understood extinct species since its discovery in the 19^th^ century. Initially considered a close relative of the aardvark (*Orycteropus*) within the order Tubulidentata, more recent morphological analyses challenged this placement on the grounds that the identifiably derived traits supporting this allocation were adaptations to digging rather than shared ancestry. Because the skeletal evidence showed many morphological traits diagnostic of different eutherian mammal orders, they could not be used to resolve its closest relatives. As a result, the genus was tentatively assigned its own taxonomic order ‘Bibymalagasia’, yet how this order relates to other eutherian mammal orders remains unclear despite numerous morphological investigations. This research presents the first known molecular sequence data for *Plesiorycteropus*, obtained from the bone protein collagen (I), which places the ‘Malagasy aardvark’ as more closely related to tenrecs than aardvarks. More specifically, *Plesiorycteropus* was recovered within the order Tenrecoidea (golden moles and tenrecs) within Afrotheria, suggesting that the taxonomic order ‘Bibymalagasia’ is obsolete. This research highlights the potential for collagen sequencing in investigating the phylogeny of extinct species as a viable alternative to ancient DNA (aDNA) sequencing, particularly in cases where aDNA cannot be recovered.

## Introduction

The island of Madagascar had a wide range of enigmatic Quaternary fauna that are now extinct, including ‘giants’ (giant lemurs, giant fossa, elephant birds) and ‘dwarfs’ (dwarf and pygmy hippopotami). The variations in size are considered an evolutionary response to insularisation [1], but such changes do not usually obscure their ancestry because many morphological characteristics remain recognisable. However, the ‘Malagasy aardvark’ (*Plesiorycteropus*) is one of the few recently extinct Madagascan mammals that remains poorly understood and its phylogenetic placement within eutherian mammals unclear [2]–[8] despite being discovered over one hundred years ago [9].

The first known collected specimen of this unique digging creature was found at Belo in western Madagascar but remained poorly described [9] until French palaeontologist Charles Lamberton subsequently excavated sites in central and southern Madagascar in the early 20^th^ century. Lamberton [2] recognized that *Plesiorycteropus* displayed the greatest number of resemblances to aardvarks, but subsequently described a series of features strikingly different from tubulidentates, noting that these other features were more akin to those in pangolins, others to armadillos and anteaters and ultimately proposed several hypotheses. These included that it is 1) a ‘palaeontological chimera’ composed of characteristics to distantly related mammals; 2) it is representative of a single type of mammal showing convergently evolved characteristics of distantly related mammals with uncertain affinity; or 3) it is a member of a deep mammalian lineage that, isolated by insularisation, retained ancestral traits that have since been lost in derivative lineages facing more intense selection pressures. Several further studies accepted *Plesiorycteropus* as a tubulidentate, but also remained uncertain as to its phylogenetic placement [3]–[6]. In the 1970s, Patterson [7]–[8] concluded that the hypodigm of *Plesiorycteropus* was indeed only from one type of mammal, and was confident in its systematic position within Tubulidentata considering comparisons with xenarthrans as phylogenetically distracting. Throughout most of the early 20^th^ century, it was regarded as an ‘edentate’ (the former grouping that included Tubulidentata with Pholidota, Cingulata and Pilosa) or more specifically as a tubulidentate and therefore most closely related to the extant aardvark (*Orycteropus*; hence its chosen genus name).

However, towards the end of the 20^th^ century, this association was challenged on the grounds that many of the identifiably derived traits of the skeleton of *Plesiorycteropus*, particularly those associated with scratch-digging, are shared not only by aardvarks but a number of other mammalian groups, including the armadillos (Dasypodidae), pangolins (Manidae), anteaters (Myrmecophagidae), and many others [10]. Of the few derived traits of *Plesiorycteropus* that had no obvious connection with digging, the ones that could be adequately documented were also found not to be unique to aardvarks but instead sharing traits with various groups from ‘Condylarthra’, a paraphyletic grouping within the ungulates [10]. However, no clear relationships between *Plesiorycteropus* and any other group within the ungulates could be confidently identified. Rather than making *Plesiorycteropus* the only recent mammal without a recognised order, a new order was created for it termed ‘Bibymalagasia’ [10]. Ordinal-level placements for recently extinct eutherian mammals is not usually difficult, but the variety of morphological characteristics for *Plesiorycteropus* made it difficult to ascertain which of the apparently derived features document its true affinity [10].

Towards the beginning of the 21^st^ century were the first phylogenies to include *Plesiorycteropus* that sampled widely across mammals [6], [11]–[12]. Even these more recent analyses, some of which used combined morphological and molecular data for extant species but only morphological data for extinct species, could not confidently reconstruct the phylogeny of *Plesiorycteropus* [11]–[12], more often than not placing it next to *Orycteropus* albeit with little support. Although more recent molecular studies on extant species has changed our understanding of phylogenetic relationships among eutherian mammals, such as the pseudoungulates (tubulidentates and the paenungulates Hyracoidea, Sirenia and Proboscidea) [13]–[15], the confident placement of *Plesiorycteropus* has remained elusive.

With novel methods in extracting molecular sequence data from fossils, particularly the sequencing of collagen using soft-ionization mass spectrometry [16]–[17], the various hypotheses for *Plesiorycteropus’* phylogenetic placement can be re-investigated. Given the recent revisions in systematics using molecular sequence data [13]–[14], Lamberton’s ‘edentate’ hypothesis [2] could be considered to place *Plesiorycteropus* as more closely related to any of the former ‘Edentata’ group including the aardvarks, armadillos, anteaters or pangolins; Patterson’s tubulidentate hypothesis [8] would specifically have *Plesiorycteropus* and *Orycteropus* forming a monophyletic group with *Orycteropus*; whereas MacPhee’s ‘Bibymalagasia’ hypothesis [10] would have *Plesiorycteropus* placed separate from all known orders, and not most closely related to Tubulidentata. To resolve the molecular phylogeny of *Plesiorycteropus*, sequence data was obtained for bone type 1 collagen from the fossil specimen and compared with all previously hypothesized closest relatives.

In brief, mass spectrometry is an analytical technique in which molecules are converted to gaseous ions that are subsequently separated in a mass spectrometer and detected. ‘Soft-ionization’ techniques are where the evaporation and ionisation of the molecules into the gaseous phase are carried out without extensive fragmentation, and thus peptides can be analysed (sequenced) without breaking apart into constituent amino acids. The two most commonly used soft-ionization techniques are Matrix Assisted Laser Desorption Ionization (MALDI) and Electrospray Ionization (ESI) [18]–[19], which are linked to various types of mass analyser (e.g., Time of Flight (ToF)) and an ion detector. In a typical ‘shotgun proteomics’ experiment, mixtures of proteins extracted from a sample are collectively digested into fragments (peptides) using a protease (commonly the enzyme trypsin). The peptides are then ionized and their mass-to-charge ratio (*m/z*) determined (in MALDI peptides are most often singly charged, whereas in ESI they more commonly exist in multiple charge states) producing ‘MS spectra’. These peptides can then be further fragmented into smaller peptide fragments and these fragment ions are further analysed by the mass analyzer and recorded as ‘MS/MS spectra’ (also known as ‘MS2 spectra’ or ‘tandem MS spectra’). These MS/MS spectra are then either sequenced manually, or compared with the expected fragmentation patterns of known amino acid sequences within a given protein sequence database (e.g., UniProt) using a specialist search engine (e.g., Mascot), the ‘ion scores’ being indicative of the relative quality of the sequence match.

‘Shotgun proteomics’ techniques are ideal for the analysis of ancient samples because typical protein isolation techniques, such as gel electrophoresis, are often rendered ineffective due to protein degradation. This is because collagen, making up approximately 90% of the total protein in bone, progressively degrades in a way that produces a vast range of collagen fragments that are unpredictable in size [20], and so smear through a polyacrylamide gel; this smearing effect is commonly noted in collagen extracted from archaeological bone [21]–[22]. Their unpredictable range of biochemical properties also negates suitable isolation by many other separation techniques. Sequencing by soft-ionization mass spectrometry now allows us the ability to infer evolutionary relationships from long extinct organisms much deeper into the past than previously thought possible and although the claims of protein sequence retrieval from dinosaur fossils [23] remain controversial [24]–[26], their survival in Early Pleistocene remains (∼1.5 Ma) from temperate climes [27]–[28] at much greater lengths of time than aDNA [29] is widely accepted.

**Figure 1:**
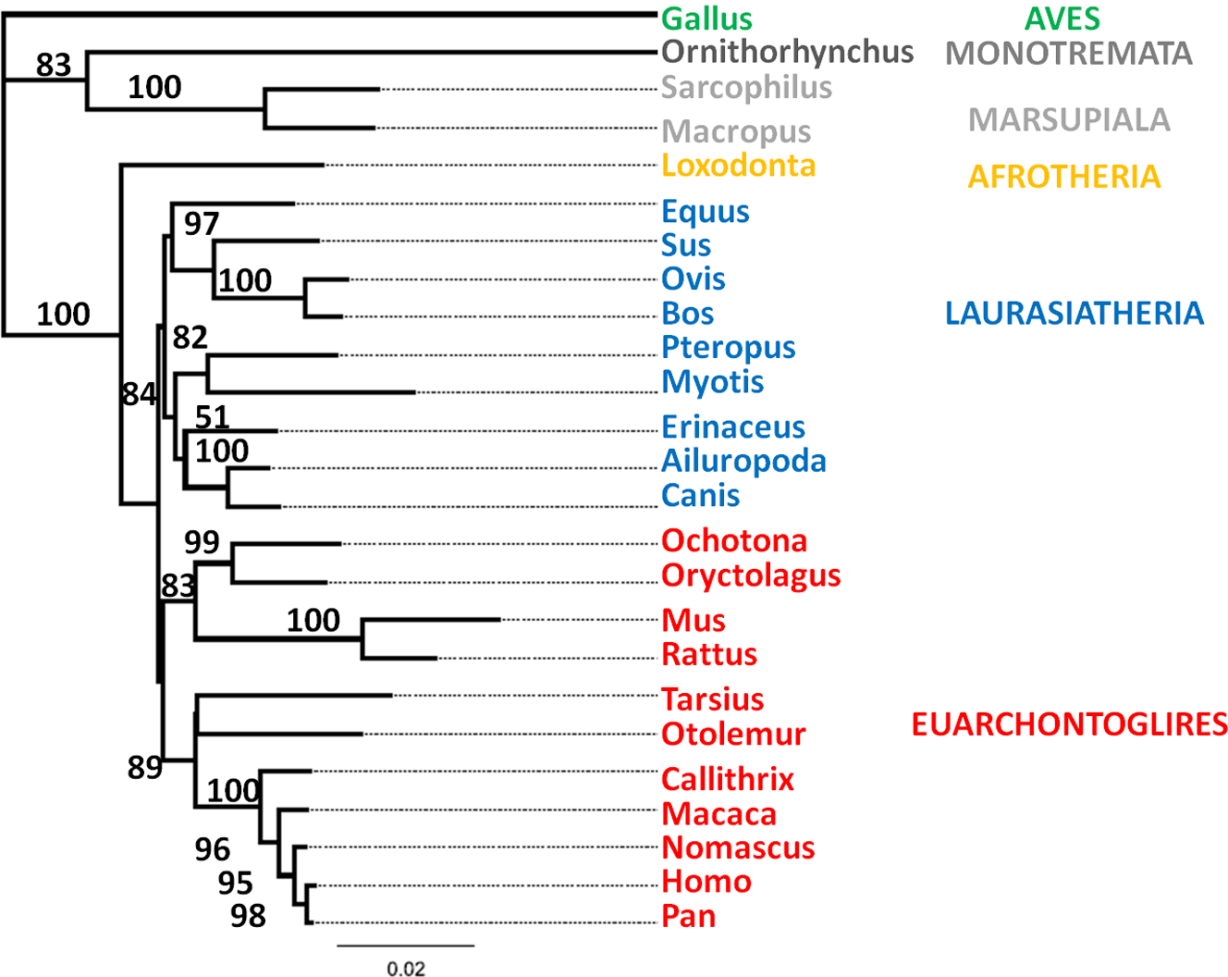
Phylogenetic analysis of 25 concatenated collagen α1 (I) and α2 (I) sequences taken from the Ensembl Genome Browser. Maximum Likelihood analyses produce a phylogenetic signal in good agreement with previously published genomic data (e.g. [13]–[14]). Branch lengths are reported as the mean number of changes per site, bootstrap support is labelled and chicken (*Gallus gallus*) used as the outgroup.

Several proteins, such as albumin and osteocalcin, have been investigated in fossil material as potential alternatives for obtaining phylogenetic information [30]–[31], but collagen (I) is by far the most abundant protein in bone and, because of its highly-insoluble and stable structure, is the most likely protein to be found in fossilised tissues. However, it initially received much less attention than other proteins due to its highly repetitive Gly-Xaa-Yaa amino acid sequence (where Xaa and Yaa can be any amino acid but most frequently Proline and Hydroxyproline respectively). It is a fibrous protein of which the basic unit is made up of two α1(I) chains and a genetically distinct α2(I) chain, which contain an uninterrupted triple helical domain (∼1000 amino acid residues per chain) flanked by short non-helical peptides. Although type I collagen has a highly repetitive amino acid sequence to maintain its triple helical structure, recent studies have shown that this motif is only closely adhered to in one of its two genetically distinct alpha chains (the α1 (I) chain), the other (α2(I)) being much more variable and can offer sequence specificity to at least the genus level [27], [32] and can produce a good phylogenetic signal (Fig. 1). Because collagen is also known to survive greater periods of time than ancient DNA (aDNA) [29] to the extent that it has been reported in Mesozoic dinosaur fossils [17], [23], it is an ideal target for molecular phylogenies of extinct taxa. In this study we use collagen sequences as a viable alternative to aDNA sequencing, which suffers greater contamination issues and requirements of more specialist laboratory arrangements, to place the fossil species *Plesiorycteropus* as a pioneering example of the application of proteomics techniques to palaeobiology.

## Materials and Methods

### Samples

Protein sequencing analyses were carried out on sub-fossil specimens of *Plesiorycteropus* as well as modern samples of postulated close relatives. The sub-fossil *Plesiorycteropus madagascariensis* sample was drilled from femur specimen Oxford University Museum (OUM) 14395, which was collected by Paul Methuen from Antolambiby, Madagascar and presented to the Oxford University Museum of Natural History in 1911. A modern bone specimen aardvark (*Orycteropus afer*) was also obtained from OUM. Additional modern bone specimens obtained from University Museum of Zoology Cambridge (UMZC) included Sunda pangolin (*Manis javanica*) (UMZC E.1308), tamandua (*Tamandua tetradactyla*) (UMZC E.583), giant anteater (*Myrmecophaga tridactyla*) (UMZC E.562), hairy armadillo (*Chaeotophractus villosus*) (UMZC E.1062), golden mole (*Ambylosomus hottentotus*) (NFC-2), greater hedgehog tenrec (*Setifer setosus*) (UMZC 2011.2.2) and rock hyrax (*Procavia capensis*) (UMZC_H.4891.C). A skin cut from modern elephant shrew (*Petrodromus tetradactylus*) (A.533) was also obtained from Manchester Museum. LC-MS data for American mastodon (*Mammut americanum*) was taken from a previous publication [16].

### Protein Extraction and MS Analysis

Soluble proteins were extracted from modern and fossil samples by agitation in 0.6 M hydrochloric acid (HCl) for 4 h and exchanged into 50 mM ammonium bicarbonate using ultrafiltration (30 kDa molecular weight cut-off (MWCO)). The proteins were then digested by trypsin (37°C for 18 h), acidified to 0.1% trifluoroacetic acid (TFA) and further purified and concentrated by C18 ziptip following [16]. Peptide Mass Fingerprints (PMFs) were then obtained by MALDI-ToF-MS and MS/MS spectra acquired for selected peptides. MS/MS spectra were obtained with approximately 500 ion counts for the precursor ion followed by 3000–5000 ion counts for the fragment ions and the peptide sequences interpreted manually. These were used to help create an initial local database that included sequences the species of interest where unknown sequence replaced with the consensus sequence. Digested samples were analysed by LC-MS/MS (Waters nanoAcquity Ultra Performance Liquid Chromatography (UPLC) instrument coupled to a Thermo Scientific Linear Trap Quadrupole (LTQ) Velos Dual Pressure mass spectrometer) on which the peptides were concentrated on a pre-column (20 mm×180 µm) then separated on a 1.7 µM Waters nanoAcquity BEH (Ethylene Bridged Hybrid) C18 analytical column (75 mm×250 µm i.d.), using a gradient from 99% buffer A (0.1% formic acid (FA) in H_2_O)/1% buffer B (0.1% FA in acetonitrile (ACN)) to 25% B in 45 min at 200 nL min^−1^. Peptides were selected for fragmentation automatically by data dependent analysis. Proteomics data files were searched using Mascot against a local database that incorporates UniProt with sequences identified from MALDI-ToF/ToF-MS/MS using two missed cleavages, error tolerances of 0.5 Da (MS and MS/MS) using ‘Error tolerant’ searches with the two (maximum allowed) variable modifications, oxidation of Proline (P) and Lysine (K) that search the Unimods database for further modifications that could occur as a result of amino acid substitution or amino acid modification during diagenesis. Following revision of the sequences from these results, the searches were carried out as normal searches but with pyro-glutamic acid, oxidation (P, K and Methionine (M)) and deamidation of asparagine (N) and glutamine (Q) modifications. LC-MS collagen peptide sequence matches were selected with a peptide ion score >40 and are listed with their respective ion scores in the Supplementary Information (Tables S1–S11).

### Sequence Analysis

The collagen α1(I) and α2(I) sequences were concatenated and aligned with orthologues from 11 LC-MS datasets as well as 26 other species [16], [24]. In sites where isobaric residues (e.g., Isoleucine (I) and Leucine (L)) were present, the most common was used throughout for purposes of phylogenetic inference (listed in Text S1). Phylogenetic trees were created using PhyML v2.0.7 [33] (10,000 bootstrap generations, invariable sites fixed, with four substitution categories) and MrBayes [34] with 1,000,000 bootstrap generations in four chains, first 20% of trees discarded, sampling frequency 1000, heated chain temperature 0.2, with 4 gamma rate variation categories in Geneious v5.5.6 with amino acid models set to Dayhoff. Neighbor-Net analysis was also carried out using the software SplitsTree 4 [35]–[36].

## Results and Discussion

**Figure 2:**
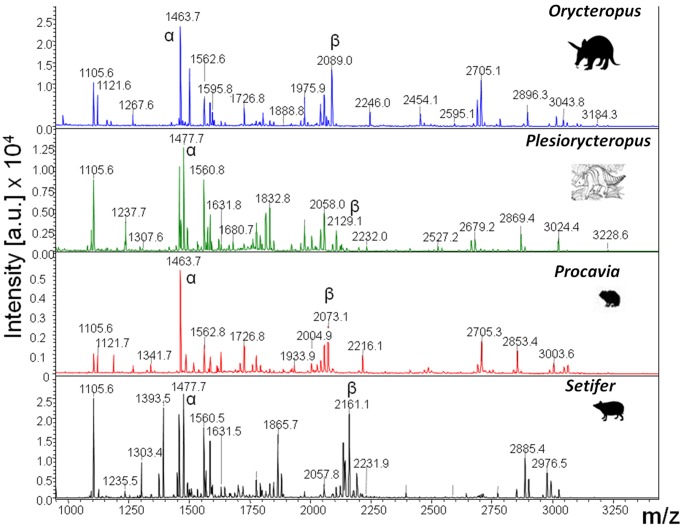
MALDI-ToF-MS spectra of collagen digests from the sub-fossil ‘malagasy aardvark’ *Plesiorycteropus* and modern specimens. The peptide mass fingerprints (PMFs) show that the quality of the collagen extracted from the fossil is as good as from modern specimens (from top to bottom; aardvark (*Orycteropus afer*), *Plesiorycteropus sp.,* rock hyrax (*Procavia capensis*) and greater hedgehog tenrec (*Setifer setosus*)). Peaks marked α and β represent some of the most variable homologous peptides manually sequenced (Fig. 3 & Fig. S2). A greater similarity between peptide *m/z* values can be seen between *Plesiorycteropus* and tenrec than any other species sampled.

**Figure 3:**
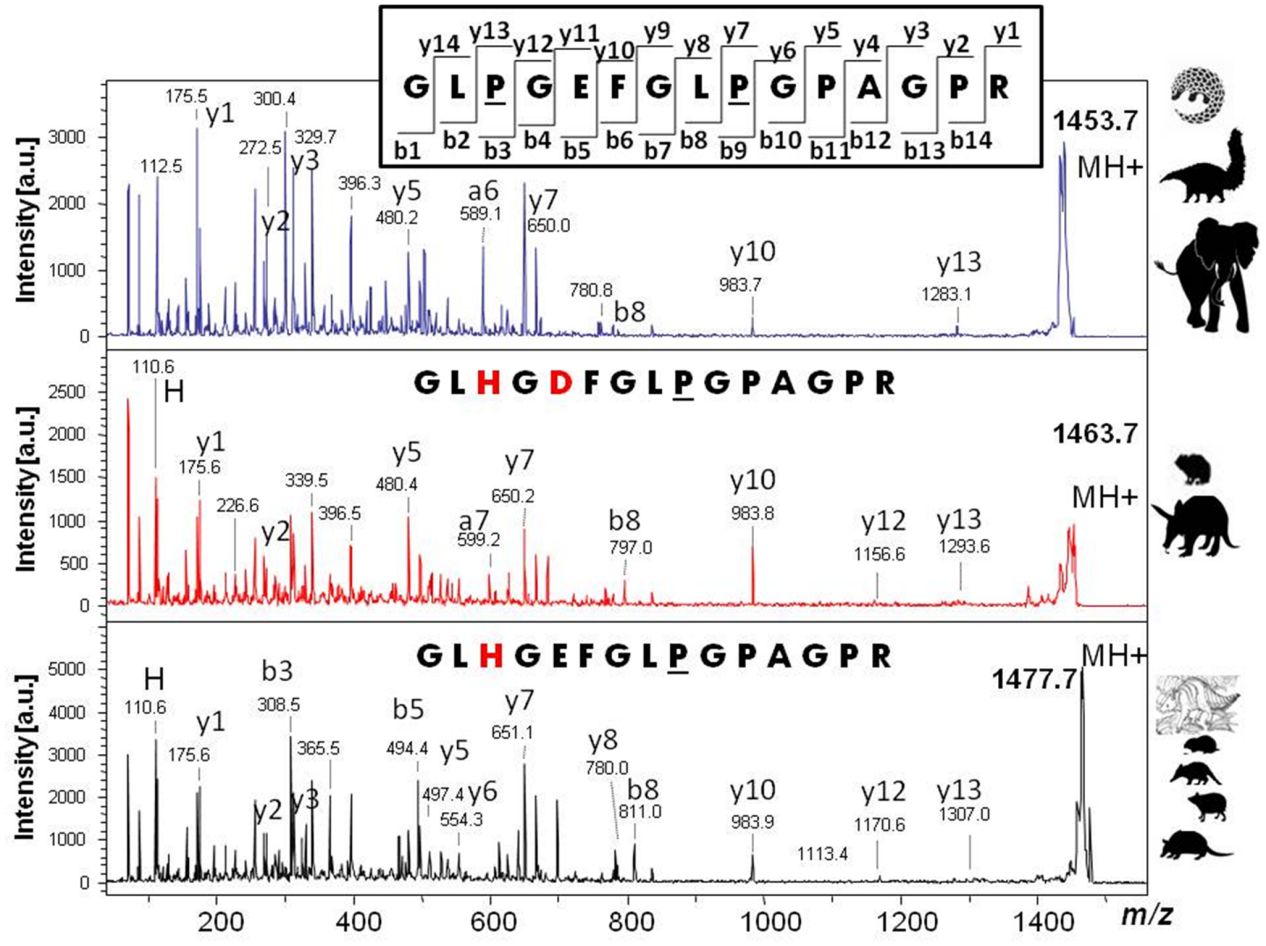
Example tandem MS spectra of three homologous peptides. The spectra are from peptide COL1A2_495–509_ (peak ‘α’ in Fig. 2) with precursor (MH+) ions at (from top to bottom) *m/z* 1453 (elephants, pangolin anteater and tamandua), 1463 (hyrax and aardvark) and 1477 (armadillos and all afrosoricids including the extinct *Plesiorycteropus*). Labeling of fragment ions (inset) follows [37], underline represents hydroxylation site, red text highlights amino acids that differ from the inset (elephant, anteater & pangolin) sequence. Isobaric amino acids (I/L) were estimated based on conserved DNA sequence data where possible; for sites that have more than one isobaric amino acid present, the most abundant was used (see Supplementary Information).

Digests of proteins extracted from a range of candidate phylogenetic neighbours were analysed by MALDI-ToF-MS producing collagen-dominated PMFs (Fig. 2 & Fig. S1). Prior to in depth LC-MS analyses, the *Plesiorycteropus* PMF was compared with those of other mammalian groups in order to identify some of the dominant peptides present that differed between the taxa, particularly the highly variable ‘species biomarker’ peptides described previously [27], [32]. A greater similarity between *Plesiorycteropus* and tenrecs, golden moles and elephant shrews, and between aardvark and hyrax could be observed in these PMFs. However, to get robust statistical support, LC-MS sequencing and subsequent phylogenetic analysis was carried out. Selected peptides with sufficiently high peak intensities in the MALDI-ToF PMFs were analysed by MS/MS for sequence analysis (e.g., Fig. 3 & Fig. S2) and these preliminary sequences were compiled into a local database and searched against using the more comprehensive LC-MS data obtained for each.

**Figure 4:**
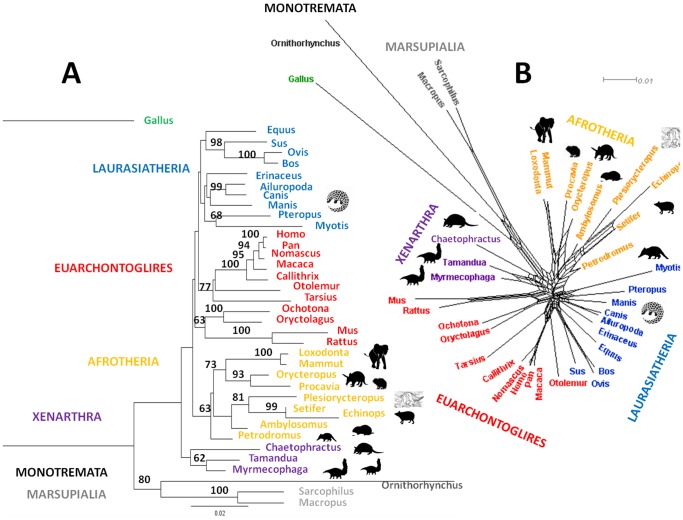
Phylogenetic analyses of *Plesiorycteropus* collagen (I) sequences obtained by LC-MS in comparison to previously postulated closest relatives. The results are from A) Maximum Likelihood (rooted to *Gallus*; only bootstrap support >50 is shown) and B) Neighbor-Net analysis in SplitzTree4. *Plesiorycteropus* consistently forms a monophyletic group with the tenrecs within the order Tenrecoidea, forming a sister group to the aardvark (tubulidentates), rock hyrax, and the proboscideans. Silhouette denotes sequences obtained from LC-MS data; scale shows average amino acid substitutions per site.

Confident peptide sequence matches (ion score >40) from the LC-MS analyses were manually aligned and analysed by Maximum Likelihood (ML; Fig. 4A) and NeighbourNet analysis (Fig. 4B). Even in cases where a high level of peptide match confidence is assumed, the tree topology with regards the species analysed in this study remained consistent where *Plesiorycteropus* always groups with tenrecs within the order Tenrecoidea (Fig. 4 & Fig. S3).

One major limitation of protein (peptide) sequencing by soft-ionization mass spectrometry relies on the matching of peptide fragments ions observed in tandem mass spectra with those predicted from a given database. Thus for fragment ion mass spectra deriving from extinct taxa that do not have closely-related representative species, levels of confidence need to be placed on inference from such data. In the case of collagen (I) analyses, the typical sequence coverage for unconstrained searches is approximately 70–80%, which reduces to ∼50% when appropriate (∼40) ion scores are used. Thus with increasing confidence thresholds in peptide matching using conventional approaches (e.g., Mascot algorithms) there is an observable increase in the amount of missing sequence information.

The sequence analyses (Fig. 4) consistently place *Plesiorycteropus* within Afrotheria, and more specifically as being more closely related to tenrecs than any other previously postulated species (Bootstrap score = 81). Because it consistently forms a monophyletic grouping with tenrecs within a clade that includes the golden moles, these results not only support the order Tenrecoidea (golden moles and tenrecs) but include *Plesiorycteropus* as a new member of this order. The close relationship consistently observed between hyracoids and tubulidentates has long been considered on morphological grounds [38] and supported by early molecular systematic studies using proteins [39]–[40] but differs from more recent nucleotide data showing closer affinities between hyracoids and proboscideans [13]. With the exclusion of Tubulidentata, it is the Afroinsectivora (Tenrecoidea+Macroscelidea) that is consistently monophyletic, not Afroinsectiphilia (Tenrecoidea+Tubulidentata). The placement of the pangolins (Pholidota) as closely related to Carnivora (Fig. 4), as well as the grouping of the xenarthrans (Fig. 4) is consistent with other molecular sequence data [13]–[14], further supporting the use of proteomics-derived collagen sequence data. Interestingly, both the Maximum Likelihood and Neighbour-Net analyses recovered the xenarthrans as more basal than afrotherians. The only discrepancy between these phylogenetic methods lies in the higher-level groupings (e.g., super-order), in which the ML methods do not recover the euarchontoglires as forming a monophyletic clade (Euarchonta and Laurasiatheria forming a sister group to Glires), which is recovered with the NeighborNet analyses.

However, the results clearly show that *Plesiorycteropus* is more closely related to tenrecoids than to tubulidentates, and that because its collagen sequence confidently places it within the order Tenrecoidea, the molecular phylogeny obtained does not support the creation of the taxonomic order ‘Bibymalagasia’ that was proposed in the mid 1990s [10]. Although *Plesiorycteropus* retained some morphological characteristics from its earlier common ancestor with aardvarks, characters such as its relatively small eyes and adaptations to climbing [2], [10] are consistent with other members of Tenrecoidea. Given that its resolved affinity is to Tenrecoidea, particularly close to the tenrecs, which can occupy a diverse range of environments including terrestrial, fossorial, arboreal and aquatic ones [41], it is perhaps not surprising to find this much larger extinct form that acquired a wide range of morphological attributes that has made it so difficult to place for so long.

### Conclusions

The inconclusive results from multiple investigations over the past one hundred years [2]–[8], [10]–[12] illustrate how difficult it is to place isolated taxa displaying many morphological novelties but few obvious synapomorphies. This problem can occur at any hierarchical level, but it is most prevalent in situations where higher taxa are being compared, which has obvious implications for the positioning of *Plesiorycteropus* as well as many other extinct taxa. These results suggest that this mysterious *Plesiorycteropus* is likely a form of ‘giant’ tenrec, a diverse family that are now only represented by much smaller taxa. The results presented here demonstrate that fossil collagen (I) can be used to resolve such questions of taxonomy and, given its longevity over millions of years, has enormous potential to build a more complete Tree of Life than possible with DNA sequencing alone.

## Supporting Information

Figure S1 **MALDI-ToF-MS spectra showing the PMFs of collagen digests extracted from elephant shrew (** ***Petrodromus tetradactylus*** **), golden mole (** ***Ambylosomus hottentotus*** **), Sunda pangolin (** ***Manis javanica*** **), anteater (** ***Myrmecophaga tridactyla*** **) and hairy armadillo (** ***Chaeotophractus villosus*** **).**
(TIF)

Figure S2 **Tandem MS spectra of nine homologous peptides with precursor ions at** ***m/z*** **2161 (Sunda pangolin), 2147 (anteaters), 2129 (** ***Plesiorycteropus*** **), 2131 (hairy armadillo), 2089 (aardvark), 2073 (rock hyrax), 2145 (golden mole), 2161 (elephant shrew) and 2115 (elephantids) split into two windows of** ***m/z*** **range 200–1100 (A) and 1100–2000 (B).**
(TIF)

Figure S3 **Bayesian analysis of the 37 collagen sequences used in this study rooted to** ***Gallus*** **including the LC-MS-derived sequences.**
(TIF)

Table S1 **Mascot results for** ***Manis*** **bone acid-insoluble protein digest LC-MS data.**
(DOCX)

Table S2 **Mascot results for** ***Orycteropus*** **bone acid-insoluble protein digest LC-MS data.**
(DOCX)

Table S3 **Mascot results for** ***Tamandua*** **bone acid-insoluble protein digest LC-MS data.**
(DOCX)

Table S4 **Mascot results for** ***Petrodromus*** **skin acid-insoluble protein digest LC-MS data.**
(DOCX)

Table S5 **Mascot results for** ***Ambylosomus*** **bone acid-insoluble protein digest LC-MS data.**
(DOCX)

Table S6 **Mascot results for** ***Chaetophractus*** **bone acid-insoluble protein digest LC-MS data.**
(DOCX)

Table S7 **Mascot results for** ***Plesiorycteropus*** **bone acid-insoluble protein digest LC-MS data.**
(DOCX)

Table S8 **Mascot results for** ***Setifer*** **bone acid-insoluble protein digest LC-MS data.**
(DOCX)

Table S9 **Mascot results for** ***Procavia*** **bone acid-insoluble protein digest LC-MS data.**
(DOCX)

Table S10 **Mascot results for** ***Myrmecaphaga*** **bone acid-insoluble protein digest LC-MS data.**
(DOCX)

Table S11 **Mascot results for** ***Mammut*** **bone acid-insoluble protein digest LC-MS data.**
(DOCX)

Text S1 **Collagen (I) sequence accession numbers.**
(DOCX)

Text S2 **Concatenated collagen sequences.**
(DOCX)
