# Supplementary figures and images for: A Molecular Phylogeny of Plesiorycteropus Reassigns the Extinct Mammalian Order ‘Bibymalagasia’
Source: PLoS One. 2013 Mar 26;8(3):e59614. doi: 10.1371/journal.pone.0059614 (PMC3608660; doi:10.1371/journal.pone.0059614)

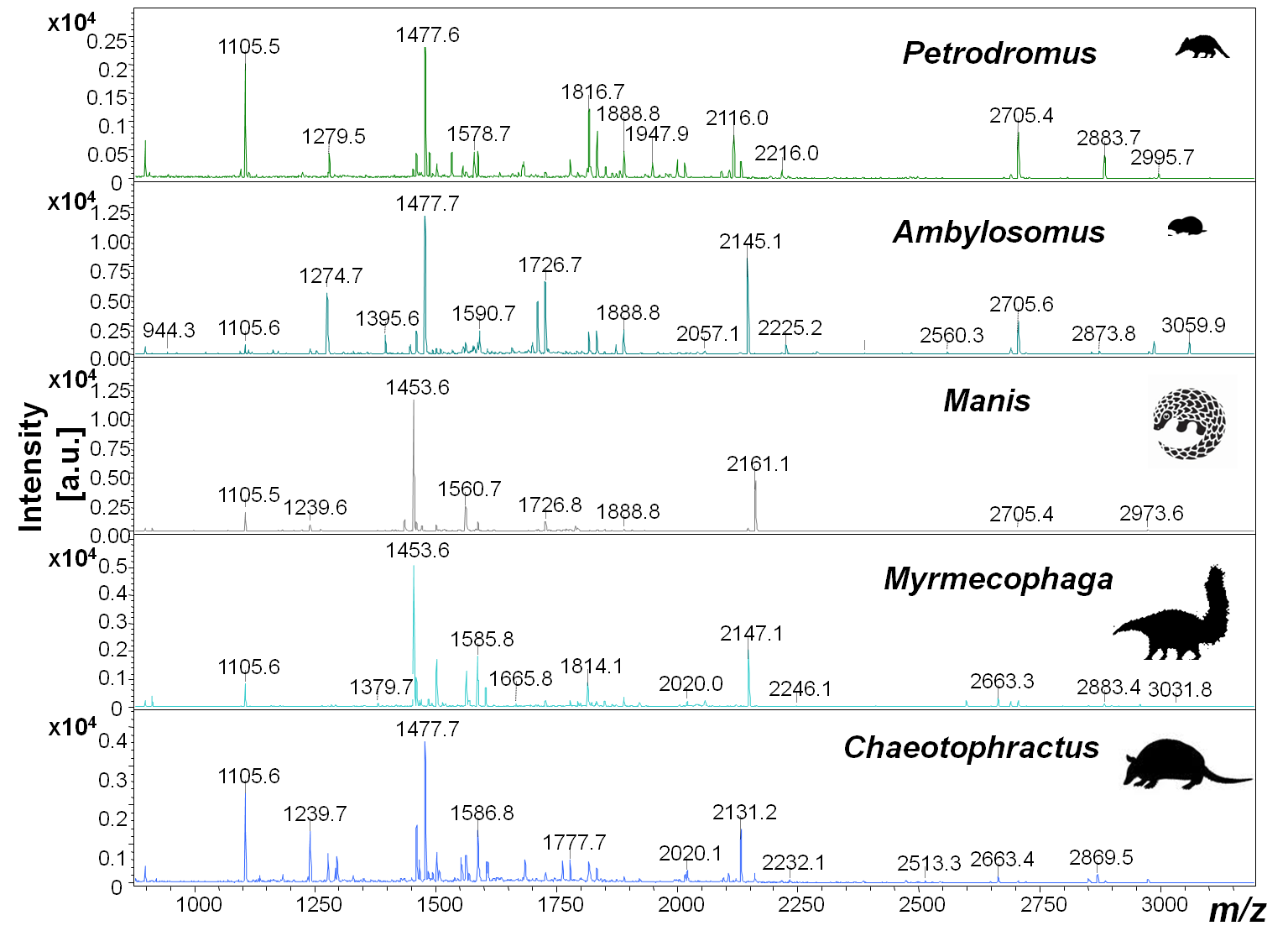

Supplement: Figure S1 — MALDI-ToF-MS spectra showing the PMFs of collagen digests extracted from elephant shrew ( Petrodromus tetradactylus ), golden mole ( Ambylosomus hottentotus ), Sunda pangolin ( Manis javanica ), anteater ( Myrmecophaga tridactyla ) and hairy armadillo ( Chaeotophractus villosus ). (TIF) [file pone.0059614.s001.tif]

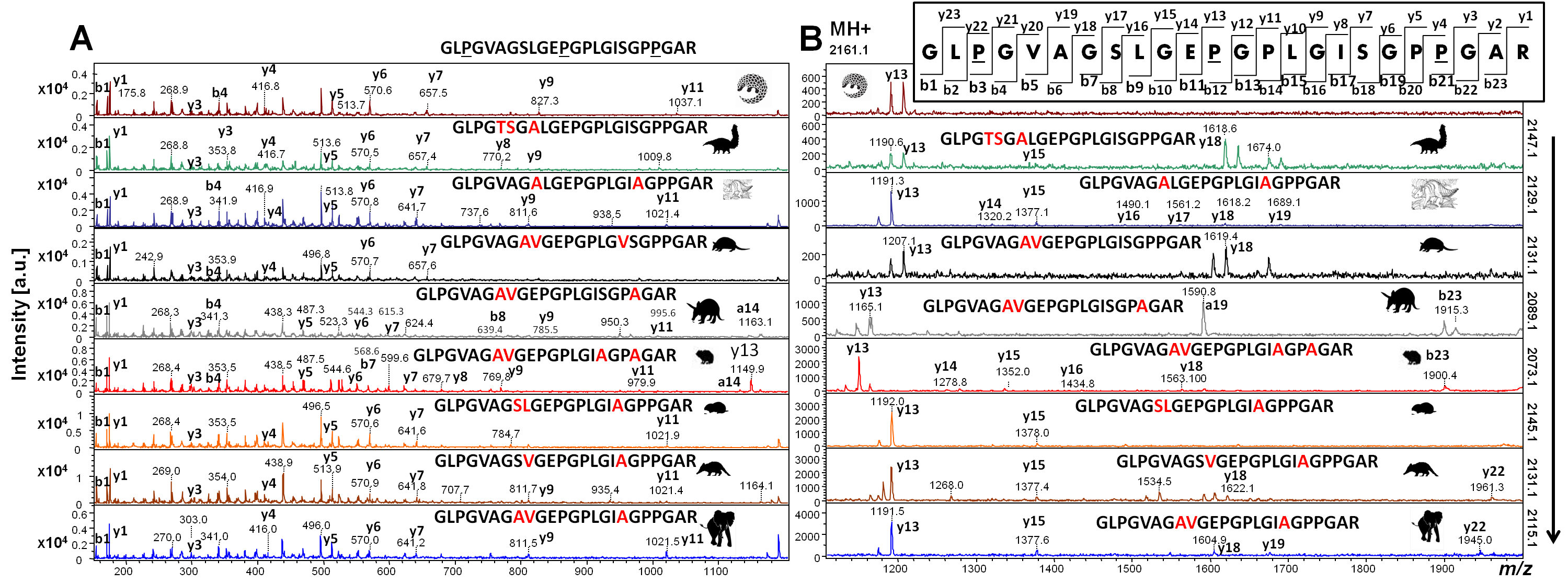

Supplement: Figure S2 — Tandem MS spectra of nine homologous peptides with precursor ions at m/z 2161 (Sunda pangolin), 2147 (anteaters), 2129 ( Plesiorycteropus ), 2131 (hairy armadillo), 2089 (aardvark), 2073 (rock hyrax), 2145 (golden mole), 2161 (elephant shrew) and 2115 (elephantids) split into two windows of m/z range 200–1100 (A) and 1100–2000 (B). (TIF) [file pone.0059614.s002.tif]

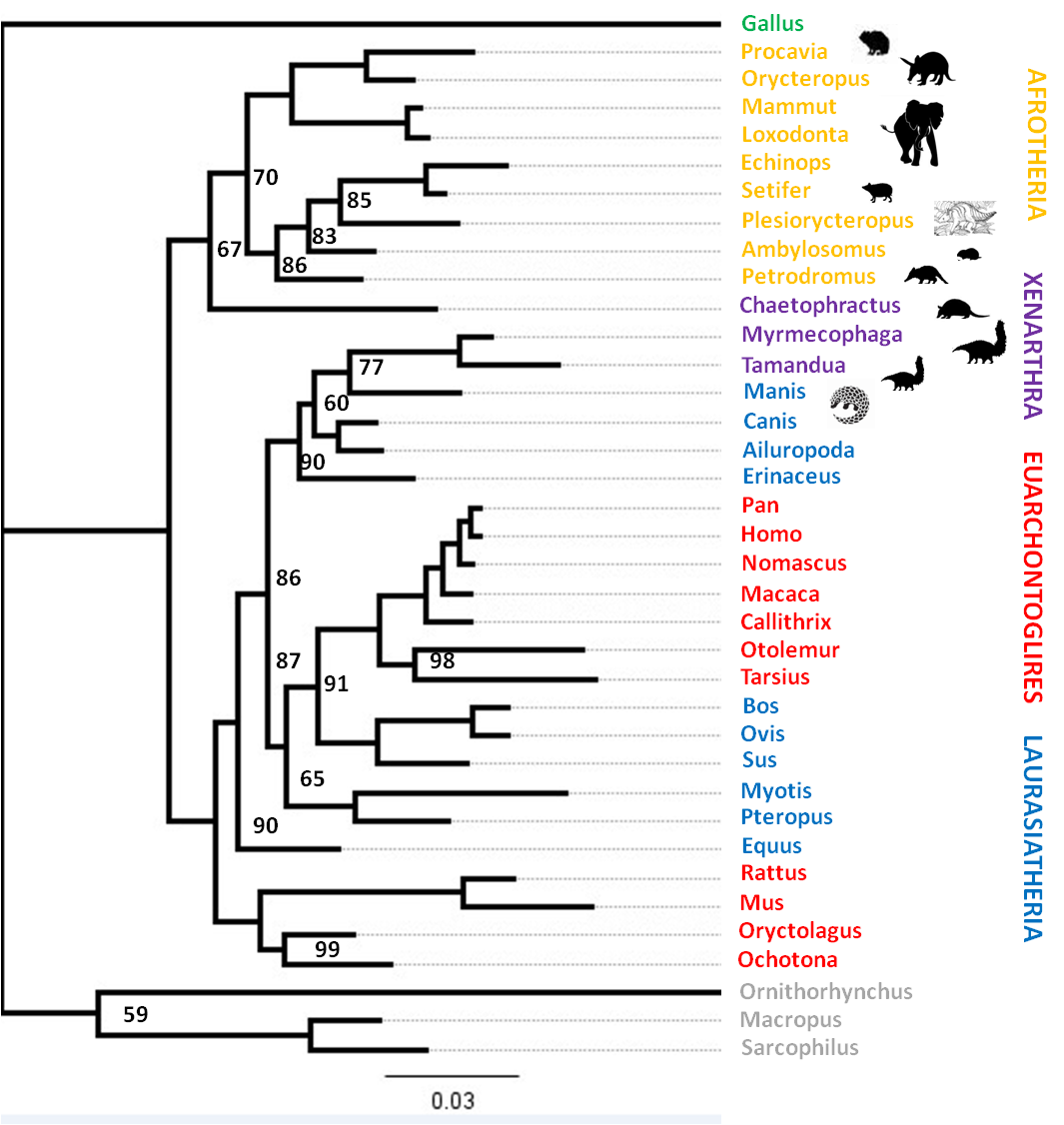

Supplement: Figure S3 — Bayesian analysis of the 37 collagen sequences used in this study rooted to Gallus including the LC-MS-derived sequences. (TIF) [file pone.0059614.s003.tif]
