# Supplementary material for: A Molecular Phylogeny of Plesiorycteropus Reassigns the Extinct Mammalian Order ‘Bibymalagasia’
Source: PLoS One. 2013 Mar 26;8(3):e59614. doi: 10.1371/journal.pone.0059614 (PMC3608660; doi:10.1371/journal.pone.0059614)
Supplement: Table S1 — Mascot results for Manis bone acid-insoluble protein digest LC-MS data. (DOCX) [file pone.0059614.s004.docx]

Table S1 – Mascot search results of LC-MS data against local database showing observed, expected and calculated molecular weights, the difference between expected and calculated molecular weights (Delta), the number of missed cleavages, peptide ion score, Expect score and peptide sequence (where underline represents modified amino acid) for *Manis* bone acid-insoluble protein digest.

| **Observed** | **Mr(expt)** | **Mr(calc)** | **Delta** | **Miss** | **Score** | **Expect** | **Peptide** |
| --- | --- | --- | --- | --- | --- | --- | --- |
| **392.2215** | **782.4284** | **782.4286** | **-0.0002** | **0** | **44** | **0.13** | **R.GAAGLPGPK.G** |
| **426.7385** | **851.4624** | **851.4250** | **0.0375** | **0** | **41** | **0.36** | **R.GPAGPQGPR.G** |
| **434.2135** | **866.4124** | **866.4134** | **-0.0010** | **0** | **42** | **0.25** | **R.GFSGLDGAK.G** |
| **449.7579** | **897.5012** | **897.5032** | **-0.0020** | **0** | **52** | **0.024** | **R.GVVGLPGQR.G** |
| **452.7548** | **903.4950** | **903.4927** | **0.0024** | **0** | **41** | **0.52** | **R.PGPVGPAGPR.G** |
| **469.2584** | **936.5022** | **936.5029** | **-0.0007** | **0** | **58** | **0.0078** | **R.GPAGPTGPVGK.D** |
| **499.7847** | **997.5548** | **997.4465** | **0.1083** | **0** | **57** | **0.0085** | **R.PGPPGPPGSR.G** |
| **529.7501** | **1057.4856** | **1057.4863** | **-0.0006** | **0** | **78** | **0.00011** | **R.PGEPGLMGPR.G** |
| **361.2001** | **1080.5785** | **1080.5788** | **-0.0004** | **1** | **40** | **0.48** | **R.GRVGAPGPAGAR.G** |
| **544.7720** | **1087.5294** | **1087.5298** | **-0.0004** | **0** | **53** | **0.033** | **R.GFPGADGVAGPK.G** |
| **545.2932** | **1088.5718** | **1088.5727** | **-0.0009** | **0** | **51** | **0.056** | [**R.GVQGPPGPAGPR.G**](http://msct.smith.man.ac.uk/mascot/cgi/peptide_view.pl?file=../data/20120829/F291555782.dat&query=697&hit=1&index=M00038&px=1&section=5&ave_thresh=52) |
| **550.7823** | **1099.5500** | **1099.5510** | **-0.0009** | **0** | **61** | **0.0047** | **R.GLVGEPGPAGSK.G** |
| **557.2679** | **1112.5212** | **1112.5211** | **0.0002** | **0** | **43** | **0.25** | **K.TGPTGPAGQDGR.P** |
| **588.8215** | **1175.6284** | **1175.6299** | **-0.0014** | **0** | **48** | **0.12** | **R.GVPGPPGAVGPAGK.D** |
| **589.2870** | **1176.5594** | **1176.5598** | **-0.0003** | **0** | **67** | **0.0013** | **R.GQAGVMGFPGPK.G** |
| **591.8091** | **1181.6036** | **1181.6041** | **-0.0004** | **0** | **47** | **0.1** | **K.EGPVGLPGIDGR.P** |
| **601.2956** | **1200.5766** | **1200.5775** | **-0.0009** | **0** | **57** | **0.012** | **R.GEPGNIGFPGPK.G** |
| **631.3180** | **1260.6214** | **1260.6211** | **0.0004** | **0** | **73** | **0.00035** | **R.GEAGPAGPAGPAGPR.G** |
| **637.3118** | **1272.6090** | **1272.6099** | **-0.0008** | **0** | **74** | **0.0003** | **R.GFPGSPGNVGPAGK.E** |
| **664.8280** | **1327.6414** | **1327.6409** | **0.0006** | **0** | **69** | **0.00082** | **R.GFPGLPGPSGEPGK.Q** |
| **666.8309** | **1331.6472** | **1331.6470** | **0.0002** | **0** | **53** | **0.037** | **R.GPSGPQGPSGPPGPK.G** |
| **675.8417** | **1349.6688** | **1349.6688** | **0.0001** | **1** | **53** | **0.034** | **K.GPAGERGSPGPAGPK.G** |
| **727.3753** | **1452.7360** | **1452.7361** | **-0.0001** | **0** | **70** | **0.00064** | **R.GLPGEFGLPGPAGPR.G** |
| **728.3508** | **1454.6870** | **1454.6903** | **-0.0032** | **0** | **79** | **8.1e-05** | **R.GDGGPPGAVGFPGAAGR.T** |
| **730.3499** | **1458.6852** | **1458.6852** | **0.0001** | **0** | **81** | **5.6e-05** | **R.GSAGPPGATGFPGAAGR.V** |
| **733.3491** | **1464.6836** | **1464.6845** | **-0.0009** | **0** | **53** | **0.033** | **R.GEPGPTGLPGPPGER.G** |
| **755.8444** | **1509.6742** | **1509.7172** | **-0.0430** | **0** | **48** | **0.11** | [**R.GAPGAVGAPGPAGATGDR.G**](http://msct.smith.man.ac.uk/mascot/cgi/peptide_view.pl?file=../data/20120829/F291555782.dat&query=2497&hit=1&index=M00038&px=1&section=5&ave_thresh=52) |
| **762.8438** | **1523.6730** | **1523.6740** | **-0.0010** | **0** | **57** | **0.016** | [**R.TGETGASGPPGFTGEK.G**](http://msct.smith.man.ac.uk/mascot/cgi/peptide_view.pl?file=../data/20120829/F291555782.dat&query=2564&hit=1&index=M00038&px=1&section=5&ave_thresh=52) |
| **775.3852** | **1548.7558** | **1548.7685** | **-0.0127** | **1** | **42** | **0.54** | [**R.GFPGTPGLPGFKGNR.G**](http://msct.smith.man.ac.uk/mascot/cgi/peptide_view.pl?file=../data/20120829/F291555782.dat&query=2691&hit=3&index=M00017&px=1&section=5&ave_thresh=52) |
| **780.9099** | **1559.8052** | **1559.8056** | **-0.0004** | **0** | **78** | **0.00013** | **R.GETGPAGPAGPIGPVGAR.G** |
| **781.8947** | **1561.7748** | **1561.7737** | **0.0012** | **0** | **55** | **0.027** | [**K.DGLNGLPGPIGPPGPR.G**](http://msct.smith.man.ac.uk/mascot/cgi/peptide_view.pl?file=../data/20120829/F291555782.dat&query=2755&hit=1&index=M00038&px=1&section=5&ave_thresh=52) |
| **781.9178** | **1561.8210** | **1561.8213** | **-0.0002** | **0** | **47** | **0.17** | **K.GAAGLPGVAGAPGLPGPR.G** |
| **783.8795** | **1565.7444** | **1565.7434** | **0.0010** | **0** | **72** | **0.00046** | **R.GPPGESGAAGPSGPIGSR.G** |
| **789.9089** | **1577.8032** | **1577.8050** | **-0.0017** | **0** | **72** | **0.00048** | **R.GLTGPIGPPGPAGATGDK.G** |
| **793.8817** | **1585.7488** | **1585.7485** | **0.0004** | **0** | **73** | **0.00036** | **K.GANGAPGIAGAPGFPGAR.G** |
| **535.9489** | **1604.8249** | **1604.8271** | **-0.0022** | **1** | **41** | **0.65** | **K.AGERGVPGPPGAVGPAGK.D** |
| **810.9055** | **1619.7964** | **1619.7904** | **0.0061** | **0** | **101** | **6.9e-07** | **K.GELGPVGNTGPSGPAGPR.G** |
| **828.4031** | **1654.7916** | **1654.7911** | **0.0006** | **1** | **54** | **0.029** | **K.GSPGEAGRPGEAGLPGAK.G** |
| **851.3694** | **1700.7242** | **1700.7238** | **0.0004** | **0** | **65** | **0.0025** | **K.GEPGAPGENGTPGQTGAR.G** |
| **853.8905** | **1705.7664** | **1705.7656** | **0.0009** | **0** | **93** | **3.9e-06** | **K.DGEAGAQGPPGPAGPAGER.G** |
| **870.4005** | **1738.7864** | **1738.7871** | **-0.0006** | **0** | **68** | **0.0015** | **R.GPSGGVGNPGVNGAPGEAGR.D** |
| **581.9549** | **1742.8429** | **1742.8449** | **-0.0020** | **1** | **51** | **0.075** | **K.GARGSAGPPGATGFPGAAGR.V** |
| **880.3445** | **1758.6744** | **1758.7115** | **-0.0371** | **0** | **51** | **0.074** | **K.GEPGSPGENGAPGQMGPR.G** |
| **588.9636** | **1763.8690** | **1763.8704** | **-0.0014** | **1** | **44** | **0.37** | **R.GFPGERGVQGPPGPAGPR.G** |
| **883.9190** | **1765.8234** | **1765.8231** | **0.0003** | **0** | **62** | **0.0047** | **K.PGEQGVPGDLGAPGPSGAR.G** |
| **899.3976** | **1796.7806** | **1796.7813** | **-0.0007** | **0** | **62** | **0.0053** | **K.GEPGSAGAQGPPGPSGEEGK.R** |
| **916.9381** | **1831.8616** | **1831.8523** | **0.0093** | **0** | **77** | **0.00017** | **R.GPPGPMGPPGLAGPPGESGR.E** |
| **917.4527** | **1832.8908** | **1832.8905** | **0.0003** | **0** | **81** | **7e-05** | **R.TGPPGPAGITGPPGPPGAAGK.E** |
| **921.4547** | **1840.8948** | **1840.8956** | **-0.0007** | **0** | **45** | **0.26** | **R.VGPPGPSGNAGPPGPPGPVGK.E** |
| **932.4401** | **1862.8656** | **1862.8647** | **0.0010** | **0** | **61** | **0.0077** | **K.GEPGPTGIQGPPGPAGEEGK.R** |
| **946.9410** | **1891.8674** | **1891.8660** | **0.0014** | **2** | **59** | **0.011** | **R.GPPGPPGKNGDDGEAGKPGR.P** |
| **977.4515** | **1952.8884** | **1952.8824** | **0.0060** | **1** | **44** | **0.4** | **K.GEPGSAGAQGPPGPSGEEGKR.G** |
| **1002.4740** | **2002.9334** | **2002.9821** | **-0.0486** | **1** | **42** | **0.62** | **K.NGDRGETGPAGPAGPIGPVGAR.G** |
| **1034.4940** | **2066.9734** | **2066.9658** | **0.0077** | **0** | **86** | **2.2e-05** | **R.GEVGPAGPNGFAGPAGAAGQPGAK.G** |
| **1053.0110** | **2104.0074** | **2103.9934** | **0.0141** | **0** | **58** | **0.014** | **K.GSPGADGPAGAPGTPGPQGIAGQR.G** |
| **1055.0130** | **2108.0114** | **2108.0135** | **-0.0020** | **0** | **88** | **1.5e-05** | **K.GEPGVLGAPGTAGASGPGGLPGER.G** |
| **1059.5050** | **2116.9954** | **2116.9814** | **0.0140** | **0** | **65** | **0.0029** | **R.GEPGPPGPAGFAGPPGADGQPGAK.G** |
| **1073.0700** | **2144.1254** | **2144.1226** | **0.0028** | **0** | **89** | **1.3e-05** | **R.GLPGVAGSLGEPGPLGISGPPGAR.G** |
| **1082.5050** | **2162.9954** | **2162.9465** | **0.0489** | **0** | **53** | **0.054** | **R.GAPGPDGNNGAQGPPGPQGVQGGK.G** |
| **1085.0320** | **2168.0494** | **2168.0498** | **-0.0004** | **0** | **74** | **0.0004** | **R.GETGPAGPPGAPGAPGAPGPVGPAGK.N** |
| **1108.4800** | **2214.9454** | **2214.9448** | **0.0007** | **0** | **66** | **0.0027** | **K.GDAGAPGAPGSQGAPGLQGMPGER.G** |
| **1141.0730** | **2280.1314** | **2280.1135** | **0.0179** | **0** | **55** | **0.028** | [**K.GDAGPAGPAGPAGPPGPIGNVGAPGPK.G**](http://msct.smith.man.ac.uk/mascot/cgi/peptide_view.pl?file=../data/20120829/F291555782.dat&query=6095&hit=1&index=M00038&px=1&section=5&ave_thresh=52) |
| **1158.5270** | **2315.0394** | **2315.0415** | **-0.0020** | **0** | **54** | **0.037** | **R.GEPGPPGPAGAAGPAGNPGADGQPGAK.G** |
| **1170.5440** | **2339.0734** | **2339.0666** | **0.0068** | **0** | **74** | **0.00043** | **R.GEQGPAGSPGFQGLPGPAGPSGEAGK.P** |
| **1172.0990** | **2342.1834** | **2342.1656** | **0.0179** | **0** | **53** | **0.046** | **R.GYPGNIGPVGAVGAPGPHGPVGPTGK.H** |
| **798.3953** | **2392.1641** | **2392.1296** | **0.0345** | **0** | **51** | **0.077** | **K.GEQGPAGPPGFQGLPGPAGPAGEVGK.P** |
| **1218.1060** | **2434.1974** | **2434.1976** | **-0.0002** | **0** | **100** | **9.7e-07** | **R.GEVGLPGLSGPVGPPGNPGANGLTGAK.G** |
| **829.7420** | **2486.2042** | **2486.2038** | **0.0004** | **1** | **43** | **0.51** | **R.GPPGSAGSPGKDGLNGLPGPIGPPGPR.G** |
| **1275.1050** | **2548.1954** | **2548.1831** | **0.0124** | **0** | **63** | **0.0055** | **R.GNDGATGAAGPPGPTGPAGPPGFPGAVGAK.G** |
| **1295.1340** | **2588.2534** | **2588.2508** | **0.0027** | **0** | **78** | **0.00015** | **R.GSDGSVGPVGPAGPIGSAGPPGFPGAPGPK.G** |
| **1300.1150** | **2598.2154** | **2598.1947** | **0.0208** | **0** | **108** | **1.8e-07** | **K.GENGPVGPTGPVGSAGPSGPNGPPGPAGSR.G** |
| **902.7568** | **2705.2486** | **2705.2318** | **0.0168** | **0** | **66** | **0.0024** | **R.GFSGLQGPPGPPGSPGEQGPSGASGPAGPR.G** |
| **950.4692** | **2848.3858** | **2848.3264** | **0.0594** | **1** | **49** | **0.12** | **K.GEQGPAGPPGFQGLPGPAGPAGEVGKPGER.G** |
| **958.4731** | **2872.3975** | **2872.3952** | **0.0023** | **1** | **49** | **0.14** | [**R.GLTGPIGPPGPAGATGDKGESGPSGPAGPTGAR.G**](http://msct.smith.man.ac.uk/mascot/cgi/peptide_view.pl?file=../data/20120829/F291555782.dat&query=7660&hit=1&index=M00038&px=1&section=5&ave_thresh=52) |
| **966.4598** | **2896.3576** | **2896.3588** | **-0.0012** | **1** | **71** | **0.0008** | **K.GPKGENGPVGPTGPVGSAGPSGPNGPPGPAGSR.G** |
| **1106.2240** | **3315.6502** | **3315.6332** | **0.0170** | **1** | **68** | **0.0016** | [**K.GPSGEPGTAGPPGTAGPQGLLGAPGILGLPGSRGER.G**](http://msct.smith.man.ac.uk/mascot/cgi/peptide_view.pl?file=../data/20120829/F291555782.dat&query=8386&hit=5&index=M00038&px=1&section=5&ave_thresh=52) |
