# Supplementary material for: A Molecular Phylogeny of Plesiorycteropus Reassigns the Extinct Mammalian Order ‘Bibymalagasia’
Source: PLoS One. 2013 Mar 26;8(3):e59614. doi: 10.1371/journal.pone.0059614 (PMC3608660; doi:10.1371/journal.pone.0059614)
Supplement: Table S2 — Mascot results for Orycteropus bone acid-insoluble protein digest LC-MS data. (DOCX) [file pone.0059614.s005.docx]

Table S2 – Mascot search results of LC-MS data against local database showing observed, expected and calculated molecular weights, the difference between expected and calculated molecular weights (Delta), the number of missed cleavages, peptide ion score, Expect score and peptide sequence (where underline represents modified amino acid) for *Orycteropus* bone acid-insoluble protein digest.

| **Observed** | **Mr(expt)** | **Mr(calc)** | **Delta** | **Miss** | **Score** | **Expect** | **Peptide** |
| --- | --- | --- | --- | --- | --- | --- | --- |
| **392.2216** | **782.4286** | **782.4286** | **-0.0000** | **0** | **44** | **0.12** | **R.GAAGLPGPK.G** |
| **449.7584** | **897.5022** | **897.5032** | **-0.0010** | **0** | **47** | **0.087** | [**R.GVVGLPGQR.G**](http://msct.smith.man.ac.uk/mascot/cgi/peptide_view.pl?file=../data/20120829/F291555796.dat&query=431&hit=1&index=M00045&px=1&section=5&ave_thresh=52) |
| **552.7689** | **1103.5232** | **1103.5248** | **-0.0015** | **0** | **49** | **0.089** | [**R.GFPGSDGVAGPK.G**](http://msct.smith.man.ac.uk/mascot/cgi/peptide_view.pl?file=../data/20120829/F291555796.dat&query=1063&hit=1&index=M00045&px=1&section=5&ave_thresh=52) |
| **553.7831** | **1105.5516** | **1105.5516** | **0.0000** | **0** | **50** | **0.062** | [**R.GVQGPPGPAGPR.G**](http://msct.smith.man.ac.uk/mascot/cgi/peptide_view.pl?file=../data/20120829/F291555796.dat&query=1075&hit=2&index=M00045&px=1&section=5&ave_thresh=52) |
| **581.3101** | **1160.6056** | **1160.6051** | **0.0006** | **0** | **50** | **0.064** | **R.SGHPGAVGPAGVR.G** |
| **589.7791** | **1177.5436** | **1177.5438** | **-0.0001** | **0** | **69** | **0.00079** | [**R.GQAGVMGFPGPK.G**](http://msct.smith.man.ac.uk/mascot/cgi/peptide_view.pl?file=../data/20120829/F291555796.dat&query=1342&hit=1&index=M00045&px=1&section=5&ave_thresh=52) |
| **601.2949** | **1200.5752** | **1200.5775** | **-0.0023** | **0** | **71** | **0.00057** | [**R.GEPGNIGFPGPK.G**](http://msct.smith.man.ac.uk/mascot/cgi/peptide_view.pl?file=../data/20120829/F291555796.dat&query=1454&hit=1&index=M00045&px=1&section=5&ave_thresh=52) |
| **611.8085** | **1221.6024** | **1221.6030** | **-0.0006** | **0** | **43** | **0.36** | **R.GFPGTPGLPGFK.G** |
| **620.3202** | **1238.6258** | **1238.6255** | **0.0003** | **0** | **76** | **0.00015** | **R.GLPGSPGNVGPAGK.E** |
| **629.7853** | **1257.5560** | **1257.5837** | **-0.0277** | **0** | **54** | **0.029** | **K.GLTGSPGSPGPDGK.T** |
| **634.3406** | **1266.6666** | **1266.6681** | **-0.0014** | **0** | **83** | **2.9e-05** | [**R.GIPGPVGAAGATGAR.G**](http://msct.smith.man.ac.uk/mascot/cgi/peptide_view.pl?file=../data/20120829/F291555796.dat&query=1781&hit=1&index=M00045&px=1&section=5&ave_thresh=52) |
| **639.3354** | **1276.6562** | **1276.6160** | **0.0402** | **0** | **84** | **2.6e-05** | [**R.GEAGPAGPAGPAGPR.G**](http://msct.smith.man.ac.uk/mascot/cgi/peptide_view.pl?file=../data/20120829/F291555796.dat&query=1847&hit=1&index=M00045&px=1&section=5&ave_thresh=52) |
| **653.8230** | **1305.6314** | **1305.6313** | **0.0001** | **0** | **64** | **0.0027** | [**R.GPSGPQGPSGAPGPK.G**](http://msct.smith.man.ac.uk/mascot/cgi/peptide_view.pl?file=../data/20120829/F291555796.dat&query=2008&hit=1&index=M00045&px=1&section=5&ave_thresh=52) |
| **672.8237** | **1343.6328** | **1343.6358** | **-0.0029** | **0** | **54** | **0.028** | [**R.GFPGLPGPSGEPGK.Q**](http://msct.smith.man.ac.uk/mascot/cgi/peptide_view.pl?file=../data/20120829/F291555796.dat&query=2282&hit=1&index=M00045&px=1&section=5&ave_thresh=52) |
| **714.3602** | **1426.7058** | **1426.7061** | **-0.0003** | **0** | **48** | **0.11** | **K.GVGLGPGPMGLMGPR.G** |
| **725.3494** | **1448.6842** | **1448.6896** | **-0.0053** | **0** | **57** | **0.014** | **R.GEPGPTGLPGPPGER.G** |
| **730.3489** | **1458.6832** | **1458.6852** | **-0.0019** | **0** | **56** | **0.018** | **R.GSAGPPGATGFPGAAGR.V** |
| **732.3734** | **1462.7322** | **1462.7317** | **0.0005** | **0** | **68** | **0.0012** | **R.GLHGDFGLPGPAGPR.G** |
| **741.8746** | **1481.7346** | **1481.7297** | **0.0050** | **0** | **43** | **0.4** | **K.SAGISVPGPMGPSGPR.G** |
| **762.8446** | **1523.6746** | **1523.6740** | **0.0006** | **0** | **54** | **0.03** | **R.TGETGASGPPGFTGEK.G** |
| **762.8710** | **1523.7274** | **1523.7328** | **-0.0054** | **0** | **55** | **0.022** | **R.GAPGAIGAPGPAGATGDR.G** |
| **780.9127** | **1559.8108** | **1559.8056** | **0.0052** | **0** | **61** | **0.0069** | [**R.GETGPAGPAGPIGPAGVR.G**](http://msct.smith.man.ac.uk/mascot/cgi/peptide_view.pl?file=../data/20120829/F291555796.dat&query=3450&hit=2&index=M00045&px=1&section=5&ave_thresh=52) |
| **521.2554** | **1560.7444** | **1560.7896** | **-0.0453** | **0** | **54** | **0.034** | **K.DGLNGLPGPIGPPGPR.G** |
| **781.9232** | **1561.8318** | **1561.8213** | **0.0106** | **0** | **73** | **0.00039** | [**K.GAAGLPGVAGAPGLPGPR.G**](http://msct.smith.man.ac.uk/mascot/cgi/peptide_view.pl?file=../data/20120829/F291555796.dat&query=3487&hit=1&index=M00045&px=1&section=5&ave_thresh=52) |
| **782.8945** | **1563.7744** | **1563.8046** | **-0.0301** | **1** | **55** | **0.022** | **R.GFPGTPGLPGFKGIR.G** |
| **793.8821** | **1585.7496** | **1585.7485** | **0.0012** | **0** | **67** | **0.0015** | **K.GANGAPGIAGAPGFPGAR.G** |
| **798.4109** | **1594.8072** | **1594.7700** | **0.0373** | **0** | **79** | **9.5e-05** | **R.GPPGQSGAAGPTGPIGSR.G** |
| **801.9201** | **1601.8256** | **1601.8162** | **0.0094** | **0** | **88** | **1.3e-05** | **R.GEPGPAGSVGPVGPVGPR.G** |
| **815.3965** | **1628.7784** | **1628.8159** | **-0.0374** | **1** | **42** | **0.53** | **R.GFSGLDGAKGDVGPAGPK.G** |
| **816.9013** | **1631.7880** | **1631.7904** | **-0.0023** | **0** | **78** | **0.00013** | **K.GELGPVGNPGPSGPAGPR.G** |
| **824.8999** | **1647.7852** | **1647.7853** | **-0.0000** | **0** | **53** | **0.04** | **R.GPNGEVGSAGPPGPPGLR.G** |
| **828.4008** | **1654.7870** | **1654.7911** | **-0.0040** | **1** | **59** | **0.01** | **K.GSPGEAGRPGEAGLPGAK.G** |
| **880.3674** | **1758.7202** | **1758.7115** | **0.0087** | **0** | **52** | **0.054** | **K.GEPGSPGENGAPGQMGPR.G** |
| **892.4232** | **1782.8318** | **1782.8795** | **-0.0477** | **1** | **41** | **0.73** | **R.AGVMGPPGSRGQTGPAGVR.G** |
| **906.9464** | **1811.8782** | **1811.8803** | **-0.0020** | **0** | **41** | **0.64** | **R.VGPPGPSGNAGPPGPPGPAGK.E** |
| **908.9371** | **1815.8596** | **1815.8574** | **0.0022** | **0** | **88** | **1.3e-05** | **R.GPPGPMGPPGLAGPPGESGR.E** |
| **909.4542** | **1816.8938** | **1816.8956** | **-0.0017** | **0** | **62** | **0.005** | [**R.TGPPGPSGITGPPGPPGAAGK.E**](http://msct.smith.man.ac.uk/mascot/cgi/peptide_view.pl?file=../data/20120829/F291555796.dat&query=5041&hit=1&index=M00045&px=1&section=5&ave_thresh=52) |
| **988.4972** | **1974.9798** | **1974.9872** | **-0.0073** | **1** | **67** | **0.0017** | **K.SGDRGETGPAGPAGPIGPAGVR.G** |
| **1021.5460** | **2041.0774** | **2041.0705** | **0.0069** | **1** | **49** | **0.12** | **K.EGPVGLPGIDGRPGPVGPAGAR.G** |
| **1026.4880** | **2050.9614** | **2050.9708** | **-0.0094** | **0** | **62** | **0.0064** | **R.GEVGPAGPNGFAGPAGAAGQPGAK.G** |
| **689.6872** | **2066.0398** | **2066.0406** | **-0.0008** | **1** | **57** | **0.017** | **K.HGNRGEPGPAGSVGPVGPVGPR.G** |
| **1045.0560** | **2088.0974** | **2088.0964** | **0.0011** | **0** | **66** | **0.0022** | **R.GLPGVAGAVGEPGPLGISGPAGAR.G** |
| **1053.9950** | **2105.9754** | **2105.9727** | **0.0028** | **0** | **65** | **0.0031** | **K.GSPGADGPAGAPGTPGPQGIGGQR.G** |
| **1093.0300** | **2184.0454** | **2184.0448** | **0.0007** | **0** | **59** | **0.011** | **R.GETGPAGPPGAPGAPGAPGPVGPAGK.S** |
| **1108.9720** | **2215.9294** | **2215.9288** | **0.0007** | **0** | **76** | **0.00026** | **K.GDAGAPGAPGSQGAPGLQGMPGER.G** |
| **1148.0770** | **2294.1394** | **2294.1292** | **0.0103** | **0** | **49** | **0.13** | **K.GDAGPPGPAGPTGAPGPIGNVGAPGPK.G** |
| **1219.6120** | **2437.2094** | **2437.2350** | **-0.0256** | **1** | **41** | **0.79** | **R.GPPGSAGAPGKDGLNGLPGPIGPPGPR.G** |
| **1274.6140** | **2547.2134** | **2547.1991** | **0.0144** | **0** | **110** | **1.1e-07** | **R.GNDGATGAAGPPGPTGPAGPPGFPGAVGAK.G** |
| **863.7570** | **2588.2492** | **2588.2508** | **-0.0016** | **0** | **48** | **0.19** | **R.GSDGSVGPVGPAGPIGSAGPPGFPGAPGPK.G** |
| **871.1155** | **2610.3247** | **2610.2787** | **0.0460** | **1** | **43** | **0.49** | **K.GDAGPPGPAGPTGAPGPIGNVGAPGPKGAR.G** |
| **884.4232** | **2650.2478** | **2650.2372** | **0.0106** | **1** | **50** | **0.12** | **R.GSPGERGEVGPAGPNGFAGPAGAAGQPGAK.G** |
| **897.0921** | **2688.2545** | **2688.2529** | **0.0016** | **0** | **52** | **0.063** | **R.GFSGLQGPPGPPGSPGEQGPSGASGPAGPR.G** |
| **907.0819** | **2718.2239** | **2718.2271** | **-0.0032** | **1** | **46** | **0.28** | **R.GAPGDRGEPGPPGPAGFAGPPGADGQPGAK.G** |
| **1042.8290** | **3125.4652** | **3125.4538** | **0.0114** | **1** | **44** | **0.4** | **K.GEPGDAGAKGDAGPPGPAGPTGAPGPIGNVGAPGPK.G** |
| **1071.5140** | **3211.5202** | **3211.5018** | **0.0183** | **1** | **54** | **0.048** | [**R.GPSGPPGPDGNKGEPGVVGAPGTAGPSGPSGLPGER.G**](http://msct.smith.man.ac.uk/mascot/cgi/peptide_view.pl?file=../data/20120829/F291555796.dat&query=7755&hit=1&index=M00045&px=1&section=5&ave_thresh=52) |
| **1110.2250** | **3327.6532** | **3327.6332** | **0.0200** | **1** | **60** | **0.011** | **K.GPSGEPGTAGPPGSPGPQGLLGAPGILGLPGSRGER.G** |
| **1138.8730** | **3413.5972** | **3413.6237** | **-0.0265** | **1** | **42** | **0.73** | **R.GNDGATGAAGPPGPTGPAGPPGFPGAVGAKGEVGPQGTR.G** |
