# Supplementary material for: A Molecular Phylogeny of Plesiorycteropus Reassigns the Extinct Mammalian Order ‘Bibymalagasia’
Source: PLoS One. 2013 Mar 26;8(3):e59614. doi: 10.1371/journal.pone.0059614 (PMC3608660; doi:10.1371/journal.pone.0059614)
Supplement: Table S3 — Mascot results for Tamandua bone acid-insoluble protein digest LC-MS data. (DOCX) [file pone.0059614.s006.docx]

Table S3 – Mascot search results of LC-MS data against local database showing observed, expected and calculated molecular weights, the difference between expected and calculated molecular weights (Delta), the number of missed cleavages, peptide ion score, Expect score and peptide sequence (where underline represents modified amino acid) for *Tamandua* bone acid-insoluble protein digest.

| **Observed** | **Mr(expt)** | **Mr(calc)** | **Delta** | **Miss** | **Score** | **Expect** | **Peptide** |
| --- | --- | --- | --- | --- | --- | --- | --- |
| **426.7377** | **851.4608** | **851.4250** | **0.0359** | **0** | **41** | **0.37** | **R.GPAGPQGPR.G** |
| **449.7585** | **897.5024** | **897.5032** | **-0.0008** | **0** | **52** | **0.026** | **R.GVVGLPGQR.G** |
| **521.7520** | **1041.4894** | **1041.4913** | **-0.0019** | **0** | **65** | **0.0017** | **R.PGEPGLMGPR.G** |
| **544.7722** | **1087.5298** | **1087.5298** | **0.0000** | **0** | **64** | **0.0023** | [**R.GFPGADGVAGPK.G**](http://msct.smith.man.ac.uk/mascot/cgi/peptide_view.pl?file=../data/20120829/F291555822.dat&query=756&hit=1&index=M00036&px=1&section=5&ave_thresh=52) |
| **553.2925** | **1104.5704** | **1104.5676** | **0.0028** | **0** | **50** | **0.069** | **R.GVQGPPGPAGPR.G** |
| **565.7996** | **1129.5846** | **1129.5802** | **0.0045** | **0** | **45** | **0.22** | **R.GLPGTAGLPGMK.G** |
| **589.7794** | **1177.5442** | **1177.5438** | **0.0005** | **0** | **68** | **0.0011** | **R.GQAGVMGFPGPK.G** |
| **591.8094** | **1181.6042** | **1181.6041** | **0.0002** | **0** | **40** | **0.5** | **K.EGPVGLPGIDGR.P** |
| **601.2961** | **1200.5776** | **1200.5775** | **0.0001** | **0** | **58** | **0.012** | [**R.GEPGNIGFPGPK.G**](http://msct.smith.man.ac.uk/mascot/cgi/peptide_view.pl?file=../data/20120829/F291555822.dat&query=1205&hit=1&index=M00036&px=1&section=5&ave_thresh=52) |
| **621.8012** | **1241.5878** | **1241.5888** | **-0.0010** | **0** | **69** | **0.00086** | **K.GLTGSPGSPGPDGK.T** |
| **664.8278** | **1327.6410** | **1327.6409** | **0.0002** | **0** | **88** | **1e-05** | **R.GFPGLPGPSGEPGK.Q** |
| **666.8306** | **1331.6466** | **1331.6470** | **-0.0004** | **0** | **67** | **0.0015** | **R.GPAGPQGPSGPPGPK.G** |
| **727.3752** | **1452.7358** | **1452.7361** | **-0.0003** | **0** | **65** | **0.0023** | **R.GLPGEFGLPGPAGPR.G** |
| **730.3500** | **1458.6854** | **1458.6852** | **0.0003** | **0** | **83** | **4.1e-05** | **R.GSAGPPGATGFPGAAGR.V** |
| **733.3484** | **1464.6822** | **1464.6845** | **-0.0023** | **0** | **55** | **0.022** | **R.GEPGPTGLPGPPGER.G** |
| **745.8708** | **1489.7270** | **1489.7274** | **-0.0003** | **0** | **68** | **0.0014** | [**R.GETGPAGPAGPAGPAGAR.G**](http://msct.smith.man.ac.uk/mascot/cgi/peptide_view.pl?file=../data/20120829/F291555822.dat&query=2446&hit=1&index=M00036&px=1&section=5&ave_thresh=52) |
| **751.3605** | **1500.7064** | **1500.6958** | **0.0107** | **0** | **64** | **0.003** | **R.GDGGPPGVTGFPGAAGR.T** |
| **766.8692** | **1531.7238** | **1531.7519** | **-0.0280** | **0** | **49** | **0.1** | [**R.TGELGAVGPPGFTGEK.G**](http://msct.smith.man.ac.uk/mascot/cgi/peptide_view.pl?file=../data/20120829/F291555822.dat&query=2700&hit=1&index=M00030&px=1&section=5&ave_thresh=52) |
| **781.8942** | **1561.7738** | **1561.7737** | **0.0002** | **0** | **64** | **0.003** | **K.DGLNGLPGPIGPPGPR.G** |
| **521.6139** | **1561.8199** | **1561.8213** | **-0.0014** | **0** | **48** | **0.12** | **K.GAAGLPGVAGAPGLPGPR.G** |
| **523.5926** | **1567.7560** | **1567.7591** | **-0.0031** | **0** | **69** | **0.00089** | **R.GPPGESGAVGPSGAIGSR.G** |
| **787.9133** | **1573.8120** | **1573.8100** | **0.0020** | **0** | **73** | **0.00037** | [**R.GLTGPIGPPGPAGAPGDK.G**](http://msct.smith.man.ac.uk/mascot/cgi/peptide_view.pl?file=../data/20120829/F291555822.dat&query=2933&hit=1&index=M00036&px=1&section=5&ave_thresh=52) |
| **793.8788** | **1585.7430** | **1585.7485** | **-0.0054** | **0** | **62** | **0.0049** | [**K.GANGAPGIAGAPGFPGAR.G**](http://msct.smith.man.ac.uk/mascot/cgi/peptide_view.pl?file=../data/20120829/F291555822.dat&query=3021&hit=1&index=M00036&px=1&section=5&ave_thresh=52) |
| **810.4103** | **1618.8060** | **1618.8064** | **-0.0003** | **0** | **94** | **3.4e-06** | **K.GELGPVGNTGPSGPAGPR.G** |
| **833.8868** | **1665.7590** | **1665.7595** | **-0.0004** | **0** | **53** | **0.044** | [**R.GPNGEPGSTGPSGPPGLR.G**](http://msct.smith.man.ac.uk/mascot/cgi/peptide_view.pl?file=../data/20120829/F291555822.dat&query=3460&hit=1&index=M00036&px=1&section=5&ave_thresh=52) |
| **840.8828** | **1679.7510** | **1679.7499** | **0.0011** | **0** | **71** | **0.00067** | **K.DGEAGAQGAPGPAGPAGER.G** |
| **875.9219** | **1749.8292** | **1749.8282** | **0.0010** | **0** | **74** | **0.00037** | **K.PGEQGVPGDLGAPGPSGAR.G** |
| **900.9380** | **1799.8614** | **1799.8625** | **-0.0010** | **0** | **96** | **2.4e-06** | [**R.GPPGPMGPPGLAGPPGESGR.E**](http://msct.smith.man.ac.uk/mascot/cgi/peptide_view.pl?file=../data/20120829/F291555822.dat&query=4017&hit=1&index=M00036&px=1&section=5&ave_thresh=52) |
| **932.4384** | **1862.8622** | **1862.8647** | **-0.0024** | **0** | **70** | **0.00083** | **K.GEPGPTGIQGPPGPAGEEGK.R** |
| **1053.0040** | **2103.9934** | **2103.9934** | **0.0001** | **0** | **67** | **0.002** | **K.GSPGADGPAGAPGTPGPQGISGQR.G** |
| **1054.0310** | **2106.0474** | **2106.0494** | **-0.0020** | **0** | **63** | **0.0049** | **R.GEAGPAGPPGAPGAPGAPGPVGPAGK.N** |
| **1055.0140** | **2108.0134** | **2108.0135** | **-0.0000** | **0** | **80** | **8.7e-05** | [**K.GEPGVLGAPGTAGASGPGGLPGER.G**](http://msct.smith.man.ac.uk/mascot/cgi/peptide_view.pl?file=../data/20120829/F291555822.dat&query=5005&hit=1&index=M00036&px=1&section=5&ave_thresh=52) |
| **1066.0620** | **2130.1094** | **2130.1069** | **0.0025** | **0** | **77** | **0.0002** | **R.GLPGTSGALGEPGPLGISGPPGAR.G** |
| **1074.4830** | **2146.9514** | **2146.9516** | **-0.0001** | **0** | **70** | **0.00092** | **R.GAPGPDGNNGAQGPPGPQGVQGGK.G** |
| **1075.4970** | **2148.9794** | **2148.9713** | **0.0082** | **0** | **65** | **0.0034** | [**R.GEPGPPGPAGFAGPPGADGQPGAK.G**](http://msct.smith.man.ac.uk/mascot/cgi/peptide_view.pl?file=../data/20120829/F291555822.dat&query=5230&hit=9&index=M00036&px=1&section=5&ave_thresh=52) |
| **1107.9870** | **2213.9594** | **2213.9608** | **-0.0013** | **0** | **52** | **0.066** | [**K.GDAGAPGAPGSQGAPGLQGMPGER.G**](http://msct.smith.man.ac.uk/mascot/cgi/peptide_view.pl?file=../data/20120829/F291555822.dat&query=5541&hit=1&index=M00036&px=1&section=5&ave_thresh=52) |
| **1137.5230** | **2273.0314** | **2273.0309** | **0.0006** | **0** | **57** | **0.02** | **R.GEPGPPGPAGAAGPAGNPGADGQAGAK.G** |
| **1154.0910** | **2306.1674** | **2306.1292** | **0.0383** | **0** | **55** | **0.033** | [**K.GDAGPPGPAGPSGPPGPIGNVGAPGPK.G**](http://msct.smith.man.ac.uk/mascot/cgi/peptide_view.pl?file=../data/20120829/F291555822.dat&query=5718&hit=1&index=M00036&px=1&section=5&ave_thresh=52) |
| **1183.0670** | **2364.1194** | **2364.0983** | **0.0212** | **0** | **81** | **7.3e-05** | [**R.GEQGPAGSPGFQGLPGPAGPPGEAGK.P**](http://msct.smith.man.ac.uk/mascot/cgi/peptide_view.pl?file=../data/20120829/F291555822.dat&query=5828&hit=3&index=M00036&px=1&section=5&ave_thresh=52) |
| **1198.0810** | **2394.1474** | **2394.1452** | **0.0022** | **0** | **69** | **0.0012** | [**K.GEQGPAGPPGFQGLPGPAGTTGEVGK.P**](http://msct.smith.man.ac.uk/mascot/cgi/peptide_view.pl?file=../data/20120829/F291555822.dat&query=5891&hit=1&index=M00036&px=1&section=5&ave_thresh=52) |
| **1274.6170** | **2547.2194** | **2547.1991** | **0.0204** | **0** | **93** | **5.1e-06** | **R.GNDGATGAAGPPGPTGPAGPPGFPGAVGAK.G** |
| **1300.1480** | **2598.2814** | **2598.2310** | **0.0504** | **0** | **86** | **2.9e-05** | [**K.GENGVVGPTGPIGAAGPSGPNGPPGPAGSR.G**](http://msct.smith.man.ac.uk/mascot/cgi/peptide_view.pl?file=../data/20120829/F291555822.dat&query=6194&hit=2&index=M00036&px=1&section=5&ave_thresh=52) |
| **1303.1320** | **2604.2494** | **2604.2457** | **0.0038** | **0** | **63** | **0.0057** | **R.GSDGSVGPVGPAGPIGSAGPPGFPGAPGPK.G** |
| **888.7465** | **2663.2177** | **2663.2212** | **-0.0036** | **0** | **56** | **0.029** | **R.GFSGLQGPPGAPGSPGEQGPSGASGPAGPR.G** |
| **950.7928** | **2849.3566** | **2849.3581** | **-0.0015** | **1** | **54** | **0.048** | **K.GEQGPAGPPGFQGLPGPAGTTGEVGKPGER.G** |
| **966.1577** | **2895.4513** | **2895.4111** | **0.0401** | **1** | **48** | **0.16** | **K.GPKGENGVVGPTGPIGAAGPSGPNGPPGPAGSR.G** |
