# Supplementary material for: A Molecular Phylogeny of Plesiorycteropus Reassigns the Extinct Mammalian Order ‘Bibymalagasia’
Source: PLoS One. 2013 Mar 26;8(3):e59614. doi: 10.1371/journal.pone.0059614 (PMC3608660; doi:10.1371/journal.pone.0059614)
Supplement: Table S4 — Mascot results for Petrodromus skin acid-insoluble protein digest LC-MS data. (DOCX) [file pone.0059614.s007.docx]

Table S4 – Mascot search results of LC-MS data against local database showing observed, expected and calculated molecular weights, the difference between expected and calculated molecular weights (Delta), the number of missed cleavages, peptide ion score, Expect score and peptide sequence (where underline represents modified amino acid) for *Petrodromus* bone acid-insoluble protein digest.

| **Observed** | **Mr(expt)** | **Mr(calc)** | **Delta** | **Miss** | **Score** | **Expect** | **Peptide** |
| --- | --- | --- | --- | --- | --- | --- | --- |
| **379.6933** | **757.3720** | **757.3719** | **0.0002** | **0** | **44** | **0.17** | **R.GLPGADGR.A** |
| **392.2218** | **782.4290** | **782.4286** | **0.0004** | **0** | **51** | **0.025** | [**R.GAAGLPGPK.G**](http://msct.smith.man.ac.uk/mascot/cgi/peptide_view.pl?file=../data/20120830/F291555843.dat&query=186&hit=1&index=M00075&px=1&section=5&ave_thresh=52) |
| **393.2170** | **784.4194** | **784.4192** | **0.0003** | **0** | **40** | **0.36** | **R.GATGPAGVR.G** |
| **426.2169** | **850.4192** | **850.4185** | **0.0008** | **0** | **42** | **0.31** | [**R.GFSGLDGAK.G**](http://msct.smith.man.ac.uk/mascot/cgi/peptide_view.pl?file=../data/20120830/F291555843.dat&query=421&hit=1&index=M00075&px=1&section=5&ave_thresh=52) |
| **441.7615** | **881.5084** | **881.5083** | **0.0001** | **0** | **50** | **0.039** | **R.GVVGLPGQR.G** |
| **472.7346** | **943.4546** | **943.4546** | **0.0001** | **0** | **62** | **0.0037** | **R.AGVMGPPGSR.G** |
| **499.7849** | **997.5552** | **997.4465** | **0.1087** | **0** | **49** | **0.062** | **R.PGPPGPPGSR.G** |
| **529.7379** | **1057.4612** | **1057.4863** | **-0.0250** | **0** | **42** | **0.41** | **R.PGEPGLMGPR.G** |
| **544.7724** | **1087.5302** | **1087.5298** | **0.0004** | **0** | **65** | **0.0023** | **R.GFPGADGVAGPK.G** |
| **545.7855** | **1089.5564** | **1089.5567** | **-0.0003** | **0** | **47** | **0.14** | **R.GVQGPPGPAGPR.G** |
| **564.2945** | **1126.5744** | **1126.5731** | **0.0014** | **1** | **40** | **0.51** | **R.GAAGLPGPKGDR.G** |
| **579.2627** | **1156.5108** | **1156.4745** | **0.0363** | **0** | **52** | **0.038** | **R.DGNPGSDGPPGR.D** |
| **581.2899** | **1160.5652** | **1160.5648** | **0.0004** | **0** | **75** | **0.00021** | [**R.GQAGVMGFPGPK.G**](http://msct.smith.man.ac.uk/mascot/cgi/peptide_view.pl?file=../data/20120830/F291555843.dat&query=2125&hit=1&index=M00075&px=1&section=5&ave_thresh=52) |
| **581.7916** | **1161.5686** | **1161.5700** | **-0.0013** | **0** | **46** | **0.16** | **R.GLPGTAGLPGMK.G** |
| **601.2961** | **1200.5776** | **1200.5775** | **0.0001** | **0** | **71** | **0.00057** | **R.GEPGNIGFPGPK.G** |
| **618.3105** | **1234.6064** | **1234.6054** | **0.0010** | **0** | **62** | **0.0039** | [**R.GEAGAAGPAGPAGPR.G**](http://msct.smith.man.ac.uk/mascot/cgi/peptide_view.pl?file=../data/20120830/F291555843.dat&query=2611&hit=1&index=M00075&px=1&section=5&ave_thresh=52) |
| **620.3204** | **1238.6262** | **1238.6255** | **0.0007** | **0** | **75** | **0.00018** | [**R.GLPGSPGNVGPAGK.E**](http://msct.smith.man.ac.uk/mascot/cgi/peptide_view.pl?file=../data/20120830/F291555843.dat&query=2643&hit=1&index=M00075&px=1&section=5&ave_thresh=52) |
| **629.7997** | **1257.5848** | **1257.5837** | **0.0011** | **0** | **50** | **0.07** | **K.GLTGSPGSPGPDGK.T** |
| **653.8235** | **1305.6324** | **1305.6313** | **0.0011** | **0** | **77** | **0.00015** | **R.GPSGPQGPSGAPGPK.G** |
| **656.3193** | **1310.6240** | **1310.6215** | **0.0025** | **0** | **67** | **0.0013** | **K.GETGPSGPAGPTGAR.G** |
| **664.8281** | **1327.6416** | **1327.6409** | **0.0008** | **0** | **65** | **0.0021** | **R.GFPGLPGPSGEPGK.Q** |
| **670.3342** | **1338.6538** | **1338.6537** | **0.0002** | **0** | **77** | **0.00015** | **K.GVGGGPGPMGLMGPR.G** |
| **722.3536** | **1442.6926** | **1442.6903** | **0.0024** | **0** | **85** | **2.2e-05** | **R.GSAGPPGATGFPGAAGR.V** |
| **733.3504** | **1464.6862** | **1464.6845** | **0.0017** | **0** | **64** | **0.0027** | [**R.GEPGPTGLPGPPGER.G**](http://msct.smith.man.ac.uk/mascot/cgi/peptide_view.pl?file=../data/20120830/F291555843.dat&query=4008&hit=1&index=M00075&px=1&section=5&ave_thresh=52) |
| **734.8649** | **1467.7152** | **1467.7140** | **0.0012** | **0** | **64** | **0.0026** | **K.SAGVSVPGPMGPSGPR.G** |
| **737.8627** | **1473.7108** | **1473.7100** | **0.0009** | **0** | **51** | **0.069** | **R.PGEVGPPGPPGPAGEK.G** |
| **739.3815** | **1476.7484** | **1476.7474** | **0.0011** | **0** | **60** | **0.0085** | **R.GLHGEFGLPGPAGPR.G** |
| **739.8267** | **1477.6388** | **1477.6685** | **-0.0297** | **0** | **65** | **0.0022** | [**R.TGETGASGPPGFAGEK.G**](http://msct.smith.man.ac.uk/mascot/cgi/peptide_view.pl?file=../data/20120830/F291555843.dat&query=4100&hit=1&index=M00075&px=1&section=5&ave_thresh=52) |
| **755.8666** | **1509.7186** | **1509.7172** | **0.0014** | **0** | **80** | **7.1e-05** | **R.GAPGAVGAPGPAGATGDR.G** |
| **766.8956** | **1531.7766** | **1531.7743** | **0.0023** | **0** | **76** | **0.00019** | **R.GETGPAGPAGPIGPAGAR.G** |
| **773.8971** | **1545.7796** | **1545.7787** | **0.0009** | **0** | **72** | **0.00053** | [**K.DGLNGLPGPIGPPGPR.G**](http://msct.smith.man.ac.uk/mascot/cgi/peptide_view.pl?file=../data/20120830/F291555843.dat&query=4645&hit=1&index=M00075&px=1&section=5&ave_thresh=52) |
| **778.3947** | **1554.7748** | **1554.7387** | **0.0362** | **0** | **72** | **0.00048** | **R.GPPGQSGAAGPTGGIGSR.G** |
| **789.8967** | **1577.7788** | **1577.7798** | **-0.0010** | **0** | **88** | **1.3e-05** | **R.GEPGPAGSVGPTGAVGPR.G** |
| **793.8826** | **1585.7506** | **1585.7485** | **0.0022** | **0** | **79** | **8.9e-05** | **K.GANGAPGIAGAPGFPGAR.G** |
| **795.9111** | **1589.8076** | **1589.8050** | **0.0027** | **0** | **57** | **0.015** | [**R.GLTGPIGPPGPAGAPGDK.G**](http://msct.smith.man.ac.uk/mascot/cgi/peptide_view.pl?file=../data/20120830/F291555843.dat&query=5011&hit=4&index=M00075&px=1&section=5&ave_thresh=52) |
| **809.3974** | **1616.7802** | **1616.7795** | **0.0008** | **1** | **85** | **2.5e-05** | **R.GFSGLDGAKGDAGPAGPK.G** |
| **816.4110** | **1630.8074** | **1630.8064** | **0.0011** | **0** | **81** | **7.2e-05** | **K.GELGPVGNPGPSGPAGPR.G** |
| **828.4041** | **1654.7936** | **1654.7911** | **0.0026** | **1** | **71** | **0.00064** | **K.GSPGEAGRPGEAGLPGAK.G** |
| **843.9419** | **1685.8692** | **1685.8697** | **-0.0004** | **2** | **48** | **0.12** | **R.GAAGIPGGKGEKGETGLR.G** |
| **853.8909** | **1705.7672** | **1705.7656** | **0.0017** | **0** | **85** | **2.9e-05** | **K.DGEAGAQGPPGPAGPAGER.G** |
| **862.9331** | **1723.8516** | **1723.8489** | **0.0027** | **1** | **58** | **0.012** | **R.GHNGLDGLKGQAGAPGVK.G** |
| **872.3662** | **1742.7178** | **1742.7166** | **0.0012** | **0** | **75** | **0.0003** | **K.GEPGSPGENGAPGQMGPR.G** |
| **581.9554** | **1742.8444** | **1742.8449** | **-0.0005** | **1** | **45** | **0.27** | **K.GARGSAGPPGATGFPGAAGR.V** |
| **890.4177** | **1778.8208** | **1778.8184** | **0.0025** | **0** | **82** | **5.8e-05** | [**R.GPPGAVGNPGVNGAPGEAGR.D**](http://msct.smith.man.ac.uk/mascot/cgi/peptide_view.pl?file=../data/20120830/F291555843.dat&query=6078&hit=1&index=M00075&px=1&section=5&ave_thresh=52) |
| **908.9370** | **1815.8594** | **1815.8574** | **0.0020** | **0** | **104** | **3.4e-07** | **R.GPPGPMGPPGLAGPPGESGR.E** |
| **910.4282** | **1818.8418** | **1818.8384** | **0.0034** | **0** | **65** | **0.0031** | [**K.GEPGPAGVQGPPGPAGEEGK.R**](http://msct.smith.man.ac.uk/mascot/cgi/peptide_view.pl?file=../data/20120830/F291555843.dat&query=6334&hit=1&index=M00075&px=1&section=5&ave_thresh=52) |
| **914.9460** | **1827.8774** | **1827.8752** | **0.0023** | **0** | **51** | **0.076** | **R.VGPPGPSGNAGPPGPPGPAGK.E** |
| **939.4233** | **1876.8320** | **1876.8300** | **0.0020** | **1** | **44** | **0.36** | **R.DGNPGSDGPPGRDGLPGHK.G** |
| **965.4382** | **1928.8618** | **1928.8873** | **-0.0255** | **1** | **42** | **0.51** | **R.QYDGKGVGGGPGPMGLMGPR.G** |
| **974.4857** | **1946.9568** | **1946.9559** | **0.0010** | **1** | **79** | **0.00012** | **K.SGDRGETGPAGPAGPIGPAGAR.G** |
| **988.4547** | **1974.8948** | **1974.9395** | **-0.0447** | **1** | **54** | **0.035** | **K.GEPGPAGVQGPPGPAGEEGKR.G** |
| **990.4754** | **1978.9362** | **1978.8981** | **0.0382** | **1** | **53** | **0.045** | **K.GEPGSAGPQGPPGPSGEEGKR.G** |
| **682.0044** | **2042.9914** | **2042.9882** | **0.0031** | **1** | **68** | **0.0015** | [**K.HGNRGEPGPAGSVGPTGAVGPR.G**](http://msct.smith.man.ac.uk/mascot/cgi/peptide_view.pl?file=../data/20120830/F291555843.dat&query=7664&hit=1&index=M00075&px=1&section=5&ave_thresh=52) |
| **1060.9840** | **2119.9534** | **2119.9519** | **0.0015** | **0** | **66** | **0.0024** | **R.GAPGPDGNNGAQGSPGPQGVQGGK.G** |
| **1061.9930** | **2121.9714** | **2121.9676** | **0.0039** | **0** | **63** | **0.0046** | [**K.GSPGADGPAGAPGTPGPQGIGGQR.G**](http://msct.smith.man.ac.uk/mascot/cgi/peptide_view.pl?file=../data/20120830/F291555843.dat&query=8062&hit=1&index=M00075&px=1&section=5&ave_thresh=52) |
| **1066.0630** | **2130.1114** | **2130.1070** | **0.0045** | **0** | **100** | **9.1e-07** | **R.GLPGVAGSVGEPGPLGIAGPPGAR.G** |
| **1069.0380** | **2136.0614** | **2136.0600** | **0.0014** | **0** | **75** | **0.0003** | [**R.GETGPAGPPGAPGAPGAPGPVGPAGK.S**](http://msct.smith.man.ac.uk/mascot/cgi/peptide_view.pl?file=../data/20120830/F291555843.dat&query=8161&hit=1&index=M00075&px=1&section=5&ave_thresh=52) |
| **1100.5380** | **2199.0614** | **2199.0556** | **0.0058** | **1** | **50** | **0.11** | **R.GETGPAGRPGEVGPPGPPGPAGEK.G** |
| **1142.5350** | **2283.0554** | **2283.0516** | **0.0038** | **0** | **75** | **0.00032** | [**R.GEPGPPGPAGAAGPAGNPGADGQPGAK.G**](http://msct.smith.man.ac.uk/mascot/cgi/peptide_view.pl?file=../data/20120830/F291555843.dat&query=8681&hit=1&index=M00075&px=1&section=5&ave_thresh=52) |
| **1149.5570** | **2297.0994** | **2297.1288** | **-0.0294** | **0** | **52** | **0.063** | **K.GDAGPPGPAGPTGAPGPIGNVGAPGVK.G** |
| **1175.0610** | **2348.1074** | **2348.1033** | **0.0041** | **0** | **67** | **0.0019** | **R.GEQGPAGSPGFQGLPGPAGPPGEAGK.P** |
| **1176.0700** | **2350.1254** | **2350.1190** | **0.0064** | **0** | **61** | **0.0077** | **K.GEQGPAGPPGFQGLPGPAGTGGEVGK.P** |
| **1218.5930** | **2435.1714** | **2435.1677** | **0.0037** | **2** | **42** | **0.72** | **R.GSPGPAGPKGSPGEAGRPGEAGLPGAK.G** |
| **819.0798** | **2454.2176** | **2454.2139** | **0.0036** | **1** | **68** | **0.0017** | **R.GPPGSAGSPGKDGLNGLPGPIGPPGPR.G** |
| **1257.1060** | **2512.1974** | **2512.1943** | **0.0032** | **1** | **101** | **7.6e-07** | [**K.GDRGETGPAGPPGAPGAPGAPGPVGPAGK.S**](http://msct.smith.man.ac.uk/mascot/cgi/peptide_view.pl?file=../data/20120830/F291555843.dat&query=9346&hit=1&index=M00075&px=1&section=5&ave_thresh=52) |
| **842.7026** | **2525.0860** | **2525.0725** | **0.0135** | **1** | **51** | **0.089** | **K.GDAGPAGPKGEPGSPGENGAPGQMGPR.G** |
| **1267.1140** | **2532.2134** | **2532.1881** | **0.0253** | **0** | **86** | **2.6e-05** | **R.GNDGATGAAGPPGPTGPAGPPGFPGAVGAK.G** |
| **1287.1370** | **2572.2594** | **2572.2558** | **0.0036** | **0** | **77** | **0.00022** | **R.GSDGSVGPVGPAGPIGSAGPPGFPGAPGPK.G** |
| **1345.1360** | **2688.2574** | **2688.2529** | **0.0046** | **0** | **96** | **2.4e-06** | **R.GFSGLQGPPGPPGSPGEQGPSGASGPAGPR.G** |
| **909.7791** | **2726.3155** | **2726.3121** | **0.0034** | **1** | **48** | **0.17** | **R.GAPGAVGAPGPAGATGDRGEAGAAGPAGPAGPR.G** |
| **936.4476** | **2806.3210** | **2806.3159** | **0.0051** | **1** | **59** | **0.013** | **K.GEQGPAGPPGFQGLPGPAGTGGEVGKPGER.G** |
| **1416.1850** | **2830.3554** | **2830.3496** | **0.0059** | **0** | **59** | **0.013** | **K.GHNGLQGLPGLAGHHGDQGAPGTVGPAGPR.G** |
| **960.4758** | **2878.4056** | **2878.3958** | **0.0097** | **1** | **43** | **0.49** | **R.GVPGPPGAVGQAGKDGEAGAQGPPGPAGPAGER.G** |
| **1442.2180** | **2882.4214** | **2882.4159** | **0.0055** | **1** | **71** | **0.00078** | [**R.GLTGPIGPPGPAGAPGDKGETGPSGPAGPTGAR.G**](http://msct.smith.man.ac.uk/mascot/cgi/peptide_view.pl?file=../data/20120830/F291555843.dat&query=10272&hit=1&index=M00075&px=1&section=5&ave_thresh=52) |
| **973.7704** | **2918.2894** | **2918.2663** | **0.0230** | **1** | **45** | **0.35** | **R.GPPGAVGNPGVNGAPGEAGRDGNPGSDGPPGR.D** |
| **1485.2520** | **2968.4894** | **2968.4891** | **0.0004** | **0** | **42** | **0.76** | **K.GPSGEPGTAGPPGSPGPQGLLGAPGILGLPGSR.G** |
| **1109.8920** | **3326.6542** | **3326.6492** | **0.0050** | **1** | **72** | **0.00065** | [**K.GPSGEPGTAGPPGSPGPQGLLGAPGILGLPGSRGER.G**](http://msct.smith.man.ac.uk/mascot/cgi/peptide_view.pl?file=../data/20120830/F291555843.dat&query=10938&hit=2&index=M00075&px=1&section=5&ave_thresh=52) |
| **1129.8720** | **3386.5942** | **3386.5876** | **0.0065** | **1** | **45** | **0.35** | **R.GNDGATGAAGPPGPTGPAGPPGFPGAVGAKGEAGPQGAR.G** |
| **1138.5780** | **3412.7122** | **3412.6873** | **0.0249** | **1** | **51** | **0.082** | **R.GLPGLKGHNGLQGLPGLAGHHGDQGAPGTVGPAGPR.G** |
