# Supplementary material for: A Molecular Phylogeny of Plesiorycteropus Reassigns the Extinct Mammalian Order ‘Bibymalagasia’
Source: PLoS One. 2013 Mar 26;8(3):e59614. doi: 10.1371/journal.pone.0059614 (PMC3608660; doi:10.1371/journal.pone.0059614)
Supplement: Table S5 — Mascot results for Ambylosomus bone acid-insoluble protein digest LC-MS data. (DOCX) [file pone.0059614.s008.docx]

Table S5 - Mascot search results of LC-MS data against local database showing observed, expected and calculated molecular weights, the difference between expected and calculated molecular weights (Delta), the number of missed cleavages, peptide ion score, Expect score and peptide sequence (where underline represents modified amino acid) for *Ambylosomus* bone acid-insoluble protein digest.

| **Observed** | **Mr(expt)** | **Mr(calc)** | **Delta** | **Miss** | **Score** | **Expect** | **Peptide** |
| --- | --- | --- | --- | --- | --- | --- | --- |
| **392.2222** | **782.4298** | **782.4286** | **0.0012** | **0** | **44** | **0.12** | **R.GAAGLPGPK.G** |
| **426.2175** | **850.4204** | **850.4185** | **0.0020** | **0** | **42** | **0.26** | **R.GFSGLDGAK.G** |
| **426.7385** | **851.4624** | **851.4250** | **0.0375** | **0** | **41** | **0.38** | **R.GPAGPQGPR.G** |
| **449.7592** | **897.5038** | **897.5032** | **0.0006** | **0** | **56** | **0.0097** | **R.GVVGLPGQR.G** |
| **499.7854** | **997.5562** | **997.4465** | **0.1097** | **0** | **45** | **0.14** | **R.PGPPGPPGSR.G** |
| **529.7505** | **1057.4864** | **1057.4863** | **0.0002** | **0** | **61** | **0.0049** | **R.PGEPGLMGPR.G** |
| **553.7845** | **1105.5544** | **1105.5516** | **0.0028** | **0** | **58** | **0.011** | **R.GVQGPPGPAGPR.G** |
| **573.7948** | **1145.5750** | **1145.5751** | **-0.0000** | **0** | **42** | **0.44** | **R.GLPGTAGLPGMK.G** |
| **581.2907** | **1160.5668** | **1160.5648** | **0.0020** | **0** | **75** | **0.00022** | **R.GQAGVMGFPGPK.G** |
| **601.2966** | **1200.5786** | **1200.5775** | **0.0011** | **0** | **67** | **0.0015** | **R.GEPGNIGFPGPK.G** |
| **603.8127** | **1205.6108** | **1205.6081** | **0.0027** | **0** | **54** | **0.028** | **R.GFPGTPGLPGFK.G** |
| **653.8242** | **1305.6338** | **1305.6313** | **0.0025** | **0** | **73** | **0.00033** | [**R.GPSGPQGPSGAPGPK.G**](http://msct.smith.man.ac.uk/mascot/cgi/peptide_view.pl?file=../data/20120830/F291555847.dat&query=3333&hit=1&index=M00041&px=1&section=5&ave_thresh=51) |
| **664.8289** | **1327.6432** | **1327.6409** | **0.0024** | **0** | **66** | **0.0016** | **R.GFPGLPGPSGEPGK.Q** |
| **698.3663** | **1394.7180** | **1394.7163** | **0.0018** | **0** | **68** | **0.0011** | **K.GVGLGPGPMGLMGPR.G** |
| **730.3507** | **1458.6868** | **1458.6852** | **0.0017** | **0** | **116** | **1.8e-08** | **R.GSAGPPGATGFPGAAGR.V** |
| **733.3492** | **1464.6838** | **1464.6845** | **-0.0007** | **0** | **58** | **0.012** | **R.GEPGPTGLPGPPGER.G** |
| **739.3816** | **1476.7486** | **1476.7474** | **0.0013** | **0** | **64** | **0.0033** | **R.GLHGEFGLPGPAGPR.G** |
| **751.3564** | **1500.6982** | **1500.6958** | **0.0025** | **0** | **62** | **0.0051** | **R.GDGGPPGVTGFPGAAGR.T** |
| **754.8481** | **1507.6816** | **1507.6791** | **0.0026** | **0** | **52** | **0.045** | [**R.TGETGASGPPGFTGEK.G**](http://msct.smith.man.ac.uk/mascot/cgi/peptide_view.pl?file=../data/20120830/F291555847.dat&query=4785&hit=1&index=M00041&px=1&section=5&ave_thresh=51) |
| **755.8665** | **1509.7184** | **1509.7172** | **0.0012** | **0** | **48** | **0.11** | **R.GAPGAVGAPGPAGATGDR.G** |
| **761.8547** | **1521.6948** | **1521.6947** | **0.0001** | **0** | **43** | **0.41** | **R.PGEVGPPGPPGPAGEK.G** |
| **767.8856** | **1533.7566** | **1533.7536** | **0.0031** | **0** | **63** | **0.0043** | **R.GETGPAGPAGPVGPAGAR.G** |
| **781.8950** | **1561.7754** | **1561.7737** | **0.0018** | **0** | **60** | **0.0082** | **K.DGLNGLPGPIGPPGPR.G** |
| **781.9187** | **1561.8228** | **1561.8213** | **0.0016** | **0** | **72** | **0.00046** | **K.GAAGLPGVAGAPGLPGPR.G** |
| **788.9089** | **1575.8032** | **1575.8006** | **0.0027** | **0** | **86** | **2.1e-05** | **R.GEPGPAGSVGPVGAVGPR.G** |
| **789.9112** | **1577.8078** | **1577.8050** | **0.0029** | **0** | **51** | **0.067** | **R.GLTGPIGPPGPAGATGDK.G** |
| **790.3962** | **1578.7778** | **1578.7751** | **0.0028** | **0** | **74** | **0.00029** | **R.GPPGQSGAAGPTGPIGSR.G** |
| **793.3901** | **1584.7656** | **1584.7645** | **0.0012** | **0** | **63** | **0.0035** | **K.GANGAPGIAGAPGFPGAR.G** |
| **808.3954** | **1614.7762** | **1614.8114** | **-0.0352** | **0** | **81** | **5.9e-05** | **K.GELGPVGNPGPSGPAGPR.G** |
| **817.3947** | **1632.7748** | **1632.7744** | **0.0005** | **1** | **77** | **0.00015** | [**R.GFSGLDGAKGDSGPAGPK.G**](http://msct.smith.man.ac.uk/mascot/cgi/peptide_view.pl?file=../data/20120830/F291555847.dat&query=5691&hit=1&index=M00041&px=1&section=5&ave_thresh=51) |
| **853.8916** | **1705.7686** | **1705.7656** | **0.0031** | **0** | **93** | **3.9e-06** | **K.DGEAGAQGPPGPAGPAGER.G** |
| **872.8599** | **1743.7052** | **1743.7006** | **0.0046** | **0** | **64** | **0.003** | **K.GEPGSPGENGAPGQMGPR.G** |
| **881.9293** | **1761.8440** | **1761.8394** | **0.0046** | **0** | **88** | **1.5e-05** | **R.GPPGAVGNPGVNGAPGEAGR.D** |
| **883.9205** | **1765.8264** | **1765.8231** | **0.0033** | **0** | **62** | **0.0053** | **K.PGEQGVPGDLGAPGPSGAR.G** |
| **900.9391** | **1799.8636** | **1799.8625** | **0.0012** | **0** | **90** | **8.7e-06** | **R.GPPGPMGPPGLAGPPGESGR.E** |
| **909.4557** | **1816.8968** | **1816.8956** | **0.0013** | **0** | **55** | **0.031** | **R.TGPPGPSGITGPPGPPGAAGK.E** |
| **914.9468** | **1827.8790** | **1827.8752** | **0.0039** | **0** | **71** | **0.00068** | **R.VGPPGPSGNAGPPGPPGPAGK.E** |
| **925.9248** | **1849.8350** | **1849.8330** | **0.0020** | **0** | **65** | **0.003** | **K.GEPGPTGVQGPPGPAGEEGK.R** |
| **992.4754** | **1982.9362** | **1982.9707** | **-0.0344** | **1** | **53** | **0.044** | **R.QYDAKGVGLGPGPMGLMGPR.G** |
| **1003.4830** | **2004.9514** | **2004.9501** | **0.0013** | **1** | **62** | **0.0058** | **K.GEPGPTGVQGPPGPAGEEGKR.G** |
| **1017.5240** | **2033.0334** | **2033.0290** | **0.0044** | **1** | **43** | **0.46** | **K.EGLGGLPGIDGRPGPTGPAGAR.G** |
| **1021.5140** | **2041.0134** | **2041.0090** | **0.0045** | **1** | **71** | **0.00069** | **K.HGNRGEPGPAGSVGPVGAVGPR.G** |
| **1034.4930** | **2066.9714** | **2066.9658** | **0.0057** | **0** | **78** | **0.00014** | [**R.GEVGPAGPNGFAGPAGAAGQPGAK.G**](http://msct.smith.man.ac.uk/mascot/cgi/peptide_view.pl?file=../data/20120830/F291555847.dat&query=7547&hit=2&index=M00041&px=1&section=5&ave_thresh=51) |
| **1045.9990** | **2089.9834** | **2089.9778** | **0.0057** | **0** | **69** | **0.0013** | **K.GSPGADGPAGAPGTPGPQGIGGQR.G** |
| **1054.4890** | **2106.9634** | **2106.9607** | **0.0028** | **0** | **46** | **0.23** | **R.GEPGPAGPAGFAGPPGADGQPGAK.G** |
| **1073.0700** | **2144.1254** | **2144.1226** | **0.0028** | **0** | **114** | **3.5e-08** | **R.GLPGVAGSLGEPGPLGIAGPPGAR.G** |
| **1093.0310** | **2184.0474** | **2184.0448** | **0.0027** | **0** | **53** | **0.044** | **R.GETGPAGPPGAPGAPGAPGPVGPAGK.S** |
| **1100.4840** | **2198.9534** | **2198.9499** | **0.0036** | **0** | **73** | **0.00047** | **K.GDAGAPGAPGSQGAPGLQGMPGER.G** |
| **1135.5590** | **2269.1034** | **2269.0975** | **0.0059** | **0** | **65** | **0.0028** | **K.GDAGPAGPAGPTGAPGPIGNVGAPGPK.G** |
| **1195.6020** | **2389.1894** | **2389.1874** | **0.0020** | **0** | **51** | **0.076** | **R.GEVGLPGVSGPVGPPGNPGANGLAGAK.G** |
| **1206.0670** | **2410.1194** | **2410.1149** | **0.0045** | **1** | **77** | **0.00021** | **R.GEVGPAGPNGFAGPAGAAGQPGAKGER.G** |
| **1249.1090** | **2496.2034** | **2496.1994** | **0.0041** | **1** | **57** | **0.023** | **K.GDRGETGPAGPPGAPGAPGAPGPVGPAGK.S** |
| **1251.1230** | **2500.2314** | **2500.2194** | **0.0120** | **1** | **41** | **0.78** | **R.GPPGSAGTPGKDGLNGLPGPIGPPGPR.G** |
| **1282.6070** | **2563.1994** | **2563.1940** | **0.0055** | **0** | **99** | **1.3e-06** | **R.GNDGATGAAGPPGPTGPAGPPGFPGAVGAK.G** |
| **1287.6420** | **2573.2694** | **2573.2147** | **0.0547** | **0** | **83** | **5e-05** | **R.GSDGSVGPVGPAGPNGSAGPPGFPGAPGPK.G** |
| **1291.6180** | **2581.2214** | **2581.2157** | **0.0057** | **0** | **41** | **0.84** | **K.GENGPVGPTGPVGAAGPSGPNGPPGPAGSR.G** |
| **1353.6280** | **2705.2414** | **2705.2318** | **0.0097** | **0** | **90** | **1.1e-05** | **R.GFSGLQGPPGPPGSPGEQGPSGASGPAGPR.G** |
| **951.1220** | **2850.3442** | **2850.3421** | **0.0021** | **1** | **79** | **0.00014** | [**K.GEQGPAGPPGFQGLPGPAGTTGEVGKPGER.G**](http://msct.smith.man.ac.uk/mascot/cgi/peptide_view.pl?file=../data/20120830/F291555847.dat&query=9032&hit=1&index=M00041&px=1&section=5&ave_thresh=51) |
| **1429.2100** | **2856.4054** | **2856.4003** | **0.0052** | **1** | **65** | **0.0038** | [**R.GLTGPIGPPGPAGATGDKGESGPSGPAGPTGAR.G**](http://msct.smith.man.ac.uk/mascot/cgi/peptide_view.pl?file=../data/20120830/F291555847.dat&query=9045&hit=1&index=M00041&px=1&section=5&ave_thresh=51) |
| **1023.1640** | **3066.4702** | **3066.4643** | **0.0059** | **1** | **59** | **0.015** | **K.GEPGDAGAKGDAGPAGPAGPTGAPGPIGNVGAPGPK.G** |
| **1135.5310** | **3403.5712** | **3403.5666** | **0.0046** | **1** | **42** | **0.65** | **R.GNDGATGAAGPPGPTGPAGPPGFPGAVGAKGEAGPQGAR.G** |
