# Supplementary material for: A Molecular Phylogeny of Plesiorycteropus Reassigns the Extinct Mammalian Order ‘Bibymalagasia’
Source: PLoS One. 2013 Mar 26;8(3):e59614. doi: 10.1371/journal.pone.0059614 (PMC3608660; doi:10.1371/journal.pone.0059614)
Supplement: Table S6 — Mascot results for Chaetophractus bone acid-insoluble protein digest LC-MS data. (DOCX) [file pone.0059614.s009.docx]

Table S6 - Mascot search results of LC-MS data against local database showing observed, expected and calculated molecular weights, the difference between expected and calculated molecular weights (Delta), the number of missed cleavages, peptide ion score, Expect score and peptide sequence (where underline represents modified amino acid) for *Chaetophractus* bone acid-insoluble protein digest.

| **Observed** | **Mr(expt)** | **Mr(calc)** | **Delta** | **Miss** | **Score** | **Expect** | **Peptide** |
| --- | --- | --- | --- | --- | --- | --- | --- |
| **449.7590** | **897.5034** | **897.5032** | **0.0002** | **0** | **43** | **0.22** | [**R.GVVGLPGQR.G**](http://msct.smith.man.ac.uk/mascot/cgi/peptide_view.pl?file=../data/20120830/F291555826.dat&query=443&hit=1&index=M00050&px=1&section=5&ave_thresh=51) |
| **551.7792** | **1101.5438** | **1101.5455** | **-0.0016** | **0** | **64** | **0.0027** | [**R.GFPGADGIAGPK.G**](http://msct.smith.man.ac.uk/mascot/cgi/peptide_view.pl?file=../data/20120830/F291555826.dat&query=1110&hit=1&index=M00050&px=1&section=5&ave_thresh=51) |
| **553.7833** | **1105.5520** | **1105.5516** | **0.0004** | **0** | **46** | **0.15** | **R.GVQGPPGPAGPR.G** |
| **580.2943** | **1158.5740** | **1158.5669** | **0.0071** | **0** | **70** | **0.00066** | **R.GEAGNIGFPGPK.G** |
| **589.7793** | **1177.5440** | **1177.5438** | **0.0003** | **0** | **78** | **0.0001** | **R.GQAGVMGFPGPK.G** |
| **591.8094** | **1181.6042** | **1181.6041** | **0.0002** | **0** | **46** | **0.13** | [**K.EGPVGLPGIDGR.P**](http://msct.smith.man.ac.uk/mascot/cgi/peptide_view.pl?file=../data/20120830/F291555826.dat&query=1526&hit=1&index=M00050&px=1&section=5&ave_thresh=51) |
| **618.3110** | **1234.6074** | **1234.6054** | **0.0020** | **0** | **42** | **0.39** | **R.GEAGAAGPAGPAGPR.G** |
| **627.3289** | **1252.6432** | **1252.6412** | **0.0021** | **0** | **82** | **3.9e-05** | **R.GLPGSPGNIGPAGK.E** |
| **648.3554** | **1294.6962** | **1294.6994** | **-0.0031** | **0** | **97** | **1.3e-06** | [**R.GIPGPVGAVGATGAR.G**](http://msct.smith.man.ac.uk/mascot/cgi/peptide_view.pl?file=../data/20120830/F291555826.dat&query=1987&hit=1&index=M00050&px=1&section=5&ave_thresh=51) |
| **649.3084** | **1296.6022** | **1296.6058** | **-0.0036** | **0** | **66** | **0.0015** | **K.GESGPSGPAGPTGAR.G** |
| **653.8226** | **1305.6306** | **1305.6313** | **-0.0007** | **0** | **61** | **0.006** | **R.GPSGPQGPSGAPGPK.G** |
| **664.8281** | **1327.6416** | **1327.6409** | **0.0008** | **0** | **55** | **0.024** | **R.GFPGLPGPSGEPGK.Q** |
| **714.3608** | **1426.7070** | **1426.7061** | **0.0009** | **0** | **41** | **0.61** | **K.GVGLGPGPMGLMGPR.G** |
| **716.8391** | **1431.6636** | **1431.6630** | **0.0006** | **0** | **41** | **0.6** | **R.TGETGAGGPPGFAGEK.G** |
| **730.3512** | **1458.6878** | **1458.6852** | **0.0027** | **0** | **75** | **0.00023** | **R.GSAGPPGATGFPGAAGR.V** |
| **733.3497** | **1464.6848** | **1464.6845** | **0.0003** | **0** | **53** | **0.034** | [**R.GEPGPTGLPGPPGER.G**](http://msct.smith.man.ac.uk/mascot/cgi/peptide_view.pl?file=../data/20120830/F291555826.dat&query=2836&hit=1&index=M00050&px=1&section=5&ave_thresh=51) |
| **739.3818** | **1476.7490** | **1476.7474** | **0.0017** | **0** | **62** | **0.0052** | **R.GLHGEFGLPGPAGPR.G** |
| **751.3560** | **1500.6974** | **1500.6958** | **0.0017** | **0** | **49** | **0.089** | [**R.GDGGPPGVTGFPGAAGR.T**](http://msct.smith.man.ac.uk/mascot/cgi/peptide_view.pl?file=../data/20120830/F291555826.dat&query=3052&hit=1&index=M00050&px=1&section=5&ave_thresh=51) |
| **753.8688** | **1505.7230** | **1505.7223** | **0.0008** | **0** | **53** | **0.037** | **R.GETGPSGPAGPAGPAGAR.G** |
| **781.8951** | **1561.7756** | **1561.7737** | **0.0020** | **0** | **51** | **0.067** | **K.DGLNGLPGPIGPPGPR.G** |
| **781.9191** | **1561.8236** | **1561.8213** | **0.0024** | **0** | **58** | **0.014** | [**K.GAAGLPGVAGAPGLPGPR.G**](http://msct.smith.man.ac.uk/mascot/cgi/peptide_view.pl?file=../data/20120830/F291555826.dat&query=3341&hit=1&index=M00050&px=1&section=5&ave_thresh=51) |
| **787.9136** | **1573.8126** | **1573.8100** | **0.0026** | **0** | **53** | **0.036** | **R.GLTGPIGPPGPAGAPGDK.G** |
| **793.8810** | **1585.7474** | **1585.7485** | **-0.0010** | **0** | **70** | **0.00083** | [**K.GANGAPGIAGAPGFPGAR.G**](http://msct.smith.man.ac.uk/mascot/cgi/peptide_view.pl?file=../data/20120830/F291555826.dat&query=3446&hit=1&index=M00050&px=1&section=5&ave_thresh=51) |
| **802.9263** | **1603.8380** | **1603.8319** | **0.0062** | **0** | **90** | **8.4e-06** | [**R.GEPGPVGSVGPVGAVGPR.G**](http://msct.smith.man.ac.uk/mascot/cgi/peptide_view.pl?file=../data/20120830/F291555826.dat&query=3520&hit=1&index=M00050&px=1&section=5&ave_thresh=51) |
| **816.4139** | **1630.8132** | **1630.8064** | **0.0069** | **0** | **74** | **0.00034** | [**K.GELGPVGNPGPAGPAGPR.G**](http://msct.smith.man.ac.uk/mascot/cgi/peptide_view.pl?file=../data/20120830/F291555826.dat&query=3669&hit=3&index=M00050&px=1&section=5&ave_thresh=51) |
| **840.8803** | **1679.7460** | **1679.7499** | **-0.0039** | **0** | **77** | **0.00016** | **K.DGEAGAQGAPGPAGPAGER.G** |
| **883.9204** | **1765.8262** | **1765.8231** | **0.0031** | **0** | **67** | **0.0018** | **K.PGEQGVPGDLGAPGPSGAR.G** |
| **908.9362** | **1815.8578** | **1815.8574** | **0.0004** | **0** | **72** | **0.00052** | **R.GPPGPMGPPGLAGPPGESGR.E** |
| **909.4560** | **1816.8974** | **1816.8956** | **0.0019** | **0** | **56** | **0.023** | **R.TGPPGPSGITGPPGPPGAAGK.E** |
| **928.9614** | **1855.9082** | **1855.9065** | **0.0018** | **0** | **40** | **0.87** | **R.VGPPGPSGNAGPPGPPGPVGK.E** |
| **932.4414** | **1862.8682** | **1862.8647** | **0.0036** | **0** | **52** | **0.057** | [**K.GEPGPTGIQGPPGPAGEEGK.R**](http://msct.smith.man.ac.uk/mascot/cgi/peptide_view.pl?file=../data/20120830/F291555826.dat&query=4539&hit=1&index=M00050&px=1&section=5&ave_thresh=51) |
| **961.4601** | **1920.9056** | **1920.9038** | **0.0018** | **1** | **62** | **0.0057** | **K.SGDRGETGPSGPAGPAGPAGAR.G** |
| **1013.4870** | **2024.9594** | **2024.9552** | **0.0043** | **0** | **85** | **3.1e-05** | **R.GEVGPAGANGFAGPAGAAGQPGAK.G** |
| **1048.0250** | **2094.0354** | **2093.9978** | **0.0376** | **0** | **43** | **0.47** | **K.GEPGVVGAPGTAGPSGSGGLPGER.G** |
| **702.3394** | **2103.9964** | **2103.9934** | **0.0030** | **0** | **40** | **0.89** | **K.GSPGADGPAGAPGTPGPQGIAGQR.G** |
| **1066.0620** | **2130.1094** | **2130.1070** | **0.0025** | **0** | **97** | **2e-06** | **R.GLPGVAGAVGEPGPLGISGPPGAR.G** |
| **717.3317** | **2148.9733** | **2148.9713** | **0.0020** | **0** | **47** | **0.19** | **R.GEPGPPGPAGFAGPPGADGQPGAK.G** |
| **738.9944** | **2213.9614** | **2213.9608** | **0.0006** | **0** | **42** | **0.69** | **K.GDAGAPGAPGSQGAPGLQGMPGER.G** |
| **829.0823** | **2484.2251** | **2484.2245** | **0.0006** | **1** | **48** | **0.16** | **R.GPPGSAGTPGKDGLNGLPGPIGPPGPR.G** |
| **833.0746** | **2496.2020** | **2496.1994** | **0.0026** | **1** | **54** | **0.04** | **K.GDRGETGPAGPPGAPGAPGAPGPVGPAGK.S** |
| **850.0760** | **2547.2062** | **2547.1991** | **0.0071** | **0** | **55** | **0.037** | [**R.GNDGATGAAGPPGPTGPAGPPGFPGAVGAK.G**](http://msct.smith.man.ac.uk/mascot/cgi/peptide_view.pl?file=../data/20120830/F291555826.dat&query=5396&hit=1&index=M00050&px=1&section=5&ave_thresh=51) |
| **888.4211** | **2662.2415** | **2662.2372** | **0.0043** | **0** | **52** | **0.063** | **R.GFSGLQGPPGAPGSPGEQGPSGASGPAGPR.G** |
| **950.7947** | **2849.3623** | **2849.3581** | **0.0042** | **1** | **74** | **0.00043** | **K.GEQGPAGPPGFQGLPGPAGTTGEVGKPGER.G** |
