# Supplementary material for: A Molecular Phylogeny of Plesiorycteropus Reassigns the Extinct Mammalian Order ‘Bibymalagasia’
Source: PLoS One. 2013 Mar 26;8(3):e59614. doi: 10.1371/journal.pone.0059614 (PMC3608660; doi:10.1371/journal.pone.0059614)
Supplement: Table S7 — Mascot results for Plesiorycteropus bone acid-insoluble protein digest LC-MS data. (DOCX) [file pone.0059614.s010.docx]

Table S7 – Mascot search results of LC-MS data against local database showing observed, expected and calculated molecular weights, the difference between expected and calculated molecular weights (Delta), the number of missed cleavages, peptide ion score, Expect score and peptide sequence (where underline represents modified amino acid) for *Plesiorycteropus* bone acid-insoluble protein digest.

| **Observed** | **Mr(expt)** | **Mr(calc)** | **Delta** | **Miss** | **Score** | **Expect** | **Peptide** |
| --- | --- | --- | --- | --- | --- | --- | --- |
| **420.7380** | **839.4614** | **839.4613** | **0.0001** | **0** | **42** | **0.21** | **K.GNVGLAGPR.G** |
| **449.7589** | **897.5032** | **897.5032** | **0.0000** | **0** | **62** | **0.0026** | **R.GVVGLPGQR.G** |
| **469.2587** | **936.5028** | **936.4665** | **0.0363** | **0** | **41** | **0.38** | **R.GPAGPSGPPGK.D** |
| **499.7852** | **997.5558** | **997.4465** | **0.1093** | **0** | **54** | **0.02** | **R.PGPPGPPGSR.G** |
| **529.7504** | **1057.4862** | **1057.4863** | **-0.0000** | **0** | **62** | **0.004** | **R.PGEPGLMGPR.G** |
| **551.7794** | **1101.5442** | **1101.5455** | **-0.0012** | **0** | **58** | **0.011** | **R.GFPGADGIAGPK.G** |
| **553.7831** | **1105.5516** | **1105.5516** | **0.0000** | **0** | **46** | **0.14** | **R.GVQGPPGPAGPR.G** |
| **578.8012** | **1155.5878** | **1155.5884** | **-0.0006** | **0** | **47** | **0.11** | [**K.EGLGGLPGIDGR.P**](http://msct.smith.man.ac.uk/mascot/cgi/peptide_view.pl?file=../data/20120830/F291555825.dat&query=1206&hit=1&index=M00044&px=1&section=5&ave_thresh=52) |
| **589.2868** | **1176.5590** | **1176.5598** | **-0.0007** | **0** | **69** | **0.00084** | [**R.GQAGVMGFPGPK.G**](http://msct.smith.man.ac.uk/mascot/cgi/peptide_view.pl?file=../data/20120830/F291555825.dat&query=1311&hit=1&index=M00044&px=1&section=5&ave_thresh=52) |
| **601.7894** | **1201.5642** | **1201.5615** | **0.0027** | **0** | **57** | **0.013** | [**R.GEPGNIGFPGPK.G**](http://msct.smith.man.ac.uk/mascot/cgi/peptide_view.pl?file=../data/20120830/F291555825.dat&query=1443&hit=1&index=M00044&px=1&section=5&ave_thresh=52) |
| **618.2934** | **1234.5722** | **1234.6054** | **-0.0332** | **0** | **45** | **0.22** | **R.GEAGAAGPAGPAGPR.G** |
| **619.3362** | **1236.6578** | **1236.6575** | **0.0004** | **0** | **82** | **3.8e-05** | **R.GIPGPVGAAGAAGAR.G** |
| **619.8058** | **1237.5970** | **1237.5979** | **-0.0009** | **0** | **53** | **0.03** | [**R.GFPGTPGLPGFK.G**](http://msct.smith.man.ac.uk/mascot/cgi/peptide_view.pl?file=../data/20120830/F291555825.dat&query=1643&hit=1&index=M00044&px=1&section=5&ave_thresh=52) |
| **620.3201** | **1238.6256** | **1238.6255** | **0.0001** | **0** | **53** | **0.032** | **R.GLPGSPGNVGPAGK.E** |
| **649.3106** | **1296.6066** | **1296.6058** | **0.0008** | **0** | **49** | **0.069** | **K.GESGPSGPAGPTGAR.G** |
| **653.8243** | **1305.6340** | **1305.6313** | **0.0027** | **0** | **72** | **0.00043** | **R.GPSGPQGPSGAPGPK.G** |
| **664.8278** | **1327.6410** | **1327.6409** | **0.0002** | **0** | **62** | **0.004** | **R.GFPGLPGPSGEPGK.Q** |
| **714.3602** | **1426.7058** | **1426.7061** | **-0.0003** | **0** | **59** | **0.0082** | **K.GVGLGPGPMGLMGPR.G** |
| **730.3496** | **1458.6846** | **1458.6852** | **-0.0005** | **0** | **74** | **0.00032** | **R.GSAGPPGATGFPGAAGR.V** |
| **733.3484** | **1464.6822** | **1464.6845** | **-0.0023** | **0** | **42** | **0.42** | **R.GEPGPTGLPGPPGER.G** |
| **739.3804** | **1476.7462** | **1476.7474** | **-0.0011** | **0** | **57** | **0.016** | [**R.GLHGEFGLPGPAGPR.G**](http://msct.smith.man.ac.uk/mascot/cgi/peptide_view.pl?file=../data/20120830/F291555825.dat&query=3016&hit=1&index=M00044&px=1&section=5&ave_thresh=52) |
| **739.8585** | **1477.7024** | **1477.6685** | **0.0339** | **0** | **56** | **0.021** | **R.PGEAGPPGPPGPAGEK.G** |
| **754.8471** | **1507.6796** | **1507.6791** | **0.0006** | **0** | **43** | **0.34** | [**R.TGETGASGPPGFTGEK.G**](http://msct.smith.man.ac.uk/mascot/cgi/peptide_view.pl?file=../data/20120830/F291555825.dat&query=3238&hit=1&index=M00044&px=1&section=5&ave_thresh=52) |
| **775.3384** | **1548.6622** | **1548.6627** | **-0.0005** | **0** | **69** | **0.00098** | [**R.GDGGPPGMTGFPGAAGR.T**](http://msct.smith.man.ac.uk/mascot/cgi/peptide_view.pl?file=../data/20120830/F291555825.dat&query=3554&hit=2&index=M00044&px=1&section=5&ave_thresh=52) |
| **775.3868** | **1548.7590** | **1548.7685** | **-0.0095** | **1** | **65** | **0.0028** | **R.GFPGTPGLPGFKGNR.G** |
| **780.9105** | **1559.8064** | **1559.8056** | **0.0008** | **0** | **66** | **0.0019** | **R.GETGPAGPAGPIGPVGAR.G** |
| **781.8939** | **1561.7732** | **1561.7737** | **-0.0004** | **0** | **74** | **0.00031** | **K.DGLNGLPGPIGPPGPR.G** |
| **785.8766** | **1569.7386** | **1569.7383** | **0.0003** | **0** | **97** | **1.5e-06** | [**R.GPPGQSGAAGPTGSIGSR.G**](http://msct.smith.man.ac.uk/mascot/cgi/peptide_view.pl?file=../data/20120830/F291555825.dat&query=3738&hit=1&index=M00044&px=1&section=5&ave_thresh=52) |
| **788.9081** | **1575.8016** | **1575.8006** | **0.0011** | **0** | **81** | **6.7e-05** | **R.GEPGPAGSVGPVGAVGPR.G** |
| **793.8818** | **1585.7490** | **1585.7485** | **0.0006** | **0** | **63** | **0.0041** | **K.GANGAPGIAGAPGFPGAR.G** |
| **796.9234** | **1591.8322** | **1591.8318** | **0.0004** | **0** | **50** | **0.079** | [**K.GATGLPGVAGAPGLPGPR.G**](http://msct.smith.man.ac.uk/mascot/cgi/peptide_view.pl?file=../data/20120830/F291555825.dat&query=3950&hit=1&index=M00044&px=1&section=5&ave_thresh=52) |
| **803.9069** | **1605.7992** | **1605.7999** | **-0.0006** | **0** | **49** | **0.11** | **R.GLTGPIGPPGPAGAPGDK.G** |
| **811.4022** | **1620.7898** | **1620.7856** | **0.0042** | **0** | **62** | **0.0048** | [**R.GPNGEAGSAGPVGPPGLR.G**](http://msct.smith.man.ac.uk/mascot/cgi/peptide_view.pl?file=../data/20120830/F291555825.dat&query=4172&hit=1&index=M00044&px=1&section=5&ave_thresh=52) |
| **815.4059** | **1628.7972** | **1628.7907** | **0.0065** | **1** | **70** | **0.00076** | **R.GGPGSRGFPGADGIAGPK.G** |
| **816.3925** | **1630.7704** | **1630.8064** | **-0.0359** | **0** | **73** | **0.00038** | **K.GELGPVGNPGPSGPAGPR.G** |
| **841.3739** | **1680.7332** | **1680.7339** | **-0.0007** | **0** | **67** | **0.0018** | [**K.DGEAGAQGAPGPAGPAGER.G**](http://msct.smith.man.ac.uk/mascot/cgi/peptide_view.pl?file=../data/20120830/F291555825.dat&query=4503&hit=1&index=M00044&px=1&section=5&ave_thresh=52) |
| **889.9429** | **1777.8712** | **1777.8707** | **0.0005** | **1** | **48** | **0.14** | **K.RGPNGEAGSAGPVGPPGLR.G** |
| **896.4468** | **1790.8790** | **1790.8799** | **-0.0009** | **0** | **95** | **2.8e-06** | **R.TGPSGPSGITGPPGPPGAAGK.E** |
| **908.9362** | **1815.8578** | **1815.8574** | **0.0004** | **0** | **94** | **3.6e-06** | **R.GPPGPMGPPGLAGPPGESGR.E** |
| **912.9050** | **1823.7954** | **1823.7922** | **0.0032** | **0** | **59** | **0.0099** | [**R.GPPGNVGNPGVNGAPGEAGR.D**](http://msct.smith.man.ac.uk/mascot/cgi/peptide_view.pl?file=../data/20120830/F291555825.dat&query=5250&hit=1&index=M00044&px=1&section=5&ave_thresh=52) |
| **915.4384** | **1828.8622** | **1828.8592** | **0.0031** | **0** | **45** | **0.26** | **R.VGPPGPSGNAGPPGPPGPAGK.E** |
| **988.5017** | **1974.9888** | **1974.9872** | **0.0017** | **1** | **72** | **0.00062** | **K.SGDRGETGPAGPAGPIGPVGAR.G** |
| **1003.4840** | **2004.9534** | **2004.9501** | **0.0033** | **1** | **57** | **0.019** | **K.GEPGPTGVQGPPGPAGEEGKR.G** |
| **1021.5120** | **2041.0094** | **2041.0090** | **0.0005** | **1** | **48** | **0.15** | **K.HGNRGEPGPAGSVGPVGAVGPR.G** |
| **1034.9820** | **2067.9494** | **2067.9498** | **-0.0003** | **0** | **69** | **0.0012** | **R.GEVGPAGPNGFAGPAGAAGQPGAK.G** |
| **1054.4870** | **2106.9594** | **2106.9567** | **0.0028** | **0** | **52** | **0.058** | **K.GSPGADGPAGAPGTPGPQGIGGQR.G** |
| **1065.0720** | **2128.1294** | **2128.1277** | **0.0018** | **0** | **98** | **1.4e-06** | **R.GLPGVAGALGEPGPLGIAGPPGAR.G** |
| **1075.9870** | **2149.9594** | **2149.9553** | **0.0042** | **0** | **42** | **0.66** | **R.GEPGPPGPAGFAGPPGADGQPGAK.G** |
| **1108.4800** | **2214.9454** | **2214.9448** | **0.0007** | **0** | **72** | **0.00057** | **K.GDAGAPGAPGSQGAPGLQGMPGER.G** |
| **1117.0360** | **2232.0574** | **2232.0560** | **0.0015** | **0** | **109** | **1.3e-07** | **R.GYPGNAGPVGAAGAPGPHGSVGPAGK.H** |
| **1148.0760** | **2294.1374** | **2294.1292** | **0.0083** | **0** | **83** | **5.7e-05** | **K.GDAGPPGPAGPTGAPGPIGNVGAPGPK.G** |
| **1203.1030** | **2404.1914** | **2404.1871** | **0.0044** | **0** | **59** | **0.013** | **R.GELGLPGVSGPVGPPGNPGANGLAGAK.G** |
| **1206.0730** | **2410.1314** | **2410.1149** | **0.0165** | **1** | **57** | **0.02** | **R.GEVGPAGPNGFAGPAGAAGQPGAKGER.G** |
| **824.4385** | **2470.2937** | **2470.2928** | **0.0008** | **1** | **63** | **0.0058** | **R.GERGLPGVAGALGEPGPLGIAGPPGAR.G** |
| **829.7398** | **2486.1976** | **2486.2038** | **-0.0062** | **1** | **42** | **0.64** | **R.GPPGSAGSPGKDGLNGLPGPIGPPGPR.G** |
| **1275.1010** | **2548.1874** | **2548.1831** | **0.0044** | **0** | **55** | **0.037** | **R.GNDGATGAAGPPGPTGPAGPPGFPGAVGAK.G** |
| **1333.1110** | **2664.2074** | **2664.2052** | **0.0022** | **0** | **63** | **0.0051** | **R.GFSGLQGPPGAPGSPGEQGPSGASGPAGPR.G** |
| **939.7875** | **2816.3407** | **2816.3366** | **0.0041** | **1** | **53** | **0.058** | **K.GEQGPAGPPGFQGLPGPAGPAGEVGKPGER.G** |
| **970.4774** | **2908.4104** | **2908.4064** | **0.0040** | **1** | **70** | **0.001** | **K.GPKGENGPVGPTGPIGSAGPSGPNGPPGPAGSR.G** |
| **1071.5110** | **3211.5112** | **3211.5018** | **0.0093** | **1** | **41** | **0.98** | **R.GPSGPPGPDGNKGEPGVVGAPGTAGPSGPSGLPGER.G** |
