# Supplementary material for: A Molecular Phylogeny of Plesiorycteropus Reassigns the Extinct Mammalian Order ‘Bibymalagasia’
Source: PLoS One. 2013 Mar 26;8(3):e59614. doi: 10.1371/journal.pone.0059614 (PMC3608660; doi:10.1371/journal.pone.0059614)
Supplement: Table S8 — Mascot results for Setifer bone acid-insoluble protein digest LC-MS data. (DOCX) [file pone.0059614.s011.docx]

Table S8 – Mascot search results of LC-MS data against local database showing observed, expected and calculated molecular weights, the difference between expected and calculated molecular weights (Delta), the number of missed cleavages, peptide ion score, Expect score and peptide sequence (where underline represents modified amino acid) for *Setifer* bone acid-insoluble protein digest.

| **Observed** | **Mr(expt)** | **Mr(calc)** | **Delta** | **Miss** | **Score** | **Expect** | **Peptide** |
| --- | --- | --- | --- | --- | --- | --- | --- |
| **442.2352** | **882.4558** | **882.4196** | **0.0363** | **0** | **49** | **0.041** | [**R.GPTGPQGPR.G**](http://msct.smith.man.ac.uk/mascot/cgi/peptide_view.pl?file=../data/20120803/F291554706.dat&query=347&hit=1&index=M00040&px=1&section=5&ave_thresh=52) |
| **449.7587** | **897.5028** | **897.5032** | **-0.0004** | **0** | **43** | **0.2** | [**R.GVVGLPGQR.G**](http://msct.smith.man.ac.uk/mascot/cgi/peptide_view.pl?file=../data/20120803/F291554706.dat&query=384&hit=1&index=M00040&px=1&section=5&ave_thresh=52) |
| **529.7506** | **1057.4866** | **1057.4863** | **0.0004** | **0** | **63** | **0.0028** | **R.PGEPGLMGPR.G** |
| **550.7827** | **1099.5508** | **1099.5510** | **-0.0001** | **0** | **51** | **0.044** | **R.GLVGEPGPAGSK.G** |
| **553.7832** | **1105.5518** | **1105.5516** | **0.0002** | **0** | **54** | **0.027** | **R.GVQGPPGPAGPR.G** |
| **578.8015** | **1155.5884** | **1155.5884** | **0.0000** | **0** | **42** | **0.36** | **K.EGLGGLPGIDGR.P** |
| **589.7791** | **1177.5436** | **1177.5438** | **-0.0001** | **0** | **68** | **0.00098** | **R.GQAGVMGFPGPK.G** |
| **601.2961** | **1200.5776** | **1200.5775** | **0.0001** | **0** | **57** | **0.012** | [**R.GEPGNIGFPGPK.G**](http://msct.smith.man.ac.uk/mascot/cgi/peptide_view.pl?file=../data/20120803/F291554706.dat&query=1663&hit=1&index=M00040&px=1&section=5&ave_thresh=52) |
| **620.3203** | **1238.6260** | **1238.6255** | **0.0005** | **0** | **59** | **0.0081** | **R.GLPGSPGNVGPAGK.E** |
| **621.8018** | **1241.5890** | **1241.5888** | **0.0002** | **0** | **52** | **0.039** | **K.GLTGSPGSPGPDGK.T** |
| **628.3231** | **1254.6316** | **1254.6317** | **-0.0000** | **0** | **57** | **0.014** | [**R.GIPGPAGSAGATGAR.G**](http://msct.smith.man.ac.uk/mascot/cgi/peptide_view.pl?file=../data/20120803/F291554706.dat&query=1868&hit=1&index=M00040&px=1&section=5&ave_thresh=52) |
| **664.8279** | **1327.6412** | **1327.6409** | **0.0004** | **0** | **82** | **4.4e-05** | **R.GFPGLPGPSGEPGK.Q** |
| **726.3422** | **1450.6698** | **1450.6689** | **0.0010** | **0** | **73** | **0.00033** | **R.GEPGPSGLPGPPGER.G** |
| **730.3502** | **1458.6858** | **1458.6852** | **0.0007** | **0** | **87** | **1.6e-05** | **R.GSAGPPGATGFPGAAGR.V** |
| **739.3681** | **1476.7216** | **1476.7474** | **-0.0257** | **0** | **52** | **0.048** | **R.GLHGEFGLPGPAGPR.G** |
| **753.8574** | **1505.7002** | **1505.6998** | **0.0004** | **0** | **64** | **0.0029** | [**R.PGEVGPPGPPGPAGEK.G**](http://msct.smith.man.ac.uk/mascot/cgi/peptide_view.pl?file=../data/20120803/F291554706.dat&query=3039&hit=2&index=M00040&px=1&section=5&ave_thresh=52) |
| **756.8450** | **1511.6754** | **1511.6740** | **0.0014** | **0** | **46** | **0.19** | **R.TGETGASGPTGFTGEK.G** |
| **775.3389** | **1548.6632** | **1548.6627** | **0.0005** | **0** | **63** | **0.0038** | [**R.GDGGPPGMTGFPGAAGR.T**](http://msct.smith.man.ac.uk/mascot/cgi/peptide_view.pl?file=../data/20120803/F291554706.dat&query=3264&hit=2&index=M00040&px=1&section=5&ave_thresh=52) |
| **780.9105** | **1559.8064** | **1559.8056** | **0.0008** | **0** | **68** | **0.0011** | **R.GETGPAGPAGPIGPVGAR.G** |
| **781.9183** | **1561.8220** | **1561.8213** | **0.0008** | **0** | **51** | **0.06** | **K.GAAGLPGVAGAPGLPGPR.G** |
| **785.3851** | **1568.7556** | **1568.7543** | **0.0013** | **0** | **99** | **1.1e-06** | **R.GPPGQSGAAGPTGSIGSR.G** |
| **793.8821** | **1585.7496** | **1585.7485** | **0.0012** | **0** | **65** | **0.0026** | **K.GANGAPGIAGAPGFPGAR.G** |
| **803.9080** | **1605.8014** | **1605.7999** | **0.0016** | **0** | **72** | **0.00046** | **R.GLTGPIGPPGPAGSPGDK.G** |
| **816.4110** | **1630.8074** | **1630.8064** | **0.0011** | **0** | **94** | **3.4e-06** | [**K.GELGPVGNPGPSGPAGPR.G**](http://msct.smith.man.ac.uk/mascot/cgi/peptide_view.pl?file=../data/20120803/F291554706.dat&query=3670&hit=2&index=M00040&px=1&section=5&ave_thresh=52) |
| **823.4056** | **1644.7966** | **1644.7955** | **0.0011** | **1** | **69** | **0.001** | **R.GLVGEPGPAGSKGESGSK.G** |
| **861.8880** | **1721.7614** | **1721.7605** | **0.0009** | **0** | **74** | **0.00035** | **K.DGEAGAQGPPGPAGPAGER.G** |
| **866.9224** | **1731.8302** | **1731.8653** | **-0.0350** | **1** | **44** | **0.31** | **K.RGSPGEPGSAGPAGPPGLR.G** |
| **883.9191** | **1765.8236** | **1765.8231** | **0.0005** | **0** | **51** | **0.07** | **K.PGEQGVPGDLGAPGPSGAR.G** |
| **908.9370** | **1815.8594** | **1815.8574** | **0.0020** | **0** | **88** | **1.5e-05** | [**R.GPPGPMGPPGLAGPPGESGR.E**](http://msct.smith.man.ac.uk/mascot/cgi/peptide_view.pl?file=../data/20120803/F291554706.dat&query=4411&hit=2&index=M00040&px=1&section=5&ave_thresh=52) |
| **910.4277** | **1818.8408** | **1818.8384** | **0.0024** | **0** | **65** | **0.0029** | [**K.GEPGPAGVQGPPGPAGEEGK.R**](http://msct.smith.man.ac.uk/mascot/cgi/peptide_view.pl?file=../data/20120803/F291554706.dat&query=4431&hit=1&index=M00040&px=1&section=5&ave_thresh=52) |
| **914.9454** | **1827.8762** | **1827.8752** | **0.0011** | **0** | **57** | **0.016** | [**R.VGPPGPSGNAGPPGPPGPAGK.E**](http://msct.smith.man.ac.uk/mascot/cgi/peptide_view.pl?file=../data/20120803/F291554706.dat&query=4467&hit=1&index=M00040&px=1&section=5&ave_thresh=52) |
| **917.4327** | **1832.8508** | **1832.8905** | **-0.0397** | **0** | **81** | **7.4e-05** | **R.TGPPGPSGITGPPGPPGAAGK.E** |
| **988.9690** | **1975.9234** | **1975.9236** | **-0.0001** | **1** | **53** | **0.051** | **K.GEPGPAGVQGPPGPAGEEGKR.G** |
| **1048.0080** | **2094.0014** | **2093.9978** | **0.0036** | **0** | **70** | **0.00089** | [**K.GEPGVVGAPGTAGASGPGGLPGER.G**](http://msct.smith.man.ac.uk/mascot/cgi/peptide_view.pl?file=../data/20120803/F291554706.dat&query=5442&hit=1&index=M00040&px=1&section=5&ave_thresh=52) |
| **1054.9790** | **2107.9434** | **2107.9447** | **-0.0013** | **0** | **74** | **0.0004** | **R.GEPGPAGPAGFAGPPGADGQPGAK.G** |
| **1081.0660** | **2160.1174** | **2160.1175** | **-0.0001** | **0** | **84** | **3.9e-05** | [**R.GLPGVAGSLGEPGPLGISGPPGAR.G**](http://msct.smith.man.ac.uk/mascot/cgi/peptide_view.pl?file=../data/20120803/F291554706.dat&query=5675&hit=1&index=M00040&px=1&section=5&ave_thresh=52) |
| **1085.0330** | **2168.0514** | **2168.0498** | **0.0016** | **0** | **75** | **0.00031** | [**R.GETGPAGPPGAPGAPGAPGPVGPAGK.S**](http://msct.smith.man.ac.uk/mascot/cgi/peptide_view.pl?file=../data/20120803/F291554706.dat&query=5698&hit=1&index=M00040&px=1&section=5&ave_thresh=52) |
| **1108.9720** | **2215.9294** | **2215.9288** | **0.0007** | **0** | **78** | **0.00015** | **K.GDAGAPGAPGSQGAPGLQGMPGER.G** |
| **759.3614** | **2275.0624** | **2275.0618** | **0.0006** | **0** | **61** | **0.0086** | **R.GYPGNAGPVGNAGAPGPHGSVGPAGK.H** |
| **1148.0730** | **2294.1314** | **2294.1292** | **0.0023** | **0** | **90** | **1.1e-05** | **K.GDAGPPGPAGPTGAPGPIGNVGAPGPK.G** |
| **798.7209** | **2393.1409** | **2393.1360** | **0.0049** | **1** | **40** | **0.93** | **R.GEVGPAGPNGFAGPAGAAGQPGAKGER.G** |
| **1282.6060** | **2563.1974** | **2563.1940** | **0.0035** | **0** | **74** | **0.00043** | [**R.GNDGATGAAGPPGPTGPAGPPGFPGAVGAK.G**](http://msct.smith.man.ac.uk/mascot/cgi/peptide_view.pl?file=../data/20120803/F291554706.dat&query=6538&hit=1&index=M00040&px=1&section=5&ave_thresh=52) |
| **892.0900** | **2673.2482** | **2673.2420** | **0.0062** | **0** | **53** | **0.049** | **R.GFSGLQGPPGPPGSPGEQGPSGASGPAGPR.G** |
| **909.7787** | **2726.3143** | **2726.3121** | **0.0022** | **1** | **55** | **0.03** | **R.GAPGAVGAPGPAGATGDRGEAGAAGPAGPAGPR.G** |
| **951.1219** | **2850.3439** | **2850.3421** | **0.0018** | **1** | **55** | **0.031** | **K.GEQGPAGPPGFQGLPGPAGTTGEVGKPGER.G** |
| **957.1396** | **2868.3970** | **2868.4003** | **-0.0033** | **1** | **41** | **0.96** | **R.GLTGPIGPPGPAGSPGDKGESGPSGPAGPTGAR.G** |
| **962.4612** | **2884.3618** | **2884.3588** | **0.0030** | **1** | **60** | **0.012** | **K.GPKGENGAVGPTGPIGSAGPSGPNGPPGPAGSR.G** |
| **1145.5430** | **3433.6072** | **3433.5771** | **0.0300** | **1** | **47** | **0.24** | **R.GNDGATGAAGPPGPTGPAGPPGFPGAVGAKGEAGPQGTR.G** |
