# Supplementary material for: A Molecular Phylogeny of Plesiorycteropus Reassigns the Extinct Mammalian Order ‘Bibymalagasia’
Source: PLoS One. 2013 Mar 26;8(3):e59614. doi: 10.1371/journal.pone.0059614 (PMC3608660; doi:10.1371/journal.pone.0059614)
Supplement: Table S9 — Mascot results for Procavia bone acid-insoluble protein digest LC-MS data. (DOCX) [file pone.0059614.s012.docx]

Table S9 – Mascot search results of LC-MS data against local database showing observed, expected and calculated molecular weights, the difference between expected and calculated molecular weights (Delta), the number of missed cleavages, peptide ion score, Expect score and peptide sequence (where underline represents modified amino acid) for *Procavia* bone acid-insoluble protein digest.

| **Observed** | **Mr(expt)** | **Mr(calc)** | **Delta** | **Miss** | **Score** | **Expect** | **Peptide** |
| --- | --- | --- | --- | --- | --- | --- | --- |
| **392.2216** | **782.4286** | **782.4286** | **-0.0000** | **0** | **42** | **0.19** | **R.GAAGLPGPK.G** |
| **426.2170** | **850.4194** | **850.4185** | **0.0010** | **0** | **41** | **0.38** | **R.GFSGLDGAK.G** |
| **437.2263** | **872.4380** | **872.4352** | **0.0029** | **0** | **43** | **0.31** | **R.GPSGTQGLR.G** |
| **449.7589** | **897.5032** | **897.5032** | **0.0000** | **0** | **52** | **0.025** | [**R.GVVGLPGQR.G**](http://msct.smith.man.ac.uk/mascot/cgi/peptide_view.pl?file=../data/20120830/F291555856.dat&query=239&hit=1&index=M00043&px=1&section=5&ave_thresh=52) |
| **464.7406** | **927.4666** | **927.4662** | **0.0005** | **0** | **41** | **0.39** | **R.PGEAGLPGAK.G** |
| **528.2889** | **1054.5632** | **1054.5632** | **0.0001** | **1** | **62** | **0.0035** | **R.GRVGAAGPSGAR.G** |
| **529.7505** | **1057.4864** | **1057.4863** | **0.0002** | **0** | **50** | **0.059** | **R.PGEPGLMGPR.G** |
| **545.2960** | **1088.5774** | **1088.5727** | **0.0047** | **0** | **46** | **0.19** | **R.GVQGPPGPAGPR.G** |
| **552.7698** | **1103.5250** | **1103.5248** | **0.0003** | **0** | **45** | **0.2** | **R.GFPGADGVAGPK.G** |
| **561.2935** | **1120.5724** | **1120.5738** | **-0.0013** | **1** | **50** | **0.069** | **R.GRPGPPGPAGAR.G** |
| **597.2845** | **1192.5544** | **1192.5547** | **-0.0002** | **0** | **57** | **0.012** | [**R.GQAGVMGFPGPK.G**](http://msct.smith.man.ac.uk/mascot/cgi/peptide_view.pl?file=../data/20120830/F291555856.dat&query=1017&hit=1&index=M00043&px=1&section=5&ave_thresh=52) |
| **601.2953** | **1200.5760** | **1200.5775** | **-0.0015** | **0** | **59** | **0.0093** | [**R.GEPGNIGFPGPK.G**](http://msct.smith.man.ac.uk/mascot/cgi/peptide_view.pl?file=../data/20120830/F291555856.dat&query=1069&hit=1&index=M00043&px=1&section=5&ave_thresh=52) |
| **401.8791** | **1202.6155** | **1202.6156** | **-0.0002** | **0** | **44** | **0.28** | **R.SGHPGPVGPAGVR.G** |
| **615.2922** | **1228.5698** | **1228.5693** | **0.0006** | **0** | **55** | **0.02** | **K.GAGPGPMGLMGPR.G** |
| **619.8065** | **1237.5984** | **1237.5979** | **0.0005** | **0** | **41** | **0.49** | **R.GFPGTPGLPGFK.G** |
| **620.3245** | **1238.6344** | **1238.6255** | **0.0089** | **0** | **79** | **7.7e-05** | **R.GLPGSPGNVGPAGK.E** |
| **629.7996** | **1257.5846** | **1257.5837** | **0.0009** | **0** | **51** | **0.056** | [**K.GLTGSPGSPGPDGK.T**](http://msct.smith.man.ac.uk/mascot/cgi/peptide_view.pl?file=../data/20120830/F291555856.dat&query=1272&hit=3&index=M00043&px=1&section=5&ave_thresh=52) |
| **631.3178** | **1260.6210** | **1260.6211** | **-0.0000** | **0** | **53** | **0.034** | **R.GEAGPAGPAGPAGPR.G** |
| **634.3414** | **1266.6682** | **1266.6681** | **0.0002** | **0** | **62** | **0.0035** | [**R.GIPGPVGAAGATGAR.G**](http://msct.smith.man.ac.uk/mascot/cgi/peptide_view.pl?file=../data/20120830/F291555856.dat&query=1309&hit=1&index=M00043&px=1&section=5&ave_thresh=52) |
| **641.3135** | **1280.6124** | **1280.6109** | **0.0015** | **0** | **55** | **0.017** | **K.GEAGPSGPAGPTGAR.G** |
| **664.8279** | **1327.6412** | **1327.6409** | **0.0004** | **0** | **64** | **0.0025** | **R.GFPGLPGPSGEPGK.Q** |
| **667.8443** | **1333.6740** | **1333.6739** | **0.0002** | **1** | **53** | **0.036** | **K.GPAGERGSPGPAGPK.G** |
| **730.3513** | **1458.6880** | **1458.6852** | **0.0029** | **0** | **54** | **0.03** | **R.GSAGPPGATGFPGAAGR.V** |
| **732.3738** | **1462.7330** | **1462.7317** | **0.0013** | **0** | **66** | **0.0019** | **R.GLHGDFGLPGPAGPR.G** |
| **733.3499** | **1464.6852** | **1464.6845** | **0.0007** | **0** | **62** | **0.0044** | **R.GEPGPTGLPGPPGER.G** |
| **737.3416** | **1472.6686** | **1472.6645** | **0.0042** | **0** | **83** | **3.4e-05** | **R.GDGGPPGATGFPGAAGR.T** |
| **737.8603** | **1473.7060** | **1473.7100** | **-0.0039** | **0** | **49** | **0.091** | **R.PGEVGPPGPPGPAGEK.G** |
| **755.8672** | **1509.7198** | **1509.7172** | **0.0026** | **0** | **79** | **8.9e-05** | **R.GAPGAVGAPGPAGATGDR.G** |
| **770.3835** | **1538.7524** | **1538.7511** | **0.0013** | **0** | **48** | **0.11** | **K.SAGGISVPGPMGPSGPR.G** |
| **781.8948** | **1561.7750** | **1561.7737** | **0.0014** | **0** | **62** | **0.0052** | **K.DGLNGLPGPIGPPGPR.G** |
| **781.9199** | **1561.8252** | **1561.8213** | **0.0040** | **0** | **66** | **0.0019** | [**K.GAAGLPGVAGAPGLPGPR.G**](http://msct.smith.man.ac.uk/mascot/cgi/peptide_view.pl?file=../data/20120830/F291555856.dat&query=2730&hit=1&index=M00043&px=1&section=5&ave_thresh=52) |
| **787.9191** | **1573.8236** | **1573.8100** | **0.0136** | **0** | **68** | **0.0013** | **R.GLTGPIGPPGPAGAPGDK.G** |
| **790.3952** | **1578.7758** | **1578.7751** | **0.0008** | **0** | **63** | **0.0043** | **R.GPPGQSGAAGPTGPIGSR.G** |
| **793.8827** | **1585.7508** | **1585.7485** | **0.0024** | **0** | **72** | **0.00044** | **K.GANGAPGIAGAPGFPGAR.G** |
| **810.8854** | **1619.7562** | **1619.7540** | **0.0023** | **0** | **102** | **5e-07** | **R.GPNGEGGATGPPGPPGLR.G** |
| **815.9318** | **1629.8490** | **1629.8475** | **0.0015** | **0** | **68** | **0.0012** | **R.GEPGPVGSVGPVGPVGPR.G** |
| **823.4194** | **1644.8242** | **1644.8220** | **0.0022** | **0** | **86** | **2.2e-05** | [**K.GELGPVGNPGPTGPAGPR.G**](http://msct.smith.man.ac.uk/mascot/cgi/peptide_view.pl?file=../data/20120830/F291555856.dat&query=3138&hit=2&index=M00043&px=1&section=5&ave_thresh=52) |
| **828.4044** | **1654.7942** | **1654.7911** | **0.0032** | **1** | **67** | **0.0014** | **K.GSPGEAGRPGEAGLPGAK.G** |
| **880.3648** | **1758.7150** | **1758.7115** | **0.0035** | **0** | **46** | **0.23** | **K.GEPGSPGENGAPGQMGPR.G** |
| **883.9196** | **1765.8246** | **1765.8231** | **0.0015** | **0** | **59** | **0.0096** | **K.PGEQGVPGDLGAPGPSGAR.G** |
| **888.9374** | **1775.8602** | **1775.8551** | **0.0052** | **1** | **91** | **7.1e-06** | **K.RGPNGEGGATGPPGPPGLR.G** |
| **898.4163** | **1794.8180** | **1794.8133** | **0.0048** | **0** | **72** | **0.00061** | [**R.GPPGNVGSPGVNGAPGEAGR.D**](http://msct.smith.man.ac.uk/mascot/cgi/peptide_view.pl?file=../data/20120830/F291555856.dat&query=3768&hit=1&index=M00043&px=1&section=5&ave_thresh=52) |
| **908.9335** | **1815.8524** | **1815.8574** | **-0.0050** | **0** | **75** | **0.00029** | [**R.GPPGPMGPPGLAGPPGESGR.E**](http://msct.smith.man.ac.uk/mascot/cgi/peptide_view.pl?file=../data/20120830/F291555856.dat&query=3832&hit=3&index=M00043&px=1&section=5&ave_thresh=52) |
| **909.4560** | **1816.8974** | **1816.8956** | **0.0019** | **0** | **62** | **0.0051** | [**R.TGPPGPSGITGPPGPPGAAGK.E**](http://msct.smith.man.ac.uk/mascot/cgi/peptide_view.pl?file=../data/20120830/F291555856.dat&query=3838&hit=1&index=M00043&px=1&section=5&ave_thresh=52) |
| **944.9473** | **1887.8800** | **1887.9149** | **-0.0349** | **1** | **47** | **0.17** | **R.GQAGVMGFPGPKGVAGEPGK.A** |
| **967.4788** | **1932.9430** | **1932.9402** | **0.0028** | **1** | **44** | **0.37** | **K.SGDRGETGPAGPAGPAGPAGVR.G** |
| **1002.4950** | **2002.9754** | **2002.9709** | **0.0046** | **1** | **83** | **4.9e-05** | **K.GEPGPVGVQGPPGPAGEEGKR.G** |
| **1026.4940** | **2050.9734** | **2050.9708** | **0.0026** | **0** | **92** | **6.4e-06** | **R.GEVGPAGPNGFAGPAGAAGQPGAK.G** |
| **692.3602** | **2074.0588** | **2074.0596** | **-0.0008** | **1** | **43** | **0.43** | **R.PGPIGPAGARGEPGNIGFPGPK.G** |
| **1045.0570** | **2088.0994** | **2088.0964** | **0.0031** | **0** | **78** | **0.00013** | **R.GLPGVAGAVGEPGPLGIAGPAGAR.G** |
| **1045.9980** | **2089.9814** | **2089.9778** | **0.0037** | **0** | **63** | **0.0052** | **K.GSPGADGPAGAPGTPGPQGIGGQR.G** |
| **1068.0230** | **2134.0314** | **2134.0291** | **0.0023** | **0** | **62** | **0.0063** | [**K.GEPGVVGAPGTAGPSGPSGLPGER.G**](http://msct.smith.man.ac.uk/mascot/cgi/peptide_view.pl?file=../data/20120830/F291555856.dat&query=4837&hit=1&index=M00043&px=1&section=5&ave_thresh=52) |
| **717.3334** | **2148.9784** | **2148.9713** | **0.0071** | **0** | **50** | **0.09** | **R.GEPGPPGPAGFAGPPGADGQPGAK.G** |
| **1107.9900** | **2213.9654** | **2213.9608** | **0.0047** | **0** | **55** | **0.032** | **K.GDAGAPGAPGSQGAPGLQGMPGER.G** |
| **1125.0730** | **2248.1314** | **2248.1237** | **0.0078** | **0** | **77** | **0.0002** | **R.GYPGNIGPVGAAGAPGPQGAVGPAGK.H** |
| **751.7139** | **2252.1199** | **2252.1186** | **0.0013** | **0** | **66** | **0.0027** | **K.GDAGPAGPAGPTGAPGPIGNVGAPGPK.G** |
| **772.6882** | **2315.0428** | **2315.0415** | **0.0013** | **0** | **45** | **0.33** | **R.GEPGPPGPAGAAGPAGNPGADGQPGAK.G** |
| **1167.5600** | **2333.1054** | **2333.0924** | **0.0130** | **0** | **47** | **0.22** | **K.GEQGPAGPPGFQGLPGPAGPAGEAGK.P** |
| **1183.5590** | **2365.1034** | **2365.0823** | **0.0212** | **0** | **57** | **0.022** | **R.GEQGPAGSPGFQGLPGPAGPPGEAGK.P** |
| **1196.0950** | **2390.1754** | **2390.1714** | **0.0040** | **0** | **40** | **1** | **R.GEVGLPGVSGPVGPPGNPGANGLAGAK.G** |
| **805.7645** | **2414.2717** | **2414.2666** | **0.0050** | **1** | **49** | **0.13** | [**R.GERGLPGVAGAVGEPGPLGIAGPAGAR.G**](http://msct.smith.man.ac.uk/mascot/cgi/peptide_view.pl?file=../data/20120830/F291555856.dat&query=5314&hit=1&index=M00043&px=1&section=5&ave_thresh=52) |
| **1287.1350** | **2572.2554** | **2572.2558** | **-0.0004** | **0** | **55** | **0.036** | **R.GSDGSVGPVGPAGPIGAAGPPGFPGAPGPK.G** |
| **901.7518** | **2702.2336** | **2702.2321** | **0.0014** | **1** | **42** | **0.64** | **R.GAPGDRGEPGPPGPAGFAGPPGADGQPGAK.G** |
| **902.4240** | **2704.2502** | **2704.2478** | **0.0024** | **0** | **60** | **0.012** | [**R.GFSGLQGPPGPPGSPGEQGPSGASGPAGPR.G**](http://msct.smith.man.ac.uk/mascot/cgi/peptide_view.pl?file=../data/20120830/F291555856.dat&query=5758&hit=2&index=M00043&px=1&section=5&ave_thresh=52) |
| **930.4460** | **2788.3162** | **2788.3053** | **0.0109** | **1** | **63** | **0.0051** | **K.GEQGPAGPPGFQGLPGPAGPAGEAGKPGER.G** |
| **931.7889** | **2792.3449** | **2792.3227** | **0.0222** | **0** | **65** | **0.0039** | **K.GHNGLQGLPGLAGQHGDQGAPGSVGPAGPR.G** |
| **949.7983** | **2846.3731** | **2846.3584** | **0.0147** | **1** | **42** | **0.69** | **K.GPKGENGPVGPPGPVGAAGPAGPNGPPGPAGGR.G** |
| **1071.5120** | **3211.5142** | **3211.5018** | **0.0123** | **1** | **55** | **0.038** | **R.GPSGPPGPDGNKGEPGVVGAPGTAGPSGPSGLPGER.G** |
| **1109.8920** | **3326.6542** | **3326.6492** | **0.0050** | **1** | **42** | **0.7** | **K.GPSGEPGTAGPPGSPGPQGLLGAPGILGLPGSRGER.G** |
