# Supplementary material for: A Molecular Phylogeny of Plesiorycteropus Reassigns the Extinct Mammalian Order ‘Bibymalagasia’
Source: PLoS One. 2013 Mar 26;8(3):e59614. doi: 10.1371/journal.pone.0059614 (PMC3608660; doi:10.1371/journal.pone.0059614)
Supplement: Table S10 — Mascot results for Myrmecaphaga bone acid-insoluble protein digest LC-MS data. (DOCX) [file pone.0059614.s013.docx]

Table S10 – Mascot search results of LC-MS data against local database showing observed, expected and calculated molecular weights, the difference between expected and calculated molecular weights (Delta), the number of missed cleavages, peptide ion score, Expect score and peptide sequence (where underline represents modified amino acid) for *Myrmecophaga* bone acid-insoluble protein digest.

| **Observed** | **Mr(expt)** | **Mr(calc)** | **Delta** | **Miss** | **Score** | **Expect** | **Peptide** |
| --- | --- | --- | --- | --- | --- | --- | --- |
| **443.7225** | **885.4304** | **885.4304** | **-0.0000** | **0** | **49** | **0.07** | **R.GSEGPQGVR.G** |
| **446.7534** | **891.4922** | **891.4926** | **-0.0004** | **0** | **47** | **0.12** | **R.PGPIGPAGAR.G** |
| **449.7589** | **897.5032** | **897.5032** | **0.0000** | **0** | **48** | **0.058** | **R.GVVGLPGQR.G** |
| **472.2268** | **942.4390** | **942.4407** | **-0.0016** | **0** | **54** | **0.02** | **R.GPPGSAGSPGK.D** |
| **480.7660** | **959.5174** | **959.4825** | **0.0350** | **0** | **40** | **0.71** | **R.PGPPGPPGPR.G** |
| **488.7468** | **975.4790** | **975.4444** | **0.0347** | **0** | **49** | **0.091** | **R.AGVMGPPGSR.G** |
| **529.7502** | **1057.4858** | **1057.4863** | **-0.0004** | **0** | **52** | **0.041** | **R.PGEPGLMGPR.G** |
| **544.7715** | **1087.5284** | **1087.5298** | **-0.0014** | **0** | **54** | **0.026** | **R.GFPGADGVAGPK.G** |
| **550.7820** | **1099.5494** | **1099.5510** | **-0.0015** | **0** | **42** | **0.33** | **R.GLVGEPGPAGSK.G** |
| **369.1905** | **1104.5497** | **1104.5676** | **-0.0180** | **0** | **52** | **0.042** | **R.GVQGPPGPAGPR.G** |
| **573.7946** | **1145.5746** | **1145.5751** | **-0.0004** | **0** | **57** | **0.013** | **R.GLPGTAGLPGMK.G** |
| **580.2905** | **1158.5664** | **1158.5669** | **-0.0005** | **0** | **70** | **0.00064** | **R.GEAGNIGFPGPK.G** |
| **589.2874** | **1176.5602** | **1176.5598** | **0.0005** | **0** | **61** | **0.005** | **R.GQAGVMGFPGPK.G** |
| **591.8096** | **1181.6046** | **1181.6041** | **0.0006** | **0** | **45** | **0.16** | **K.EGPVGLPGIDGR.P** |
| **592.7504** | **1183.4862** | **1183.4854** | **0.0008** | **0** | **40** | **0.54** | **R.DGNPGNDGPPGR.D** |
| **611.8094** | **1221.6042** | **1221.6030** | **0.0012** | **0** | **43** | **0.33** | **R.GFPGTPGLPGFK.G** |
| **621.8013** | **1241.5880** | **1241.5888** | **-0.0008** | **0** | **57** | **0.013** | [**K.GLTGSPGSPGPDGK.T**](http://msct.smith.man.ac.uk/mascot/cgi/peptide_view.pl?file=../data/20121230/F291569701.dat&query=2301&hit=3&index=M00037&px=1&section=5&ave_thresh=52) |
| **627.3276** | **1252.6406** | **1252.6412** | **-0.0005** | **0** | **76** | **0.00015** | **R.GLPGSPGNIGPAGK.E** |
| **634.3396** | **1266.6646** | **1266.6681** | **-0.0034** | **0** | **48** | **0.1** | **R.GIPGPVGAAGATGAR.G** |
| **656.3185** | **1310.6224** | **1310.6215** | **0.0009** | **0** | **61** | **0.0055** | [**K.GETGPSGPAGPTGAR.G**](http://msct.smith.man.ac.uk/mascot/cgi/peptide_view.pl?file=../data/20121230/F291569701.dat&query=2845&hit=1&index=M00037&px=1&section=5&ave_thresh=52) |
| **664.8278** | **1327.6410** | **1327.6409** | **0.0002** | **0** | **77** | **0.00013** | **R.GFPGLPGPSGEPGK.Q** |
| **666.8311** | **1331.6476** | **1331.6470** | **0.0006** | **0** | **81** | **5.3e-05** | **R.GPSGPQGPSGPPGPK.G** |
| **714.3603** | **1426.7060** | **1426.7061** | **-0.0001** | **0** | **50** | **0.064** | **K.GVGLGPGPMGLMGPR.G** |
| **727.3759** | **1452.7372** | **1452.7361** | **0.0011** | **0** | **63** | **0.0038** | **R.GLPGEFGLPGPAGPR.G** |
| **730.3502** | **1458.6858** | **1458.6852** | **0.0007** | **0** | **90** | **7.9e-06** | **R.GSAGPPGATGFPGAAGR.V** |
| **733.3492** | **1464.6838** | **1464.6845** | **-0.0007** | **0** | **61** | **0.0053** | **R.GEPGPTGLPGPPGER.G** |
| **497.5829** | **1489.7269** | **1489.7274** | **-0.0005** | **0** | **72** | **0.00049** | **R.GETGPAGPAGPAGPAGAR.G** |
| **751.3555** | **1500.6964** | **1500.6958** | **0.0007** | **0** | **55** | **0.025** | **R.GDGGPPGVTGFPGAAGR.T** |
| **781.8942** | **1561.7738** | **1561.7737** | **0.0002** | **0** | **58** | **0.012** | **K.DGLNGLPGPIGPPGPR.G** |
| **521.6047** | **1561.7923** | **1561.8213** | **-0.0290** | **0** | **57** | **0.016** | [**K.GAAGLPGVAGAPGLPGPR.G**](http://msct.smith.man.ac.uk/mascot/cgi/peptide_view.pl?file=../data/20121230/F291569701.dat&query=4665&hit=1&index=M00037&px=1&section=5&ave_thresh=52) |
| **783.8619** | **1565.7092** | **1565.7434** | **-0.0342** | **0** | **82** | **5.1e-05** | **R.GPPGESGAAGPSGPIGSR.G** |
| **785.3888** | **1568.7630** | **1568.7617** | **0.0013** | **0** | **46** | **0.18** | **K.STGGISVPGPMGPSGPR.G** |
| **793.8836** | **1585.7526** | **1585.7485** | **0.0042** | **0** | **64** | **0.0029** | [**K.GANGAPGIAGAPGFPGAR.G**](http://msct.smith.man.ac.uk/mascot/cgi/peptide_view.pl?file=../data/20121230/F291569701.dat&query=4904&hit=1&index=M00037&px=1&section=5&ave_thresh=52) |
| **795.9101** | **1589.8056** | **1589.8050** | **0.0007** | **0** | **52** | **0.047** | [**R.GLTGPIGPPGPAGAPGDK.G**](http://msct.smith.man.ac.uk/mascot/cgi/peptide_view.pl?file=../data/20121230/F291569701.dat&query=4958&hit=5&index=M00037&px=1&section=5&ave_thresh=52) |
| **801.9349** | **1601.8552** | **1601.8162** | **0.0390** | **0** | **43** | **0.42** | [**R.GEPGPAGSVGPVGPVGPR.G**](http://msct.smith.man.ac.uk/mascot/cgi/peptide_view.pl?file=../data/20121230/F291569701.dat&query=5058&hit=1&index=M00037&px=1&section=5&ave_thresh=52) |
| **809.3979** | **1616.7812** | **1616.7795** | **0.0018** | **1** | **58** | **0.014** | [**R.GFSGLDGAKGDAGPAGPK.G**](http://msct.smith.man.ac.uk/mascot/cgi/peptide_view.pl?file=../data/20121230/F291569701.dat&query=5156&hit=2&index=M00037&px=1&section=5&ave_thresh=52) |
| **828.4122** | **1654.8098** | **1654.8064** | **0.0035** | **1** | **53** | **0.042** | [**R.GFPGADGVAGPKGPAGER.G**](http://msct.smith.man.ac.uk/mascot/cgi/peptide_view.pl?file=../data/20121230/F291569701.dat&query=5370&hit=1&index=M00037&px=1&section=5&ave_thresh=52) |
| **833.8923** | **1665.7700** | **1665.7595** | **0.0106** | **0** | **56** | **0.019** | **R.GPNGEPGSTGPSGPPGLR.G** |
| **836.4011** | **1670.7876** | **1670.7860** | **0.0017** | **1** | **52** | **0.057** | [**K.GSPGESGRPGEAGLPGAK.G**](http://msct.smith.man.ac.uk/mascot/cgi/peptide_view.pl?file=../data/20121230/F291569701.dat&query=5465&hit=1&index=M00037&px=1&section=5&ave_thresh=52) |
| **851.9406** | **1701.8666** | **1701.8646** | **0.0021** | **2** | **54** | **0.038** | **R.GAAGIPGGKGEKGETGLR.G** |
| **853.8907** | **1705.7668** | **1705.7656** | **0.0013** | **0** | **91** | **7.5e-06** | [**K.DGEAGAQGPPGPAGPAGER.G**](http://msct.smith.man.ac.uk/mascot/cgi/peptide_view.pl?file=../data/20121230/F291569701.dat&query=5712&hit=1&index=M00037&px=1&section=5&ave_thresh=52) |
| **888.3605** | **1774.7064** | **1774.7064** | **0.0000** | **0** | **50** | **0.079** | **K.GEPGSPGENGAPGQMGPR.G** |
| **601.2915** | **1800.8527** | **1800.8504** | **0.0023** | **1** | **65** | **0.003** | **K.GPRGSAGPPGATGFPGAAGR.V** |
| **908.9371** | **1815.8596** | **1815.8574** | **0.0022** | **0** | **87** | **1.6e-05** | [**R.GPPGPMGPPGLAGPPGESGR.E**](http://msct.smith.man.ac.uk/mascot/cgi/peptide_view.pl?file=../data/20121230/F291569701.dat&query=6473&hit=1&index=M00037&px=1&section=5&ave_thresh=52) |
| **912.4073** | **1822.8000** | **1822.7970** | **0.0031** | **0** | **78** | **0.00013** | **K.GEPGSAGPQGPPGPSGEEGK.R** |
| **917.4534** | **1832.8922** | **1832.8905** | **0.0017** | **0** | **65** | **0.0031** | [**R.TGPPGPSGITGPPGPPGAAGK.E**](http://msct.smith.man.ac.uk/mascot/cgi/peptide_view.pl?file=../data/20121230/F291569701.dat&query=6606&hit=2&index=M00037&px=1&section=5&ave_thresh=52) |
| **920.9644** | **1839.9142** | **1839.9116** | **0.0027** | **0** | **46** | **0.19** | **R.VGPPGPSGNAGPPGPPGPVGK.E** |
| **932.4402** | **1862.8658** | **1862.8647** | **0.0012** | **0** | **80** | **8.9e-05** | **K.GEPGPTGIQGPPGPAGEEGK.R** |
| **644.9808** | **1931.9206** | **1931.9198** | **0.0008** | **1** | **61** | **0.0067** | **K.NGDRGETGPAGPAGPAGPAGAR.G** |
| **990.4572** | **1978.8998** | **1978.8981** | **0.0018** | **1** | **76** | **0.00023** | [**K.GEPGSAGPQGPPGPSGEEGKR.G**](http://msct.smith.man.ac.uk/mascot/cgi/peptide_view.pl?file=../data/20121230/F291569701.dat&query=7465&hit=1&index=M00037&px=1&section=5&ave_thresh=52) |
| **1002.4940** | **2002.9734** | **2002.9708** | **0.0026** | **1** | **77** | **0.00019** | **K.GEPGPTGIQGPPGPAGEEGKR.G** |
| **1034.5000** | **2066.9854** | **2066.9658** | **0.0197** | **0** | **76** | **0.00026** | **R.GEVGPAGPNGFAGPAGAAGQPGAK.G** |
| **1053.0040** | **2103.9934** | **2103.9934** | **0.0001** | **0** | **74** | **0.00034** | **K.GSPGADGPAGAPGTPGPQGISGQR.G** |
| **1055.0150** | **2108.0154** | **2108.0135** | **0.0020** | **0** | **83** | **4.4e-05** | [**K.GEPGVLGAPGTAGASGPGGLPGER.G**](http://msct.smith.man.ac.uk/mascot/cgi/peptide_view.pl?file=../data/20121230/F291569701.dat&query=8057&hit=1&index=M00037&px=1&section=5&ave_thresh=52) |
| **1073.9950** | **2145.9754** | **2145.9676** | **0.0079** | **0** | **54** | **0.042** | **R.GAPGPDGNNGAQGPPGPQGVQGGK.G** |
| **1074.0600** | **2146.1054** | **2146.1019** | **0.0036** | **0** | **73** | **0.00052** | **R.GLPGVSGSVGEPGPLGISGPPGAR.G** |
| **717.6598** | **2149.9576** | **2149.9553** | **0.0023** | **0** | **40** | **0.93** | **R.GEPGPPGPAGFAGPPGADGQPGAK.G** |
| **1078.0250** | **2154.0354** | **2154.0342** | **0.0013** | **0** | **56** | **0.023** | **R.GEAGPAGPPGAPGAPGAPGPVGPAGK.N** |
| **1108.4790** | **2214.9434** | **2214.9448** | **-0.0013** | **0** | **72** | **0.00061** | **K.GDAGAPGAPGSQGAPGLQGMPGER.G** |
| **739.3569** | **2215.0489** | **2215.0506** | **-0.0017** | **1** | **43** | **0.51** | **R.GETGPAGRPGEVGPPGPPGPAGEK.G** |
| **1135.5580** | **2269.1014** | **2269.0975** | **0.0039** | **0** | **78** | **0.00015** | **K.GDAGPAGPAGPTGAPGPIGNVGAPGPK.G** |
| **772.6877** | **2315.0413** | **2315.0415** | **-0.0002** | **0** | **51** | **0.08** | [**R.GEPGPPGPAGAAGPAGNPGADGQPGAK.G**](http://msct.smith.man.ac.uk/mascot/cgi/peptide_view.pl?file=../data/20121230/F291569701.dat&query=8710&hit=1&index=M00037&px=1&section=5&ave_thresh=52) |
| **773.0207** | **2316.0403** | **2316.0255** | **0.0148** | **0** | **(43)** | **0.5** | **R.GEPGPPGPAGAAGPAGNPGADGQPGAK.G** |
| **1191.5530** | **2381.0914** | **2381.0772** | **0.0143** | **0** | **42** | **0.6** | **R.GEQGPAGSPGFQGLPGPAGPPGEAGK.P** |
| **1206.0740** | **2410.1334** | **2410.1149** | **0.0185** | **1** | **60** | **0.01** | [**R.GEVGPAGPNGFAGPAGAAGQPGAKGER.G**](http://msct.smith.man.ac.uk/mascot/cgi/peptide_view.pl?file=../data/20121230/F291569701.dat&query=8862&hit=3&index=M00037&px=1&section=5&ave_thresh=52) |
| **829.7429** | **2486.2069** | **2486.2038** | **0.0031** | **1** | **49** | **0.12** | **R.GPPGSAGSPGKDGLNGLPGPIGPPGPR.G** |
| **1274.6090** | **2547.2034** | **2547.1991** | **0.0044** | **0** | **61** | **0.0077** | **R.GNDGATGAAGPPGPTGPAGPPGFPGAVGAK.G** |
| **1332.6320** | **2663.2494** | **2663.2212** | **0.0282** | **0** | **58** | **0.017** | **R.GFSGLQGPPGAPGSPGEQGPSGASGPAGPR.G** |
| **932.1207** | **2793.3403** | **2793.3067** | **0.0336** | **0** | **42** | **0.66** | **K.GHNGLQGLPGLAGQHGDQGAPGSVGPAGPR.G** |
| **951.1285** | **2850.3637** | **2850.3421** | **0.0216** | **1** | **61** | **0.009** | **K.GEQGPAGPPGFQGLPGPAGTTGEVGKPGER.G** |
