# Supplementary material for: A Molecular Phylogeny of Plesiorycteropus Reassigns the Extinct Mammalian Order ‘Bibymalagasia’
Source: PLoS One. 2013 Mar 26;8(3):e59614. doi: 10.1371/journal.pone.0059614 (PMC3608660; doi:10.1371/journal.pone.0059614)
Supplement: Table S11 — Mascot results for Mammut bone acid-insoluble protein digest LC-MS data. (DOCX) [file pone.0059614.s014.docx]

Table S11 – Mascot search results of LC-MS data against local database showing observed, expected and calculated molecular weights, the difference between expected and calculated molecular weights (Delta), the number of missed cleavages, peptide ion score, Expect score and peptide sequence (where underline represents modified amino acid) for *Mammut* bone acid-insoluble protein digest.

| **Observed** | **Mr(expt)** | **Mr(calc)** | **Delta** | **Miss** | **Score** | **Expect** | **Peptide** |
| --- | --- | --- | --- | --- | --- | --- | --- |
| **369.6927** | **737.3708** | **737.3708** | **-0.0000** | **0** | **54** | **0.014** | **R.GPPGPPGK.N** |
| **418.7273** | **835.4400** | **835.4301** | **0.0100** | **0** | **45** | **0.17** | **R.GPAGPQGPR.G** |
| **443.7351** | **885.4556** | **885.4304** | **0.0251** | **0** | **45** | **0.16** | **R.GSEGPQGVR.G** |
| **446.7615** | **891.5085** | **891.4926** | **0.0158** | **0** | **41** | **0.46** | **R.PGPIGPAGAR.G** |
| **462.2594** | **922.5043** | **922.4872** | **0.0170** | **0** | **50** | **0.046** | **R.GPAGPSGPVGK.D** |
| **464.7467** | **927.4789** | **927.4662** | **0.0127** | **0** | **58** | **0.0069** | **R.PGEAGLPGAK.G** |
| **475.7428** | **949.4710** | **949.4618** | **0.0092** | **0** | **48** | **0.08** | **R.PGPPGPPGAR.G** |
| **544.7810** | **1087.5475** | **1087.5298** | **0.0177** | **0** | **61** | **0.0049** | **R.GFPGADGVAGPK.G** |
| **553.2951** | **1104.5757** | **1104.5676** | **0.0081** | **0** | **46** | **0.18** | **R.GVQGPPGPAGPR.G** |
| **558.7617** | **1115.5089** | **1115.4843** | **0.0246** | **0** | **46** | **0.16** | **R.EGAPGAEGSPGR.D** |
| **563.2760** | **1124.5374** | **1124.5211** | **0.0163** | **0** | **55** | **0.018** | **K.TGPPGPAGQDGR.P** |
| **565.8004** | **1129.5862** | **1129.5802** | **0.0060** | **0** | **49** | **0.074** | **R.GLPGTAGLPGMK.G** |
| **567.8184** | **1133.6223** | **1133.6193** | **0.0030** | **0** | **71** | **0.00054** | **R.GVPGPPGAVGAAGK.D** |
| **577.7925** | **1153.5704** | **1153.5728** | **-0.0024** | **0** | **52** | **0.035** | **K.EGPAGLPGIDGR.P** |
| **589.2819** | **1176.5493** | **1176.5598** | **-0.0105** | **0** | **66** | **0.0017** | **R.GQAGVMGFPGPK.G** |
| **601.2854** | **1200.5563** | **1200.5775** | **-0.0213** | **0** | **61** | **0.0047** | **R.GEPGNIGFPGPK.G** |
| **611.7986** | **1221.5826** | **1221.6030** | **-0.0204** | **0** | **58** | **0.01** | **R.GFPGTPGLPGFK.G** |
| **626.3067** | **1250.5989** | **1250.6004** | **-0.0015** | **0** | **58** | **0.0091** | **R.GEAGPAGSAGPAGPR.G** |
| **629.7932** | **1257.5719** | **1257.5837** | **-0.0118** | **0** | **55** | **0.019** | **K.GLTGSPGSPGPDGK.T** |
| **634.3226** | **1266.6306** | **1266.6681** | **-0.0374** | **0** | **66** | **0.0017** | **R.GIPGPVGAAGATGAR.G** |
| **641.3124** | **1280.6103** | **1280.6109** | **-0.0006** | **0** | **77** | **0.00011** | **K.GEAGPSGPAGPTGAR.G** |
| **645.8266** | **1289.6387** | **1289.6364** | **0.0023** | **0** | **68** | **0.0011** | **R.GPAGPQGPSGAPGPK.G** |
| **664.8349** | **1327.6552** | **1327.6409** | **0.0144** | **0** | **62** | **0.0042** | **R.GFPGLPGPSGEPGK.Q** |
| **725.3542** | **1448.6939** | **1448.6896** | **0.0043** | **0** | **63** | **0.0035** | **R.GEPGPTGLPGPPGER.G** |
| **727.4098** | **1452.8051** | **1452.7361** | **0.0690** | **0** | **85** | **2.1e-05** | **R.GIPGEFGLPGPAGPR.G** |
| **729.3471** | **1456.6796** | **1456.6695** | **0.0100** | **0** | **50** | **0.067** | **R.GDGGPPGATGFPGAAGR.T** |
| **730.3501** | **1458.6857** | **1458.6852** | **0.0006** | **0** | **87** | **1.7e-05** | **R.GSAGPPGATGFPGAAGR.V** |
| **739.8509** | **1477.6873** | **1477.6685** | **0.0188** | **0** | **79** | **0.00011** | **R.TGETGASGPPGFAGEK.G** |
| **745.8606** | **1489.7067** | **1489.7049** | **0.0018** | **0** | **55** | **0.024** | **R.PGEVGPPGPPGPAGEK.G** |
| **759.8822** | **1517.7499** | **1517.7587** | **-0.0088** | **0** | **79** | **9.2e-05** | **R.GETGPAGPAGPAGPAGVR.G** |
| **781.8907** | **1561.7669** | **1561.7737** | **-0.0068** | **0** | **90** | **8.5e-06** | **K.DGLNGLPGPIGPPGPR.G** |
| **787.9146** | **1573.8147** | **1573.8100** | **0.0046** | **0** | **81** | **6.7e-05** | **R.GLTGPIGPPGPAGAPGDK.G** |
| **788.9148** | **1575.8150** | **1575.8006** | **0.0144** | **0** | **73** | **0.00042** | **R.GEPGPAGSVGPVGAVGPR.G** |
| **790.4075** | **1578.8005** | **1578.7751** | **0.0254** | **0** | **94** | **3e-06** | **R.GPPGQSGAAGPTGPIGSR.G** |
| **793.8860** | **1585.7575** | **1585.7485** | **0.0090** | **0** | **82** | **4.6e-05** | **K.GANGAPGIAGAPGFPGAR.G** |
| **816.4059** | **1630.7972** | **1630.8064** | **-0.0092** | **0** | **62** | **0.0051** | **K.GEIGPVGNPGPSGPAGPR.G** |
| **845.9046** | **1689.7946** | **1689.7707** | **0.0240** | **0** | **69** | **0.0011** | **K.DGEAGAQGPPGPAGPAGER.G** |
| **870.9532** | **1739.8918** | **1739.8591** | **0.0327** | **0** | **106** | **2.2e-07** | **R.GPPGPAGPPGLAGPPGESGR.E** |
| **875.9216** | **1749.8286** | **1749.8282** | **0.0003** | **0** | **64** | **0.003** | **K.PGEQGVPGDLGAPGPSGAR.G** |
| **876.9252** | **1751.8358** | **1751.8075** | **0.0283** | **0** | **62** | **0.0048** | **R.GPPGAVGSPGVNGAPGEAGR.D** |
| **909.4692** | **1816.9238** | **1816.8956** | **0.0282** | **0** | **59** | **0.011** | **R.TGPPGPAGITGPPGPPGAAGK.E** |
| **1005.9931** | **2009.9717** | **2009.9443** | **0.0275** | **0** | **91** | **8e-06** | **R.GEVGPAGPNGFAGPAGAAGQAGAK.G** |
| **692.3456** | **2074.0149** | **2073.9828** | **0.0321** | **0** | **50** | **0.09** | **K.GSPGADGPAGAPGTPGPQGIGGQR.G** |
| **705.7298** | **2114.1675** | **2114.1120** | **0.0554** | **0** | **95** | **3.3e-06** | **R.GLPGVAGAVGEPGPLGIAGPPGAR.G** |
| **1077.0533** | **2152.0920** | **2152.0549** | **0.0371** | **0** | **72** | **0.0006** | **R.GETGPSGPPGAPGAPGAPGPVGPAGK.S** |
| **759.7117** | **2276.1132** | **2276.1186** | **-0.0053** | **0** | **55** | **0.031** | **R.GYPGNAGPVGTAGAPGPQGPLGPAGK.H** |
| **1177.5433** | **2353.0720** | **2353.0823** | **-0.0102** | **0** | **107** | **2.3e-07** | **K.GEQGPAGPPGFQGLPGPSGTAGEAGK.P** |
| **788.3980** | **2362.1723** | **2362.1401** | **0.0322** | **0** | **53** | **0.051** | **R.GEAGLPGVSGPVGPPGNPGANGLAGAK.G** |
| **850.0899** | **2547.2477** | **2547.1991** | **0.0487** | **0** | **58** | **0.018** | **R.GNDGATGAAGPPGPTGPAGPPGFPGAVGAK.G** |
| **1284.1129** | **2566.2112** | **2566.2048** | **0.0064** | **0** | **121** | **8.1e-09** | **K.GENGPVGPTGPVGAAGPAGPNGPPGPAGSR.G** |
| **863.7622** | **2588.2646** | **2588.2508** | **0.0139** | **0** | **64** | **0.0041** | **R.GSDGSVGPVGPAGPIGSAGPPGFPGAPGPK.G** |
| **902.4431** | **2704.3075** | **2704.2478** | **0.0597** | **0** | **79** | **0.00014** | **R.GFSGLQGPPGPPGSPGEQGPSGASGPAGPR.G** |
| **703.1217** | **2808.4579** | **2808.3176** | **0.1403** | **0** | **48** | **0.15** | **K.GHNGLQGLPGLAGQHGDQGSPGSVGPAGPR.G** |
| **957.1465** | **2868.4176** | **2868.4003** | **0.0174** | **1** | **54** | **0.049** | **R.GLTGPIGPPGPAGAPGDKGEAGPSGPAGPTGAR.G** |
| **1501.6000** | **3001.1854** | **3001.4629** | **-0.2775** | **0** | **56** | **0.028** | **K.GSAGEPGTAGPPGSPGPQGILGPPGILGLPGSR.G** |
| **1525.6978** | **3049.3810** | **3049.3174** | **0.0636** | **0** | **42** | **0.65** | **R.GPPGATGPPGSPGFQGPPGEPGEPGQTGPAGSR.G** |
