# Supplementary material for: A Molecular Phylogeny of Plesiorycteropus Reassigns the Extinct Mammalian Order ‘Bibymalagasia’
Source: PLoS One. 2013 Mar 26;8(3):e59614. doi: 10.1371/journal.pone.0059614 (PMC3608660; doi:10.1371/journal.pone.0059614)
Supplement: Text S1 — Collagen (I) sequence accession numbers. (DOCX) [file pone.0059614.s015.docx]

**Collagen (I) Sequence Accession Numbers**

Collagen α1 (I) and α2 (I) protein sequences were obtained from the UniProt or ENSEMBL (ENS) genome browser for the following organisms (including corresponding gene transcript numbers): chicken (*Gallus gallus*) P02457 (UNI) & ENSGALP00000015687.3, duckbilled platypus (*Ornithorhynchus anatinus*) ENSOANG00000001736 & ENSOANG00000003903, Tasmanian devil (*Sarcophilus harrisii*) ENSSHAT00000015907 & ENSSHAP00000006215.1, wallaby (*Macropus eugenii*) ENSMEUT00000008452 & ENSMEUP00000000860.1, African elephant (*Loxodonta africana*) ENSLAFP00000014382.4, pika (*Ochotona dauurica*) ENSOPRT00000013202 & ENSOPRP00000010581.2, Norway rat (*Rattus norvegicus*) P02454 & ENSRNOP00000016423.4, mouse (*Mus musculus*) P11087 & Q01149 (UNI), rabbit (*Oryctolagus cunniculus*) ENSOCUG00000012881 & ENSOCUG00000016105, bushbaby (*Otolemur garnetti*) ENSOGAG00000024598 & ENSOGAP00000005344.2, gibbon (*Nomascus leucogenys*) ENSNLET00000011502 & ENSNLEP00000018820.1, chimpanzee (*Pan troglodytes*) ENSPTRT00000017231 & ENSPTRP00000033204.3, human (*Homo sapiens*) PO2452 (UNI) & ENSP00000297268.6, tarsier (*Tarsius syrichta*) ENSTSYT00000013018 & ENSTYSG00000008329, Rhesus macaque (*Macaca* mulatta) ENSMMUG00000001467 & H9Z2D1 (UNI), marmoset (*Callithrix jacchus*) ENSCJAT00000007736 & ENSCJAG00000013547, large flying fox (*Pteropus vampyrus*) ENSPVAT00000008600 & ENSPVAP00000008363.1, little brown bat (*Myotis lucifugus)* ENSMLUT00000028458 & ENSMLUP00000014148.2, horse (*Equus caballus*) ENSECAG00000013693 & ENSECAP00000022559.1, cow (*Bos taurus*) P02453 & ENSBTAP00000033771.4, hedgehog (*Erinaceous europaeus*) ENSEEUT00000007941 & ENSEEUP00000010146.1, panda (*Ailuropodo melanoleuca*) ENSAMET00000013220 & ENSAMEP00000018729.1 and dog (*Canis lupus familiaris*) Q9XSJ7 (UNI) & ENSCAFP00000029400.3. American mastodon (*Mammut americanum*), pig (*Sus scrofa*) and sheep (*Ovis aries*) sequences were obtained from Buckley et al. (2011). The sequences from lesser hedgehog tenrec (*Echinops telfairi*) ENSETEG00000017238 & ENSETEP00000009581.1 were not included in the initial (Figure 1) analysis but included in the fossil sequence analysis (Figure 4) due to the fragmentary nature of the sequences available. Prior to phylogenetic analysis, the procollagen sequence for each of the obtained collagen sequences was removed and the sequences manually corrected where possible (i.e., positions that are required to be G were corrected; the presence of anomalous amino acids (e.g., C, or where differed from all other mammals and birds) were replaced with X. In sites where isobaric residues I/L were present, the most common was used throughout for purposes of phylogenetic inference (e.g., positions COL1A1946-I, COL1A2170-I, COL1A2265-L, COL1A2496-L, COL1A2527-L, COL1A2787-L, COL1A2820-I, COL1A2942-L, COL1A2953-L, COL1A21000-I; subscripted number denotes position in chain with respect to human type 1 collagen, subscripted letter denotes amino acid used). The two alpha chain sequences were then concatenated with an R (selected as a cleavage site for trypsin, making the Mascot search results simpler when used as a local proteomics database) following the alpha 1 chain sequence. All other sequences were obtained by mass spectrometry (LC-MS/MS).
