# Supplementary material for: A Molecular Phylogeny of Plesiorycteropus Reassigns the Extinct Mammalian Order ‘Bibymalagasia’
Source: PLoS One. 2013 Mar 26;8(3):e59614. doi: 10.1371/journal.pone.0059614 (PMC3608660; doi:10.1371/journal.pone.0059614)
Supplement: Text S2 — Concatenated collagen sequences. (DOCX) [file pone.0059614.s016.docx]

**Supplementary Concatenated Collagen Sequences**

>Procavia

XXXXXXXXXSAGGISVPGPMGPSGPRXXXXXXXXXXXXXXXXXXXXXXXXXXXXXXXXXX

XXXXXXXXXXXXXXXXXXXXXXXXXXXXXXXXXXXXXXXXXXXXXXXGFSGLDGAKXXXX

XXXXXGEPGSPGENGAPGQMGPRXXXXXXGRPGPPGPAGARXXXXXXXXXXXXXXXXXXX

XXXXXXXXXXXXXXXXXXXXXXXXXXXXXGEPGPPGPAGAAGPAGNPGADGQPGAKGANG

APGIAGAPGFPGARXXXXXXXXXXXXXXXXXXXXXXXXXXXXXXXXXGEPGPVGVQGPPG

PAGEEGKRXXXGEPGPTGLPGPPGERXXXXXXGFPGADGVAGPKGPAGERGSPGPAGPKG

SPGEAGRPGEAGLPGAKGLTGSPGSPGPDGKXXXXXXXXXXXXXXXXXXXXXXGQAGVMG

FPGPKGVAGEPGKXXXXXXXXXXXXXXXXXXXXXXXXXXXXXXXXXXXXXGEQGPAGSPG

FQGLPGPAGPPGEAGKPGEQGVPGDLGAPGPSGARXXXXXXXXXGVQGPPGPAGPRXXXX

XXXXXXXXGDAGAPGAPGSQGAPGLQGMPGERGAAGLPGPKXXXXXXXXXXXXXXXXXXX

XXGLTGPIGPPGPAGAPGDKGEAGPSGPAGPTGARGAPGDRGEPGPPGPAGFAGPPGADG

QPGAKXXXXXXXXXGDAGPAGPAGPTGAPGPIGNVGAPGPKXXXGSAGPPGATGFPGAAG

RXXXXXXXXXXXXXXXXXXXXXXXXXXXXXXXXXXXXPGEVGPPGPPGPAGEKGSPGADG

PAGAPGTPGPQGIGGQRGVVGLPGQRXXXGFPGLPGPSGEPGKXXXXXXXXXXGPPGPMG

PPGLAGPPGESGRXXXXXXXXXXXXXXXXXXXXXXXXXXXXXXXXXXXXXXXXXXXXXXX

XSGDRGETGPAGPAGPAGPAGVRXXXXXXXXXXXXXXXXXXXXXXXXXXXGFSGLQGPPG

PPGSPGEQGPSGASGPAGPRXXXXXXXXXXXDGLNGLPGPIGPPGPRXXXXXXXXXXXXX

XXXXXXXXXXXXXXXXXXXXXXXXXXXXXXXXXXXXXXXXXXXG-A-GPGPMGLMGPRXX

XXXXXXXXXXXXXXXXXXXXXXXXXXXXXXXXXXXXXXXXXXXXXXXXXXXXXXXXXXXX

XXXXGFPGTPGLPGFKXXXXXXXXXXXXXXXXXXXXXXXXXXXXXXXXXXXXXXXXXXXX

XGRVGAAGPSGARGSDGSVGPVGPAGPIGAAGPPGFPGAPGPKGELGPVGNPGPTGPAGP

RGEVGLPGVSGPVGPPGNPGANGLAGAKGAAGLPGVAGAPGLPGPRGIPGPVGAAGATGA

RXXXXXXXXXXXXXXXXXXXXXXXXXXXXXXXXXXXXXXRGPNGEGGATGPPGPPGLRXX

XXXXXXXXXXXXXXXXXXXXXXXXXXXXXXXXXXXXXXXPGEPGLMGPRGLPGSPGNVGP

AGKXXXXXXXXXXXXPGPIGPAGARGEPGNIGFPGPKXXXXXXXXXXXXXXXXXXXXXXX

XXXXXXXXXXXXXXXXXXXXXXGEQGPAGPPGFQGLPGPAGPAGEAGKPGERGLHGDFGL

PGPAGPRXXXGPPGQSGAAGPTGPIGSRGPSGPPGPDGNKGEPGVVGAPGTAGPSGPSGL

PGERXXXXXXXXXXXXXXXXXXXXXXXXXXXXXXGAPGAVGAPGPAGATGDRGEAGPAGP

AGPAGPRXXXXXXGEVGPAGPNGFAGPAGAAGQPGAKXXXXXXGPKGENGPVGPPGPVGA

AGPAGPNGPPGPAGGRGDGGPPGATGFPGAAGRTGPPGPSGITGPPGPPGAAGKXXXXXX

XXXXXXXXXXXXXXXXXXXXXXXXXGPSGEPGTAGPPGSPGPQGLLGAPGILGLPGSRGE

RGLPGVAGAVGEPGPLGIAGPAGARGPPGNVGSPGVNGAPGEAGRXXXXXXXXXXXXXXX

XXXXXXXGYPGNIGPVGAAGAPGPQGAVGPAGKXXXXGEPGPVGSVGPVGPVGPRGPSGT

QGLRXXXXXXXXXXXXXXXXXXGHNGLQGLPGLAGQHGDQGAPGSVGPAGPRXXXXXXXX

XXXXXXSGHPGPVGPAGVRXXXXXXXXXXXXXXXXXXXXXXXXXXXXXXXXXXXXXXX

>Mammut

XXXXXXXXXXXXXXXXXXXXXXXXXXXXXXXXXXXXXXXXXXXXXXXXXXXXXXXXXXXG

PPGPPGKXXXXXXXXXXXXXXXXXXXXXXXXXGLPGTAGLPGMKXXXXXXXXXXXXXXXX

XXXXXXXXXXXXXXXXXXXXXXXXXXXXXXXXXXXXXXXXXGNDGATGAAGPPGPTGPAG

PPGFPGAVGAKXXXXXXXXXGSEGPQGVRGEPGPPGPAGAAGPAGNPGADGQPGAKGANG

APGIAGAPGFPGARGPAGPQGPSGAPGPKXXXXXXXXXXXXXXXXXXGEPGPIGIQGPPG

PAGEEGKXXXXGEPGPTGLPGPPGERXXXXXXGFPGADGVAGPKXXXXXXXXXXXXXXXX

XXXXXXXPGEAGLPGAKGLTGSPGSPGPDGKTGPPGPAGQDGRPGPPGPPGARGQAGVMG

FPGPKXXXXXXXXXXXXGVPGPPGAVGAAGKDGEAGAQGPPGPAGPAGERXXXXXXXXXX

XXXXXXXXXXXXXXXXPGEQGVPGDLGAPGPSGARXXXXXXXXXGVQGPPGPAGPRXXXX

XXXXXXXXXXXXXXXXXXXXXXXXXXXXXXXXXXXXXXXXXXXXXXXXXXXXXXXXXXXX

XXGLTGPIGPPGPAGAPGDKGEAGPSGPAGPTGARXXXXXXXXXXXXXXXXXXXXXXXXX

XXXXXXXXXXXXXXXXXXXXXXXXXXXXXXXXXXXXXXXXXXXXGSAGPPGATGFPGAAG

RXXXXXXXXXXXXXXXXXXXXXXXXXXXXXXXXXXXXPGEVGPPGPPGPAGEKGSPGADG

PAGAPGTPGPQGIGGQRXXXXXXXXXXXXGFPGLPGPSGEPGKXXXXXXXXXXGPPGPAG

PPGLAGPPGESGREGAPGAEGSPGRXXXXXXXXXXGETGPSGPPGAPGAPGAPGPVGPAG

KXXXXGETGPAGPAGPAGPAGVRGPAGPQGPRXXXXXXXXXXXXXXXXXXGFSGLQGPPG

PPGSPGEQGPSGASGPAGPRXXXXXXXXXXXDGLNGLPGPIGPPGPRXXXXXXXXXXXXX

XXXXXXXXXXXXXXXXXXXXXXXXXXXXXXXXXXXXXXXXXXXXXXXXXXXXXXXXXXGP

PGATGPPGSPGFQGPPGEPGEPGQTGPAGSRXXXXXXXXXXXXXXXXXXXXXXXXXXXXX

XXXXGFPGTPGLPGFKXXXXXXXXXXXXXXXXXXXXXXXXXXXXXXXXXXXXXXXXXXXX

XXXXXXXXXXXXXGSDGSVGPVGPAGPIGSAGPPGFPGAPGPKGELGPVGNPGPSGPAGP

RXXXXXXXXXXXXXXXXXXXXXXXXXXXXXXXXXXXXXXXXXXXXXGIPGPVGAAGATGA

RXXXXXXXXXXXXXXXXXXXXXXXXXXXXXXXXXXXXXXXXXXXXXXXXXXXXXXXXXXX

XXXXXXXXXXXXXXXXXXXXXXXXXXXXXXXXXXXXXXXXXXXXXXXXXXXXXXXXXXXX

XXXEGPAGLPGIDGRPGPIGPAGARGEPGNIGFPGPKXXXXXXXXXXXXXXXXXXXXXXX

XXXXXXXXXXXXXXXXXXXXXXGEQGPAGPPGFQGLPGPSGTAGEAGKXXXXGLPGEFGL

PGPAGPRXXXGPPGQSGAAGPTGPIGSRXXXXXXXXXXXXXXXXXXXXXXXXXXXXXXXX

XXXXXXXXXXXXXXXXXXXXXXXXXXXXXXXXXXXXXXXXXXXXXXXXXXXXGEAGPAGS

AGPAGPRXXXXXXGEVGPAGPNGFAGPAGAAGQAGAKXXXXXXXXXGENGPVGPTGPVGA

AGPAGPNGPPGPAGSRGDGGPPGATGFPGAAGRTGPPGPAGITGPPGPPGAAGKXXXXXX

XXXXXXXXXTGETGASGPPGFAGEKGSSGEPGTAGPPGSPGPQGLLGPPGILGLPGSRXX

XGLPGVAGAVGEPGPLGIAGPPGARGPPGAVGSPGVNGAPGEAGRXXXXXXXXXXXXXXX

XXXXXXXGYPGNAGPVGTAGAPGPQGPLGPAGKXXXXGEPGPAGSVGPVGAVGPRXXXXX

XXXXXXXXXXXXXXXXXXXXXXGHNGLQGLPGLAGQHGDQGSPGSVGPAGPRGPAGPSGP

VGKXXXXXXXXXXXXXXXXXXXXXXXXXXXXXXXXXXXXXXXXXXXXXXXXXXXXXXX

>Orycteropus

XXXXXXXXXSAG-ISVPGPMGPSGPRXXXXXXXXXXXXXXXXXXXXXXXXXXXXXXXXXX

XXXXXXXXXXXXXXXXXXXXXXXXXXXXXXXXXXXXXXXXXXXXXXXGFSGLDGAKGDVG

PAGPKGEPGSPGENGAPGQMGPRXXXXXXXXXXXXXXXXXXGNDGATGAAGPPGPTGPAG

PPGFPGAVGAKGEVGPQGTRXXXXXXXXXXXXXXXXXXXXXXXXXXXXXXXXXXXXGANG

APGIAGAPGFPGARGPSGPQGPSGAPGPKXXXXXXXXXXXXXXXXXXXXXXXXXXXXXXX

XXXXXXXXXXXGEPGPTGLPGPPGERXXXXXXGFPGSDGVAGPKXXXXXXXXXXXXXXXG

SPGEAGRPGEAGLPGAKGLTGSPGSPGPDGKXXXXXXXXXXXXXXXXXXXXXXGQAGVMG

FPGPKXXXXXXXXXXXXXXXXXXXXXXXXXXXXXXXXXXXXXXXXXXXXXXXXXXXXXXX

XXXXXXXXXXXXXXXXXXXXXXXXXXXXXXXXXXXXXXXXXXXXGVQGPPGPAGPRXXXX

XXXXXXXXGDAGAPGAPGSQGAPGLQGMPGERGAAGLPGPKXXXXXXXXXXXXXXXXXXX

XXXXXXXXXXXXXXXXXXXXXXXXXXXXXXXXXXXGAPGDRGEPGPPGPAGFAGPPGADG

QPGAKGEPGDAGAKGDAGPPGPAGPTGAPGPIGNVGAPGPKGARGSAGPPGATGFPGAAG

RVGPPGPSGNAGPPGPPGPAGKXXXXXXXXXXXXXXXXXXXXXXXXXXXXXXXGSPGADG

PAGAPGTPGPQGIGGQRGVVGLPGQRXXXGFPGLPGPSGEPGKXXXXXXXXXXGPPGPMG

PPGLAGPPGESGRXXXXXXXXXXXXXXXXXXXXXXGETGPAGPPGAPGAPGAPGPVGPAG

KSGDRGETGPAGPAGPIGPAGVRXXXXXXXXXXXXXXXXXXXXXXXXXXXGFSGLQGPPG

PPGSPGEQGPSGASGPAGPRGPPGSAGAPGKDGLNGLPGPIGPPGPRXXXXXXXXXXXXX

XXXXXXXXXXXXXXXXXXXXXXXXXXXXXXXXXXXXXXXXXXXGVGLGPGPMGLMGPRXX

XXXXXXXXXXXXXXXXXXXXXXXXXXXXXXXXXXXXXXXXXXXXXXXXXXXXXXXXXXXX

XXXXGFPGTPGLPGFKGIRXXXXXXXXXXXXXXXXXXXXXXXXXXXXXXXXXXXXXXXXX

XXXXXXXXXXXXXGSDGSVGPVGPAGPIGSAGPPGFPGAPGPKGELGPVGNPGPSGPAGP

RXXXXXXXXXXXXXXXXXXXXXXXXXXXGAAGLPGVAGAPGLPGPRGIPGPVGAAGATGA

RXXXXXXXXXXXXXXXXXXXXXXXXXXXXXXXXXXXXXXXGPNGEVGSAGPPGPPGLRXX

XXXXXXXXXXXXAGVMGPPGSRGQTGPAGVRXXXXXXXXXXXXXXXXXXGLPGSPGNVGP

AGKEGPVGLPGIDGRPGPVGPAGARGEPGNIGFPGPKXXXXXXXXXXXXXXXXXXXXXXX

XXXXXXXXXXXXXXXXXXXXXXXXXXXXXXXXXXXXXXXXXXXXXXXXXXXXGLHGDFGL

PGPAGPRXXXGPPGQSGAAGPTGPIGSRGPSGPPGPDGNKGEPGVVGAPGTAGPSGPSGL

PGERXXXXXXXXXXXXXXXXXXXXXXXXXXXXXXGAPGAIGAPGPAGATGDRGEAGPAGP

AGPAGPRGSPGERGEVGPAGPNGFAGPAGAAGQPGAKXXXXXXXXXXXXXXXXXXXXXXX

XXXXXXXXXXXXXXXXXXXXXXXXXXXXXXXXXTGPPGPSGITGPPGPPGAAGKXXXXXX

XXXXXXXXXTGETGASGPPGFTGEKGPSGEPGTAGPPGSPGPQGLLGAPGILGLPGSRGE

RGLPGVAGAVGEPGPLGISGPAGARXXXXXXXXXXXXXXXXXXXXXXXXXXXXXXXXXXX

XXXXXXXXXXXXXXXXXXXXXXXXXXXXXXXXXHGNRGEPGPAGSVGPVGPVGPRXXXXX

XXXXXXXXXXXXXXXXXXXXXXXXXXXXXXXXXXXXXXXXXXXXXXXXXXXXXXXXXXXX

XXXXXXSGHPGAVGPAGVRXXXXXXXXXXXXXXXXXXXXXXXXXXXXXXXXXXXXXXX

>Echinops

XXXXXXXXXXXXXXXXXXXXGPSGPRGLPGPPGAPGPQGFQGPAGEPGEPGASGPMGPRG

PPGPPGKNGDDGEAGKPGRAGERGPPGPQGARGLPGTAGLPGMKGHRXXXXXXXXXXXXX

XXXXXXXXXXXXXXXXXXXXXXXXXXXXXXXXXXXXXXXXXXXXXXXXXXXXXXXXXXXX

XXXXXXXXXXXGESgPQGTRGSEGPQGVRGEPGPPGPAGAAGPAGNPGXXGQPGXKGANG

APGIAGAPGFPGARGPSGPQGPSGAPGPKGNSGEPGAPGNKGDAGAKGEPGPTGVQGPPG

PAGEEGKRGARGEPGPSGLPGPPGERGGPGSRGFPGSDGVAGPKGPAGERGSPGPAGPKG

SPGESGRPGEAGLPGAKXXXXXXXXXXXXXXXXXXXXXXXXXXXXXXXXXXXXXXXXXXX

XXXXXXXXXXXXXXXXXXXXXXXXXXXXXXXXXXXXXXXXXXXXXXXXXXXXXXXXXXXX

XXXXXXXXXXXXXXXXXXXXGVPGDLGAPGPSGARGERGFPGERGVQGPPGPAGPRGSNG

APGNDGAKGDAGAPGAPGSQGAPGLQGMPGERGAAGLPGPKGDRGDAGPKGADGXXXXXX

XXXXXXXXXXXXXXXXXXXXGESGPSGPAGPTGARGAPGDRGEPGPAGPAGFAGPPGADG

QPGAKGEPGDAGAKGDAGLSGPAGPTGAPGPIGNVGAPGPKGARGSAGPPGATGXPGAAX

RVXXXXPSXXXXXXXXXXXXXXXXXXXXXXXXXXXXXXXXXXXXXXXXXXXXXXXXXXXX

XXXXXXXXXXXXXXXXXXXXXXXXXXXXXXXXXXXXXXXXXXXXXXXXXXXXXXXXXXXX

XXXXXXXXXXXXXXXXXXXXXXXXXXXXXXXXXXXXXXXXXXXXXXXXXXXXXXXXXXXX

XXXXXXXXGPAGPAGPIGPVGARGPSGPQGARGDKGETGEQGDRGMKGHRGFSGLQGPPG

LPGSPGEQGPSGASGPAGPRGPPGSAGSPGKDGLSGLPGPIGPPGPRGRTGDAGPVGPPG

PPGPPGPPGPPSGGFDFSFMPQPPQEKAHDGGRYYRARQYDGKGVGLGPGPMGLMGPRGP

PGANGPPGPPGFNGPAGEPGEPGQTGPAGSRGPAGPPGKAGEDGHPGNPERSGERGVVGX

XGARGFPGTPGLPGFKGIRGHNGLDGLKGQPGAAGVKGXXXXXXXXGTXGQXGARGLPGE

RGRVGGSGPAGARGSDGSVGPVGPAGPIGSAGPPGFPGAPGPKGELGPVGNPGPSGPAGP

RGEVGLPGVSGPVGPPGNPGANGLAGAKGAAGLPGVAGAPGLPGPRGIPGPAGSAGATGA

RGLVGEPGPAGSKGESGSKGEPGSAGPQGPAXXXXXXXXXXXXXXXXXXXXXXXXXXXGS

PGSRGLPGADGRAGVMGPPGNRGASGPAGSRGPSGDSGRPGEPGLMGPRGLPGSPGNVGP

AGKEGLGGLPGIDGRPGPTGPAGARGEPGNIGFPGPKGPTGDAGKPGDKGHAGLAGPRGA

PGPDGNNGAQGPPGPQGVQGGKGEQGPAGPPGFQGLPGPAGPTGEVGKPGERGLHGEFGL

PGPAGPRGERGPPGQSGAAGPTGSIGSRGPSGPPGPDGNKGEPGVVGAPGTAGASGPGGL

PGERGAAGVPGGKGEKGETGLRGEIGNTGRDGARGAPGAVGAPGPAGATGDRGEAGAAGP

AGPAGPRGSPGERGEVGPAGPNGFAGPAGAAGQAGPKGERGTKGPKGENGAVGPTGPIGS

AGPSGPNGPPGPAGSRGDGGPPXXXXXXXXXXXXXXXXXXGITGPPGPPGAAGKEGLRGP

RGDQGPVGRTGETGASGPTGFTGEKGPSGEPGTAGPPGTPGPQGILGPPGILGLPGSRGE

RGLPGVAGSLGEPGPLGISGPPGARGXXGAVGNPGVNGAXXXXXXXGNPGSDGPPGRDGL

PGHKGERGYPGNAGPVGNAGAPGPHGSVGPAGKYGNRGEPGPAGSVGPVGAVGPRGPSGP

QGPRGDKGEAGEKGPRGLTGFKGHNGLQGLPGLAGQHGDQGSPGTVGPAGPRGPAGPSGP

AGKDGRSGHPGAVGPAGVRGSQGSQGPSGPPGPPGPPGPPGVSGGGYDFGYDGDFYRA

>Setifer

XXXXXXXXXXXXXXXXXXXXXXXXXXXXXXXXXXXXXXXXXXXXXXXXXXXXXXXXXXXX

XXXXXXXXXXXXXXXXXXXXXXXXXXXXXXXXXXXXXXXXXXXXXXXXXXXXXXXXXXXX

XXXXXXXXXXXXXXXXXXXXXXXXXXXXXXXXXXXXXXXXXGNDGATGAAGPPGPTGPAG

PPGFPGAVGAKGEAGPQGTRXXXXXXXXXXXXXXXXXXXXXXXXXXXXXXXXXXXXGANG

APGIAGAPGFPGARXXXXXXXXXXXXXXXXXXXXXXXXXXXXXXXXXGEPGPAGVQGPPG

PAGEEGKRXXXGEPGPSGLPGPPGERXXXXXXXXXXXXXXXXXXXXXXXXXXXXXXXXXX

XXXXXXXXXXXXXXXXXGLTGSPGSPGPDGKXXXXXXXXXXXXXXXXXXXXXXGQAGVMG

FPGPKXXXXXXXXXXXXXXXXXXXXXXXXXXDGEAGAQGPPGPAGPAGERXXXXXXXXXX

XXXXXXXXXXXXXXXXPGEQGVPGDLGAPGPSGARXXXXXXXXXGVQGPPGPAGPRXXXX

XXXXXXXXGDAGAPGAPGSQGAPGLQGMPGERXXXXXXXXXXXXXXXXXXXXXXXXXXXX

XXGLTGPIGPPGPAGSPGDKGESGPSGPAGPTGARXXXXXXGEPGPAGPAGFAGPPGADG

QPGAKXXXXXXXXXGDAGPPGPAGPTGAPGPIGNVGAPGPKXXXGSAGPPGATGFPGAAG

RVGPPGPSGNAGPPGPPGPAGKXXXXXXXXXXXXXXXPGEVGPPGPPGPAGEKXXXXXXX

XXXXXXXXXXXXXXXXXGVVGLPGQRXXXGFPGLPGPSGEPGKXXXXXXXXXXGPPGPMG

PPGLAGPPGESGRXXXXXXXXXXXXXXXXXXXXXXGETGPAGPPGAPGAPGAPGPVGPAG

KXXXXGETGPAGPAGPIGPVGARGPTGPQGPRXXXXXXXXXXXXXXXXXXGFSGLQGPPG

PPGSPGEQGPSGASGPAGPRXXXXXXXXXXXXXXXXXXXXXXXXXXXXXXXXXXXXXXXX

XXXXXXXXXXXXXXXXXXXXXXXXXXXXXXXXXXXXXXXXXXXXXXXXXXXXXXXXXXXX

XXXXXXXXXXXXXXXXXXXXXXXXXXXXXXXXXXXXXXXXXXXXXXXXXXXXXXXXXXXX

XXXXXXXXXXXXXXXXXXXXXXXXXXXXXXXXXXXXXXXXXXXXXXXXXXXXXXXXXXXX

XXXXXXXXXXXXXXXXXXXXXXXXXXXXXXXXXXXXXXXXXXXGELGPVGNPGPSGPAGP

RXXXXXXXXXXXXXXXXXXXXXXXXXXXGAAGLPGVAGAPGLPGPRGIPGPAGSAGATGA

RGLVGEPGPAGSKGESGSKXXXXXXXXXXXXXXXXXXXXRGPNGEAGSAGPAGPPGLRXX

XXXXXXXXXXXXXXXXXXXXXXXXXXXXXXXXXXXXXXXPGEPGLMGPRGLPGSPGNVGP

AGKEGLGGLPGIDGRXXXXXXXXXXGEPGNIGFPGPKXXXXXXXXXXXXXXXXXXXXXXX

XXXXXXXXXXXXXXXXXXXXXXGEQGPAGPPGFQGLPGPAGTTGEVGKPGERGLHGEFGL

PGPAGPRXXXGPPGQSGAAGPTGSIGSRXXXXXXXXXXXXGEPGVVGAPGTAGASGPGGL

PGERXXXXXXXXXXXXXXXXXXXXXXXXXXXXXXGAPGAVGAPGPAGATGDRGEAGAAGP

AGPAGPRXXXXXXGEVGPAGPNGFAGPAGAAGQPGAKGERXXXGPKGENGAVGPTGPIGS

AGPSGPNGPPGPAGSRGDGGPPGMTGFPGAAGRTGPPGPSGITGPPGPPGAAGKXXXXXX

XXXXXXXXXTGETGASGPTGFTGEKXXXXXXXXXXXXXXXXXXXXXXXXXXXXXXXXXXX

XGLPGVAGSLGEPGPLGISGPPGARXXXXXXXXXXXXXXXXXXXXXXXXXXXXXXXXXXX

XXXXXXXGYPGNAGPVGNAGAPGPHGSVGPAGKXXXXXXXXXXXXXXXXXXXXXXXXXXX

XXXXXXXXXXXXXXXXXXXXXXXXXXXXXXXXXXXXXXXXXXXXXXXXXXXXXXXXXXXX

XXXXXXXXXXXXXXXXXXXXXXXXXXXXXXXXXXXXXXXXXXXXXXXXXXXXXXXXXX

>Ambylosomus

XXXXXXXXXXXXXXXXXXXXXXXXXXXXXXXXXXXXXXXXXXXXXXXXXXXXXXXXXXXX

XXXXXXXXXXXXXXXXXXXXXXXXXXXXXXXXGLPGTAGLPGMKXXXGFSGLDGAKGDSG

PAGPKGEPGSPGENGAPGQMGPRXXXXXXXXXXXXXXXXXXGNDGATGAAGPPGPTGPAG

PPGFPGAVGAKGEAGPQGARXXXXXXXXXXXXXXXXXXXXXXXXXXXXXXXXXXXXGANG

APGIAGAPGFPGARGPSGPQGPSGAPGPKXXXXXXXXXXXXXXXXXXGEPGPTGVQGPPG

PAGEEGKRXXXGEPGPTGLPGPPGERXXXXXXXXXXXXXXXXXXXXXXXXXXXXXXXXXX

XXXXXXXXXXXXXXXXXXXXXXXXXXXXXXXXXXXXXXXXXXXPGPPGPPGSRGQAGVMG

FPGPKXXXXXXXXXXXXXXXXXXXXXXXXXXDGEAGAQGPPGPAGPAGERXXXXXXXXXX

XXXXXXXXXXXXXXXXPGEQGVPGDLGAPGPSGARXXXXXXXXXGVQGPPGPAGPRXXXX

XXXXXXXXGDAGAPGAPGSQGAPGLQGMPGERGAAGLPGPKXXXXXXXXXXXXXXXXXXX

XXGLTGPIGPPGPAGATGDKGESGPSGPAGPTGARXXXXXXGEPGPAGPAGFAGPPGADG

QPGAKGEPGDAGAKGDAGPAGPAGPTGAPGPIGNVGAPGPKXXXGSAGPPGATGFPGAAG

RVGPPGPSGNAGPPGPPGPAGKXXXXXXXXXXXXXXXPGEVGPPGPPGPAGEKGSPGADG

PAGAPGTPGPQGIGGQRGVVGLPGQRXXXGFPGLPGPSGEPGKXXXXXXXXXXGPPGPMG

PPGLAGPPGESGRXXXXXXXXXXXXXXXXXXXGDRGETGPAGPPGAPGAPGAPGPVGPAG

KXXXXGETGPAGPAGPVGPAGARGPAGPQGPRXXXXXXXXXXXXXXXXXXGFSGLQGPPG

PPGSPGEQGPSGASGPAGPRGPPGSAGTPGKDGLNGLPGPIGPPGPRXXXXXXXXXXXXX

XXXXXXXXXXXXXXXXXXXXXXXXXXXXXXXXXXXXXXQYDAKGVGLGPGPMGLMGPRXX

XXXXXXXXXXXXXXXXXXXXXXXXXXXXXXXXXXXXXXXXXXXXXXXXXXXXXXXXXXXX

XXXXGFPGTPGLPGFKXXXXXXXXXXXXXXXXXXXXXXXXXXXXXXXXXXXXXXXXXXXX

XXXXXXXXXXXXXXXXXXXXXXXXXXXXXXXXXXXXXXXXXXXGELGPVGNPGPSGPAGP

RGEVGLPGVSGPVGPPGNPGANGLAGAKGAAGLPGVAGAPGLPGPRXXXXXXXXXXXXXX

XXXXXXXXXXXXXXXXXXXXXXXXXXXXXXXXXXXXXXXXXXXXXXXXXXXXXXXXXXXX

XXXXXXXXXXXXXXXXXXXXXXXXXXXXXXXXXXXXXXXPGEPGLMGPRXXXXXXXXXXX

XXXEGLGGLPGIDGRPGPTGPAGARGEPGNIGFPGPKXXXXXXXXXXXXXXXXXXXXXXX

XXXXXXXXXXXXXXXXXXXXXXGEQGPAGPPGFQGLPGPAGTTGEVGKPGERGLHGEFGL

PGPAGPRXXXGPPGQSGAAGPTGPIGSRXXXXXXXXXXXXXXXXXXXXXXXXXXXXXXXX

XXXXXXXXXXXXXXXXXXXXXXXXXXXXXXXXXXGAPGAVGAPGPAGATGDRXXXXXXXX

XXXXXXXXXXXXXGEVGPAGPNGFAGPAGAAGQPGAKGERXXXXXXGENGPVGPTGPVGA

AGPSGPNGPPGPAGSRGDGGPPGVTGFPGAAGRTGPPGPAGITGPPGPPGAAGKXXXXXX

XXXXXXXXXTGETGASGPPGFTGEKXXXXXXXXXXXXXXXXXXXXXXXXXXXXXXXXXXX

XGLPGVAGSLGEPGPLGIAGPPGARGPPGAVGNPGVNGAPGEAGRXXXXXXXXXXXXXXX

XXXXXXXXXXXXXXXXXXXXXXXXXXXXXXXXXHGNRGEPGPAGSVGPVGAVGPRXXXXX

XXXXXXXXXXXXXXXXXXXXXXXXXXXXXXXXXXXXXXXXXXXXXXXXXXXXXXXXXXXX

XXXXXXXXXXXXXXXXXXXXXXXXXXXXXXXXXXXXXXXXXXXXXXXXXXXXXXXXXX

>Petrodromus

XXXXXXXXXSAG-VSVPGPMGPSGPRXXXXXXXXXXXXXXXXXXXXXXXXXXXXXXXXXX

XXXXXXXXXXXXXXXXXXXXXXXXXXXXXXXXGLPGTAGLPGMKXXXGFSGLDGAKGDAG

PAGPKGEPGSPGENGAPGQMGPRXXXXXXXXXXXXXXXXXXGNDGATGAAGPPGPTGPAG

PPGFPGAVGAKGEAGPQGARXXXXXXXXXGEPGPPGPAGAAGPAGNPGADGQPGAKGANG

APGIAGAPGFPGARGPSGPQGPSGAPGPKXXXXXXXXXXXXXXXXXXGEPGPAGVQGPPG

PAGEEGKRXXXGEPGPTGLPGPPGERXXXXXXGFPGADGVAGPKXXXXXXGSPGPAGPKG

SPGEAGRPGEAGLPGAKGLTGSPGSPGPDGKTGPPGPAGQDGRPGPPGPPGSRGQAGVMG

FPGPKXXXXXXXXXXXXGVPGPPGAVGQAGKDGEAGAQGPPGPAGPAGERGEQGPAGSPG

FQGLPGPAGPPGEAGKXXXXXXXXXXXXXXXXXXXXXXXXXXXXGVQGPPGPAGPRXXXX

XXXXXXXXXXXXXXXXXXXXXXXXXXXXXXXXGAAGLPGPKGDRXXXXXXXXXXXXXXXX

XXGLTGPIGPPGPAGAPGDKGETGPSGPAGPTGARXXXXXXXXXXXXXXXXXXXXXXXXX

XXXXXXXXXXXXXXGDAGPPGPAGPTGAPGPIGNVGAPGVKGARGSAGPPGATGFPGAAG

RVGPPGPSGNAGPPGPPGPAGKXXXXXXXGETGPAGRPGEVGPPGPPGPAGEKGSPGADG

PAGAPGTPGPQGIGGQRGVVGLPGQRXXXGFPGLPGPSGEPGKXXXXXXXXXXGPPGPMG

PPGLAGPPGESGRXXXXXXXXXXXXXXXXXXXGDRGETGPAGPPGAPGAPGAPGPVGPAG

KSGDRGETGPAGPAGPIGPAGARXXXXXXXXXXXXXXXXXXXXXXXXXXXGFSGLQGPPG

PPGSPGEQGPSGASGPAGPRGPPGSAGSPGKDGLNGLPGPIGPPGPRXXXXXXXXXXXXX

XXXXXXXXXXXXXXXXXXXXXXXXXXXXXXXXXXXXXXQYDGKGVGGGPGPMGLMGPRXX

XXXXXXXXXXXXXXXXXXXXXXXXXXXXXXXXXXXXXXXXXXXXXXXXXXXXXXXXXXXX

XXXXXXXXXXXXXXXXXXXGHNGLDGLKGQAGAPGVKXXXXXXXXXXXXXXXXXXXXXXX

XXXXXXXXXXXXXGSDGSVGPVGPAGPIGSAGPPGFPGAPGPKGELGPVGNPGPSGPAGP

RXXXXXXXXXXXXXXXXXXXXXXXXXXXXXXXXXXXXXXXXXXXXXXXXXXXXXXXXXXX

XXXXXXXXXXXXXXXXXXXGEPGSAGPQGPPGPSGEEGKRXXXXXXXXXXXXXXXXXXXX

XXXXGLPGADGRAGVMGPPGSRGATGPAGVRXXXXXXXXPGEPGLMGPRGLPGSPGNVGP

AGKXXXXXXXXXXXXXXXXXXXXXXGEPGNIGFPGPKXXXXXXXXXXXXXXXXXXXXXGA

PGPDGNNGAQGSPGPQGVQGGKXXXXXXXXXXXXXXXXXXXXXXXXXXXXXXGLHGEFGL

PGPAGPRXXXXXXXXXXXXXXXXXXXXXXXXXXXXXXXXXXXXXXXXXXXXXXXXXXXXX

XXXXGAAGIPGGKGEKGETGLRXXXXXXXXXXXXGAPGAVGAPGPAGATGDRGEAGAAGP

AGPAGPRXXXXXXXXXXXXXXXXXXXXXXXXXXXXXXXXXXXXXXXXXXXXXXXXXXXXX

XXXXXXXXXXXXXXXXXXXXXXXXXXXXXXXXXXXXXXXXXXXXXXXXXXXXXXXXXXXX

XXXXXXXXXTGETGASGPPGFAGEKGPSGEPGTAGPPGSPGPQGLLGAPGILGLPGSRGE

RGLPGVAGSVGEPGPLGIAGPPGARGPPGAVGNPGVNGAPGEAGRDGNPGSDGPPGRDGL

PGHKXXXXXXXXXXXXXXXXXXXXXXXXXXXXXHGNRGEPGPAGSVGPTGAVGPRXXXXX

XXXXXXXXXXXXXXXXGLPGLKGHNGLQGLPGLAGHHGDQGAPGTVGPAGPRXXXXXXXX

XXXXXXXXXXXXXXXXXXXXXXXXXXXXXXXXXXXXXXXXXXXXXXXXXXXXXXXXXX

>Plesiorycteropus

XXXXXXXXXXXXXXXXXXXXXXXXXXXXXXXXXXXXXXXXXXXXXXXXXXXXXXXXXXXX

XXXXXXXXXXXXXXXXXXXXXXXXXXXXXXXXXXXXXXXXXXXXXXXXXXXXXXXXXXXX

XXXXXXXXXXXXXXXXXXXXXXXXXXXXXXXXXXXXXXXXXGNDGATGAAGPPGPTGPAG

PPGFPGAVGAKXXXXXXXXXXXXXXXXXXXXXXXXXXXXXXXXXXXXXXXXXXXXXGANG

APGIAGAPGFPGARGPSGPQGPSGAPGPKXXXXXXXXXXXXXXXXXXGEPGPTGVQGPPG

PAGEEGKRXXXGEPGPTGLPGPPGERGGPGSRGFPGADGIAGPKXXXXXXXXXXXXXXXX

XXXXXXXXXXXXXXXXXXXXXXXXXXXXXXXXXXXXXXXXXXXPGPPGPPGSRGQAGVMG

FPGPKXXXXXXXXXXXXXXXXXXXXXXXXXXDGEAGAQGAPGPAGPAGERXXXXXXXXXX

XXXXXXXXXXXXXXXXXXXXXXXXXXXXXXXXXXXXXXXXXXXXGVQGPPGPAGPRXXXX

XXXXXXXXGDAGAPGAPGSQGAPGLQGMPGERXXXXXXXXXXXXXXXXXXXXXXXXXXXX

XXGLTGPIGPPGPAGAPGDKGESGPSGPAGPTGARXXXXXXGEPGPPGPAGFAGPPGADG

QPGAKXXXXXXXXXGDAGPPGPAGPTGAPGPIGNVGAPGPKXXXGSAGPPGATGFPGAAG

RVGPPGPSGNAGPPGPPGPAGKXXXXXXXXXXXXXXXPGEAGPPGPPGPAGEKGSPGADG

PAGAPGTPGPQGIGGQRGVVGLPGQRXXXGFPGLPGPSGEPGKXXXXXXXXXXGPPGPMG

PPGLAGPPGESGRXXXXXXXXXXXXXXXXXXXXXXXXXXXXXXXXXXXXXXXXXXXXXXX

XSGDRGETGPAGPAGPIGPVGARXXXXXXXXXXXXXXXXXXXXXXXXXXXGFSGLQGPPG

APGSPGEQGPSGASGPAGPRGPPGSAGSPGKDGLNGLPGPIGPPGPRXXXXXXXXXXXXX

XXXXXXXXXXXXXXXXXXXXXXXXXXXXXXXXXXXXXXXXXXXGVGLGPGPMGLMGPRXX

XXXXXXXXXXXXXXXXXXXXXXXXXXXXXXXXXXXXXXXXXXXXXXXXXXXXXXXXXXXX

XXXXGFPGTPGLPGFKGNRXXXXXXXXXXXXXXXXXXXXXXXXXXXXXXXXXXXXXXXXX

XXXXXXXXXXXXXXXXXXXXXXXXXXXXXXXXXXXXXXXXXXXGELGPVGNPGPSGPAGP

RGELGLPGVSGPVGPPGNPGANGLAGAKGATGLPGVAGAPGLPGPRGIPGPVGAAGAAGA

RXXXXXXXXXXXXXXXXXXXXXXXXXXXXXXXXXXXXXXXGPNGEAGSAGPVGPPGLRXX

XXXXXXXXXXXXXXXXXXXXXXXXXXXXXXXXXXXXXXXPGEPGLMGPRGLPGSPGNVGP

AGKEGLGGLPGIDGRXXXXXXXXXXGEPGNIGFPGPKXXXXXXXXXXXXGNVGLAGPRXX

XXXXXXXXXXXXXXXXXXXXXXGEQGPAGPPGFQGLPGPAGPAGEVGKPGERGLHGEFGL

PGPAGPRXXXGPPGQSGAAGPTGSIGSRGPSGPPGPDGNKGEPGVVGAPGTAGPSGPSGL

PGERXXXXXXXXXXXXXXXXXXXXXXXXXXXXXXXXXXXXXXXXXXXXXXXXGEAGAAGP

AGPAGPRXXXXXXGEVGPAGPNGFAGPAGAAGQPGAKGERXXXGPKGENGPVGPTGPIGS

AGPSGPNGPPGPAGSRGDGGPPGMTGFPGAAGRTGPSGPSGITGPPGPPGAAGKXXXXXX

XXXXXXXXXTGETGASGPPGFTGEKXXXXXXXXXXXXXXXXXXXXXXXXXXXXXXXXXGE

RGLPGVAGALGEPGPLGIAGPPGARGPPGNVGNPGVNGAPGEAGRXXXXXXXXXXXXXXX

XXXXXXXGYPGNAGPVGAAGAPGPHGSVGPAGKHGNRGEPGPAGSVGPVGAVGPRXXXXX

XXXXXXXXXXXXXXXXXXXXXXXXXXXXXXXXXXXXXXXXXXXXXXXXXXXXGPAGPSGP

PGKXXXXXXXXXXXXXXXXXXXXXXXXXXXXXXXXXXXXXXXXXXXXXXXXXXXXXXX

>Myrmecophaga

XXXXXXXXXSTGGISVPGPMGPSGPRXXXXXXXXXXXXXXXXXXXXXXXXXXXXXXXXXX

XXXXXXXXXXXXXXXXXXXXXXXXXXXXXXXXGLPGTAGLPGMKXXXGFSGLDGAKGDAG

PAGPKGEPGSPGENGAPGQMGPRXXXXXXXXXXXXXXXXXXGNDGATGAAGPPGPTGPAG

PPGFPGAVGAKXXXXXXXXXGSEGPQGVRGEPGPPGPAGAAGPAGNPGADGQPGAKGANG

APGIAGAPGFPGARGPSGPQGPSGPPGPKXXXXXXXXXXXXXXXXXXGEPGPTGIQGPPG

PAGEEGKRXXXGEPGPTGLPGPPGERXXXXXXGFPGADGVAGPKGPAGERXXXXXXXXXG

SPGESGRPGEAGLPGAKGLTGSPGSPGPDGKXXXXXXXXXXXXPGPPGPPGPRGQAGVMG

FPGPKXXXXXXXXXXXXXXXXXXXXXXXXXXDGEAGAQGPPGPAGPAGERGEQGPAGSPG

FQGLPGPAGPPGEAGKXXXXXXXXXXXXXXXXXXXXXXXXXXXXGVQGPPGPAGPRXXXX

XXXXXXXXGDAGAPGAPGSQGAPGLQGMPGERXXXXXXXXXXXXXXXXXXXXXXXXXXXX

XXGLTGPIGPPGPAGAPGDKGETGPSGPAGPTGARXXXXXXGEPGPPGPAGFAGPPGADG

QPGAKXXXXXXXXXGDAGPAGPAGPTGAPGPIGNVGAPGPKGPRGSAGPPGATGFPGAAG

RVGPPGPSGNAGPPGPPGPVGKXXXXXXXGETGPAGRPGEVGPPGPPGPAGEKGSPGADG

PAGAPGTPGPQGISGQRGVVGLPGQRXXXGFPGLPGPSGEPGKXXXXXXXXXXGPPGPMG

PPGLAGPPGESGRXXXXXXXXXXXXXXXXXXXXXXGEAGPAGPPGAPGAPGAPGPVGPAG

KNGDRGETGPAGPAGPAGPAGARXXXXXXXXXXXXXXXXXXXXXXXXXXXGFSGLQGPPG

APGSPGEQGPSGASGPAGPRGPPGSAGSPGKDGLNGLPGPIGPPGPRXXXXXXXXXXXXX

XXXXXXXXXXXXXXXXXXXXXXXXXXXXXXXXXXXXXXXXXXXGVGLGPGPMGLMGPRXX

XXXXXXXXXXXXXXXXXXXXXXXXXXXXXXXXXXXXXXXXXXXXXXXXXXXXXXXXXXXX

XXXXGFPGTPGLPGFKXXXXXXXXXXXXXXXXXXXXXXXXXXXXXXXXXXXXXXXXXXXX

XXXXXXXXXXXXXXXXXXXXXXXXXXXXXXXXXXXXXXXXXXXXXXXXXXXXXXXXXXXX

XXXXXXXXXXXXXXXXXXXXXXXXXXXXGAAGLPGVAGAPGLPGPRGIPGPVGAAGATGA

RGLVGEPGPAGSKXXXXXXGEPGSAGPQGPPGPSGEEGKRGPNGEPGSTGPSGPPGLRXX

XXXXXXXXXXXXAGVMGPPGSRXXXXXXXXXXXXXXXXXPGEPGLMGPRGLPGSPGNIGP

AGKEGPVGLPGIDGRPGPIGPAGARGEAGNIGFPGPKXXXXXXXXXXXXXXXXXXXXXGA

PGPDGNNGAQGPPGPQGVQGGKGEQGPAGPPGFQGLPGPAGTTGEVGKPGERGLPGEFGL

PGPAGPRXXXGPPGESGAAGPSGPIGSRXXXXXXXXXXXXGEPGVLGAPGTAGASGPGGL

PGERGAAGIPGGKGEKGETGLRXXXXXXXXXXXXXXXXXXXXXXXXXXXXXXXXXXXXXX

XXXXXXXXXXXXXGEVGPAGPNGFAGPAGAAGQPGAKGERXXXXXXGENGVVGPTGPIGA

AGPSGPNGPPGPAGSRGDGGPPGVTGFPGAAGRTGPPGPSGITGPPGPPGAAGKXXXXXX

XXXXXXXXXXXXXXXXXXXXXXXXXXXXXXXXXXXXXXXXXXXXXXXXXXXXXXXXXXXX

XGLPGVSGSVGEPGPLGISGPPGARXXXXXXXXXXXXXXXXXXXXDGNPGNDGPPGRXXX

XXXXXXXXXXXXXXXXXXXXXXXXXXXXXXXXXXXXXGEPGPAGSVGPVGPVGPRXXXXX

XXXXXXXXXXXXXXXXXXXXXXGHNGLQGLPGLAGQHGDQGAPGSVGPAGPRXXXXXXXX

XXXXXXXXXXXXXXXXXXXXXXXXXXXXXXXXXXXXXXXXXXXXXXXXXXXXXXXXXX

>Tamandua

XXXXXXXXXXXXXXXXXXXXXXXXXXXXXXXXXXXXXXXXXXXXXXXXXXXXXXXXXXXX

XXXXXXXXXXXXXXXXXXXXXXXXXXXXXXXXGLPGTAGLPGMKXXXXXXXXXXXXXXXX

XXXXXXXXXXXXXXXXXXXXXXXXXXXXXXXXXXXXXXXXXGNDGATGAAGPPGPTGPAG

PPGFPGAVGAKXXXXXXXXXXXXXXXXXXXXXXXXXXXXXXXXXXXXXXXXXXXXXGANG

APGIAGAPGFPGARGPAGPQGPSGPPGPKXXXXXXXXXXXXXXXXXXGEPGPTGIQGPPG

PAGEEGKXXXXGEPGPTGLPGPPGERXXXXXXGFPGADGVAGPKXXXXXXXXXXXXXXXX

XXXXXXXXXXXXXXXXXGLTGSPGSPGPDGKXXXXXXXXXXXXXXXXXXXXXXGQAGVMG

FPGPKXXXXXXXXXXXXXXXXXXXXXXXXXXDGEAGAQGAPGPAGPAGERGEQGPAGSPG

FQGLPGPAGPPGEAGKPGEQGVPGDLGAPGPSGARXXXXXXXXXGVQGPPGPAGPRXXXX

XXXXXXXXGDAGAPGAPGSQGAPGLQGMPGERXXXXXXXXXXXXXXXXXXXXXXXXXXXX

XXGLTGPIGPPGPAGAPGDKXXXXXXXXXXXXXXXXXXXXXGEPGPPGPAGFAGPPGADG

QPGAKXXXXXXXXXGDAGPPGPAGPSGPPGPIGNVGAPGPKXXXGSAGPPGATGFPGAAG

RXXXXXXXXXXXXXXXXXXXXXXXXXXXXXXXXXXXXXXXXXXXXXXXXXXXXGSPGADG

PAGAPGTPGPQGISGQRGVVGLPGQRXXXGFPGLPGPSGEPGKXXXXXXXXXXGPPGPMG

PPGLAGPPGESGRXXXXXXXXXXXXXXXXXXXXXXGEAGPAGPPGAPGAPGAPGPVGPAG

KXXXXGETGPAGPAGPAGPAGARGPAGPQGPRXXXXXXXXXXXXXXXXXXGFSGLQGPPG

APGSPGEQGPSGASGPAGPRXXXXXXXXXXXDGLNGLPGPIGPPGPRXXXXXXXXXXXXX

XXXXXXXXXXXXXXXXXXXXXXXXXXXXXXXXXXXXXXXXXXXXXXXXXXXXXXXXXXXX

XXXXXXXXXXXXXXXXXXXXXXXXXXXXXXXXXXXXXXXXXXXXXXXXXXXXXXXXXXXX

XXXXXXXXXXXXXXXXXXXXXXXXXXXXXXXXXXXXXXXXXXXXXXXXXXXXXXXXXXXX

XXXXXXXXXXXXXGSDGSVGPVGPAGPIGSAGPPGFPGAPGPKGELGPVGNTGPSGPAGP

RXXXXXXXXXXXXXXXXXXXXXXXXXXXGAAGLPGVAGAPGLPGPRXXXXXXXXXXXXXX

XXXXXXXXXXXXXXXXXXXXXXXXXXXXXXXXXXXXXXXXGPNGEPGSTGPSGPPGLRXX

XXXXXXXXXXXXXXXXXXXXXXXXXXXXXXXXXXXXXXXPGEPGLMGPRXXXXXXXXXXX

XXXEGPVGLPGIDGRXXXXXXXXXXGEPGNIGFPGPKXXXXXXXXXXXXXXXXXXXXXGA

PGPDGNNGAQGPPGPQGVQGGKGEQGPAGPPGFQGLPGPAGTTGEVGKPGERGLPGEFGL

PGPAGPRXXXGPPGESGAVGPSGAIGSRXXXXXXXXXXXXGEPGVLGAPGTAGASGPGGL

PGERXXXXXXXXXXXXXXXXXXXXXXXXXXXXXXXXXXXXXXXXXXXXXXXXXXXXXXXX

XXXXXXXXXXXXXXXXXXXXXXXXXXXXXXXXXXXXXXXXXXXGPKGENGVVGPTGPIGA

AGPSGPNGPPGPAGSRGDGGPPGVTGFPGAAGRTGPPGPAGITGPPGPPGAAGKXXXXXX

XXXXXXXXXTGELGAVGPPGFTGEKXXXXXXXXXXXXXXXXXXXXXXXXXXXXXXXXXXX

XGLPGTSGALGEPGPLGISGPPGARXXXXXXXXXXXXXXXXXXXXXXXXXXXXXXXXXXX

XXXXXXXXXXXXXXXXXXXXXXXXXXXXXXXXXXXXXXXXXXXXXXXXXXXXXXXXXXXX

XXXXXXXXXXXXXXXXXXXXXXXXXXXXXXXXXXXXXXXXXXXXXXXXXXXXXXXXXXXX

XXXXXXXXXXXXXXXXXXXXXXXXXXXXXXXXXXXXXXXXXXXXXXXXXXXXXXXXXX

>Manis

XXXXXXXXXXXXXXXXXXXXXXXXXXXXXXXXXXXXXXXXXXXXXXXXXXXXXXXXXXXG

PPGPPGKNGDDGEAGKPGRXXXXXXXXXXXXXXXXXXXXXXXXXXXXGFSGLDGAKXXXX

XXXXXGEPGSPGENGAPGQMGPRXXXXXXXXXXXXXXXXXXGNDGATGAAGPPGPTGPAG

PPGFPGAVGAKXXXXXXXXXXXXXXXXXXGEPGPPGPAGAAGPAGNPGADGQPGAKGANG

APGIAGAPGFPGARGPSGPQGPSGPPGPKXXXXXXXXXXXXXXXXXXGEPGPTGIQGPPG

PAGEEGKXXXXGEPGPTGLPGPPGERXXXXXXGFPGADGVAGPKGPAGERGSPGPAGPKG

SPGEAGRPGEAGLPGAKXXXXXXXXXXXXXXTGPTGPAGQDGRPGPPGPPGSRGQAGVMG

FPGPKXXXXXXXXAGERGVPGPPGAVGPAGKDGEAGAQGPPGPAGPAGERGEQGPAGSPG

FQGLPGPAGPSGEAGKPGEQGVPGDLGAPGPSGARXXXGFPGERGVQGPPGPAGPRXXXX

XXXXXXXXGDAGAPGAPGSQGAPGLQGMPGERGAAGLPGPKXXXXXXXXXXXXXXXXXXX

XXGLTGPIGPPGPAGATGDKGESGPSGPAGPTGARXXXXXXGEPGPPGPAGFAGPPGADG

QPGAKXXXXXXXXXGDAGPAGPAGPAGPPGPIGNVGAPGPKGARGSAGPPGATGFPGAAG

RVGPPGPSGNAGPPGPPGPVGKXXXXXXXXXXXXXXXXXXXXXXXXXXXXXXXGSPGADG

PAGAPGTPGPQGIAGQRGVVGLPGQRXXXGFPGLPGPSGEPGKXXXXXXXXXXGPPGPMG

PPGLAGPPGESGRXXXXXXXXXXXXXXXXXXXXXXGETGPAGPPGAPGAPGAPGPVGPAG

KNGDRGETGPAGPAGPIGPVGARGPAGPQGPRXXXXXXXXXXXXXXXXXXGFSGLQGPPG

PPGSPGEQGPSGASGPAGPRGPPGSAGSPGKDGLNGLPGPIGPPGPRXXXXXXXXXXXXX

XXXXXXXXXXXXXXXXXXXXXXXXXXXXXXXXXXXXXXXXXXXXXXXXXXXXXXXXXXXX

XXXXXXXXXXXXXXXXXXXXXXXXXXXXXXXXXXXXXXXXXXXXXXXXXXXXXXXXXXXX

XXXXGFPGTPGLPGFKGNRXXXXXXXXXXXXXXXXXXGEPGAPGENGTPGQTGARXXXXX

XGRVGAPGPAGARGSDGSVGPVGPAGPIGSAGPPGFPGAPGPKGELGPVGNTGPSGPAGP

RGEVGLPGLSGPVGPPGNPGANGLTGAKGAAGLPGVAGAPGLPGPRXXXXXXXXXXXXXX

XGLVGEPGPAGSKXXXXXXGEPGSAGAQGPPGPSGEEGKRXXXXXXXXXXXXXXXXXXXX

XXXXXXXXXXXXXXXXXXXXXXXXXXXXXXXXXXXXXXXPGEPGLMGPRGFPGSPGNVGP

AGKEGPVGLPGIDGRPGPVGPAGPRGEPGNIGFPGPKXXXXXXXXXXXXXXXXXXXXXGA

PGPDGNNGAQGPPGPQGVQGGKGEQGPAGPPGFQGLPGPAGPAGEVGKPGERGLPGEFGL

PGPAGPRXXXGPPGESGAAGPSGPIGSRXXXXXXXXXXXXGEPGVLGAPGTAGASGPGGL

PGERXXXXXXXXXXXXXXXXXXXXXXXXXXXXXXGAPGAVGAPGPAGATGDRGEAGPAGP

AGPAGPRXXXXXXGEVGPAGPNGFAGPAGAAGQPGAKXXXXXXGPKGENGPVGPTGPVGS

AGPSGPNGPPGPAGSRGDGGPPGAVGFPGAAGRTGPPGPAGITGPPGPPGAAGKXXXXXX

XXXXXXXXXTGETGASGPPGFTGEKGPSGEPGTAGPPGTAGPQGLLGAPGILGLPGSRGE

RGLPGVAGSLGEPGPLGISGPPGARGPSGGVGNPGVNGAPGEAGRXXXXXXXXXXXXXXX

XXXXXXXGYPGNIGPVGAVGAPGPHGPVGPTGKXXXXXXXXXXXXXXXXXXXXXXXXXXX

XXXXXXXXXXXXXXXXXXXXXXXXXXXXXXXXXXXXXXXXXXXXXXXXXXXXGPAGPTGP

VGKXXXXXXXXXXXXXXXXXXXXXXXXXXXXXXXXXXXXXXXXXXXXXXXXXXXXXXX

>Gallus

QMSYGYDEKSAG-VAVPGPMGPAGPRGLPGPPGAPGPQGFQGPPGEPGEPGASGPMGPRG

PAGPPGKNGDDGEAGKPGRPGERGPPGPQGARGLPGTAGLPGMKGHRGFSGLDGAKGQPG

PAGPKGEPGSPGENGAPGQMGPRGLPGERGRPGPSGPAGARGNDGAPGAAGPPGPTGPAG

PPGFPGAAGAKGETGPQGARGSEGPQGSRGEPGPPGPAGAAGPAGNPGADGQPGAKGATG

APGIAGAPGFPGARGPSGPQGPSGAPGPKGNSGEPGAPGNKGDTGAKGEPGPAGVQGPPG

PAGEEGKRGARGEPGPAGLPGPAGERGAPGSRGFPGADGIAGPKGPPGERGSPGAVGPKG

SPGEAGRPGEAGLPGAKGLTGSPGSPGPDGKTGPPGPAGQDGRPGPAGPPGARGQAGVMG

FPGPKGAAGEPGKPGERGAPGPPGAVGAAGKDGEAGAQGPPGPTGPAGERGEQGPAGAPG

FQGLPGPAGPPGEAGKPGEQGVPGNAGAPGPAGARGERGFPGERGVQGPPGPQGPRGANG

APGNDGAKGDAGAPGAPGNEGPPGLEGMPGERGAAGLPGAKGDRGDPGPKGADGAPGKDG

LRGLTGPIGPPGPAGAPGDKGEAGPPGPAGPTGARGAPGDRGEPGPPGPAGFAGPPGADG

QPGAKGETGDAGAKGDAGPPGPAGPTGAPGPAGAVGAPGPKGARGSAGPPGATGFPGAAG

RVGPPGPSGNIGLPGPPGPAGKKGSKGPRGETGPAGRPGEPGPAGPPGPPGEKGSPGADG

PIGAPGTPGPQGIAGQRGVVGLPGQRGERGFPGLPGPSGEPGKQGPSGASGERGPPGPMG

PPGLAGPPGEAGREGAPGAEGAPGRDGAAGPKGDRGETGPAGPPGAPGAPGAPGPVGPAG

KNGDRGETGPAGPAGPPGPAGARGPAGPQGPRGDKGETGEQGDRGMKGHRGFSGLQGPPG

PPGAPGEQGPSGASGPAGPRGPPGSAGAAGKDGLNGLPGPIGPPGPRGRTGEVGPVGPPG

PPGPPGPPGPPSGGFDFSFLPQPPQEKAHDGGRYYRARQYDPKAADFGPGPMGLMGPRGP

PGASGPPGPPGFQGVPGEPGEPGQTGPQGPRGPPGPPGKAGEDGHPGKPGRPGERGVAGP

QGARGFPGTPGLPGFKGIRGHNGLDGQKGQPGTPGTKGEPGAPGENGTPGQPGARGLPGE

RGRIGAPGPAGARGSDGSAGPTGPAGPIGAAGPPGFPGAPGAKGELGPAGNVGPTGPAGP

RGEIGLPGSSGPVGPPGNPGANGLPGAKGAAGLPGVAGAPGLPGPRGIPGPPGPAGPSGA

RGLVGEPGPAGAKGESGNKGEPGAAGPPGPPGPSGEEGKRGSNGEPGSAGPPGPAGLRGV

PGSRGLPGADGRAGVMGPAGNRGASGPVGAKGPNGDAGRPGEPGLMGPRGLPGQPGSPGP

AGKEGPVGFPGADGRVGPIGPAGNRGEPGNIGFPGPKGPTGEPGKPGEKGNVGLAGPRGA

PGPEGNNGAQGPPGVTGNQGAKGETGPAGPPGFQGLPGPSGPAGEAGKPGERGLHGEFGV

PGPAGPRGERGLPGESGAVGPAGPIGSRGPSGPPGPDGNKGEPGNVGPAGAPGPAGPGGI

PGERGVAGVPGGKGEKGAPGLRGDTGATGRDGARGLPGAIGAPGPAGGAGDRGEGGPAGP

AGPAGARGIPGERGEPGPVGPSGFAGPPGAAGQPGAKGERGPKGPKGETGPTGAIGPIGA

SGPPGPVGAAGPAGPRGDAGPPGMTGFPGAAGRVGPPGPAGITGPPGPPGPAGKDGPRGL

RGDVGPVGRTGEQGIAGPPGFAGEKGPSGEAGAAGPPGTPGPQGILGAPGILGLPGSRGE

RGLPGIAGATGEPGPLGVSGPPGARGPSGPVGSPGPNGAPGEAGRDGNPGNDGPPGRDGA

PGFKGERGAPGNPGPSGALGAPGPHGQVGPSGKPGNRGDPGPVGPVGPAGAFGPRGLAGP

QGPRGEKGEPGDKGHRGLPGLKGHNGLQGLPGLAGQHGDQGPPGNNGPAGPRGPPGPSGP

PGKDGRNGLPGPIGPAGVRGSHGSQGPAGPPGPPGPPGPPGPNGGGYEVGFDAEYYRA

>Ornithorhynchus

QMAYGYDEKAGGGMSVPGPMGPSGPRGLPGPPGSPGPQGFQGPPGEPGEPGASGPMGPRG

PAGPPGKNGDDGEAGKPGRPGERGPPGPQGARGLPGTAGLPGMKGHRGFSGLDGAKGDSG

PAGPKGEPGSAGENGAPGQMGPRGLPGERGRPGPSGPAGARGNDGAPGAAGPPGPTGPAG

PPGFPGAVGAKGEAGAQGSRGSEGPQGARGEPGPPGPAGAAGPSGNPGSDGQPGAKGANG

APGIAGAPGFPGARGPSGPQGPSGGPGPKGNSGEPGAPGNKGDPGAKGEPGPVGVQGPPG

PSGEEGKRGSRGEPGPTGLPGPAGERGGPGSRGFPGADGVAGPKGPAGERGSPGPAGPKG

SPGEAGRPGEAGLPGAKGMPGSPGGPGSDGKPGPPSPAGQGGRPGPPGSQGPRGQPGVMG

FPGPKGNDGAPGKNGERGGPGAPGGPGAAGKDGEAGAQGPPGPAGPAGERGEQGPSGSPG

FQGLPGPSGPAGESGKPGEQFPPGEAGAPSPSGARGERGFPGERGVQGPAGPQGPRGSNG

APGNDGAKGDAGAPGAPGGQGPPGLQGMPGERGAAGLPGAKGDRGDAGPKGGDGAPGKDG

IRGLTGPIGPPGPAGNPGDKGESGPSGPAGPTGARGAPGDRGEPGPPGPAGFAGPPGADG

QPGAKGETGDSGAKGDAGPPGPAGPTGAPGPAGNVGAPGPKGARGSAGPPGATGFPGAAG

RVGPPGPSGNPGPPGSAGPSGKDGPPGPAGNTGPPGGPGGVGPKGEAGQPGEKGSPGDDG

PSGPDGPPGPQGLAGQRGIVGLPGQRGERGFPGLPGPSGEPGKQGAPGGAGDRGPPGPVG

PPGLTGPSGEPGREGNPGSDGPPGRDGAAGVKGDRGETGPAGPPGAPGAPGAPGPVGPAG

KNGDRGETGPSGPAGPAGPAGARGPSGPQGPRGDKGETGEQGDRGMKGHRGFSGLQGPPG

PPGSPGEQGPSGASGPAGPRGPLGPHGPPGKDGVSGHPGPIGPPGPRGNRGETGPAGSPG

HPGNPGPPGPPSGGFDFSFxPQPPQEKxHDGGRYYRARQYDAKAADMGPGPMGLMGPRGP

PGASGAPGAQGFQGPPGEPGEPGQSGPAGSRGPAGPPGKSGEDGHPGKPGRSGERGVVGP

QGARGFPGTPGLPGFKGIRGHNGLDGQKGQPGTPGVKGEPGAPGENGSPGQSGARGLPGE

RGRIGGAGPTGARGSDGSVGPVGPAGPiGSAGPPGFPGAPGPKGElGAVGNTGPAGPAGP

RGELGLPGVSGPVGPAGNPGANGLAGAKGAAGLPGVAGAPGLPGPRGIPGPSGPSGPSGP

RGLVGEPGPAGSKGESGSKGEPGSAGAQGPPGPNGEEGKRGPNGEPGSTGPTGPPGLRGV

PGSRGLPGADGRAGGMGPAGNRGSAGPSGARGPSGDSGRPGEPGLVGPRGLPGFPGNVGP

AGKEGPVGLPGSEGRPGPTGPAGARGEPGNIGFPGPKGPNGEPGKSGERGHAGLAGSRGA

PGPDGNNGAqGPPGPTGNQGGKgEQSPAGPPgfqGLPGPSGPAGESGKPGERGLPGEFGL

PGPAGPRGERGPPGESGAAGPTGPLGNRGPSGPPGPDGNKGEPGVAGAPGNAGPAGSGGL

PGERGAVGVPGGKGEKGEPGLRGEFGNPGRDGARGAPGAVGSPGPSGATGDRGEAGAAGP

AGPAGPRGSPGERGEVGPAGPNGFAGPPGAAGQAGAKGERGTKGPKGENGPTGPVGAVGS

AGPAGPNGLPGPTGsRGDGGPPGMTGFPGAAGRTGPAGPSGITGPSGPPGASGKEGPRGP

RGDQGPVGRTGELGAVGPPGFTGEKGPSGEPGTAGPPGTPGPQGLLGSPGILGLPGSRGE

RGLPGVSGGLGEPGPLGLSGPSGARGPPGNVGNPGVNGAPGEAGRDGNPGSDGPPGRDGA

AGVKGDRGypGPVGAPGAPGAPGSPGPVGPTGKXGnRGEPGPAGxxGxxGxxGPRgXSGP

QGPRGEKGETGDKGHRGMNGFKGHNGLQGLPGLAGPSGDQGTSGPAGPSGPRGPLGPHGP

PGKDGVSGHPGPIGPPGIRGSQGSQGPAGPVGPVGSLGPAGPSGGGYDFGYEGDFYRA

>Macropus

QMSYGYDEKSGG-ISVPGPMGPSGPRGLPGPPGXPGPQGFQGPPGEPGEPGASGPMGPRG

PXGPPGKNGDDGEAGKPGRPGERGPPGPQGARGLPGTAGLPGMKGHRGFSGLDGAKGDSG

PAGPKGEPGSPGENGAPGQMGPRGLPGERGRPGPPGPAGARGNDGATGAAGPPGPTGPAG

PPGFPGAVGAKGEAGPQGARGSEGPQGVRGEPGPPGPAGAAGPSGNPGADGQPGAKGANG

APGIAGAPGFPGARGPSGPQGPSGAPGPKGNSGEPGAPGNKGDAGAKGEPGPVGVQGPPG

PAGEEGKRGSRGEPGPTGLPGPAGERGGPGSRGFPGADGVAGPKGAPGERGAPGPAGPKG

SPGESGRPGEAGLPGAKGLTGSPGSPGPDGKTGPPGPAGQDGRPGPPGPPGARGQAGVMG

FPGPKGAAGEPGKAGERGVPGPPGAVGAAGKDGEAGAQGPPGPAGPAGERGEQGPAGSPG

FQGLPGPAGPPGEAGKPGEQGVPGDAGAPGPSGARGERGFPGERGVQGPPGPQGPRGANG

APGNDGAKGDAGAPGAPGSQGPPGLQGMPGERGAAGLPGAKGDRGDAGPKGADGAPGKDG

VRGLTGPIGPPGPAGPSGDKGESGPSGPVGPTGARGAPGERGEPGPPGPAGFAGPPGADG

QPGAKGEPGDAGAKGDAGPPGPAGPTGAPGPAGNVGAPGPKGARGSAGPPGATGFPGAAG

RVGPPGPSGNTGPPGPPGPAGKEGGKGPRGETGPVGRPGEVGPPGPPGPSGEKGSPGADG

PAXXXXXXXXXXXXXXXXXXXXXXXXXXXXXXXXXXXXXXXXXXXXXXXXXXXXXXXXXX

XXXXXXXXXXXXXXXXXXXXXXXXXXXXXXXXXXXXXXXXXXXXXXXXXXXXXXXXXXXX

XXXXXXXXXXXXXXXXXXXXXXXXXXXXXXXXXXXXXXXXXXXXXXXXXXXXXXXXXXXX

XXXXXXXXXXXXXXXXXXXXXXXXXXXXXXXXXXXXXXXXXXXXXXXXXXXXXXXXGPPG

PPGPPGPPGPPSGGFDFSFLPQPPQEKAHDSGRYYRARXXXxXXXXXXXXXXGLMGPRGP

xGASGPPGAQGFQGPAGEPGEPGQTGPAGARGPPGPPGKSGEDGHPGKPGRPGERGIVGP

QGARGFPGTPGLPGFKXXXXXXXXXXXXXXXXXXXXXGEPGAPGENGTPGQAGARGLPGE

RGRIGGAGPAGARGSDGSVGPVGPAGPIGSAGPPGFPGAPGPKGELGPVGNPGPAGPAGP

RGELGLPGMTGPVGPAGNPGANGLTGAKGAAGLPGVAGAPGLPGPRGIPGPAGAAGASGP

RGLAGEPGPAGAKGESGNKGEPGAAGPQGPPGPSGEEGKRGPNGEPGSTGPTGPPGLRGV

PGSRGLPGADGRAGGMGPPGNRGSSGPAGARGPNGDAGRPGEPGLMGPRGLPGSPGNPGP

TGKEGPAGLPGPDGRPGPTGPAGNRGEPGNIGFPGPKGPNGEPGKSGEKGHAGLAGARGA

PGPDGNNGAQGPPGPAGVQGGKGEQGPAGPPGFQGLPGPSGPAGEGGKVGERGIPGEFGL

PGPAGPRXXXXXXXXXXXXXXXXXXXXXXXXXXXXXXXXXGEPGVVGAPGSAGPAGSGGV

PGERGAAGVPGGKGEKGETGLRGDFGNPGRDGARGAPGAMGAPGPAGATGERGEAGPAGP

VGPTGARGAPGDRGEAGPAGPNGFAGPPGAAGQAGAKGERGTKGPKGENGVVGPTGPVGA

AGPAGPNGPPGPVGGRGDGGPPGATGFPGAAGRTGAPGPAGITGPPGPPGASGKEGPRGP

RGDQGPLGRAGETGAVGPPGFAGEKGPPGEAGATGPPGSSGPQGLLGAPGILGLPGSRGE

RGLPGVSGALGEPGPLGLAGPPGARGPPGAVGNPGVNGAPGEAGRDGNPGNDGPPGRDGL

AGHKGERGYPGNAGAVGNAGAPGPHGTVGPAGKPGNRGEPGPVGSVGPAGPFGARGPSGP

QGPRGDKGEVGDKGPRGLNGLKGHNGFQGLPGlAGQHGDQGAPGSIGPAGPRGPAGPSGP

AGKDGRPGQAGAVGPAGLRGSQGSQGPAGPPGPPGLPGPPGPSGGGYDFGYDGDFYRA

>Loxodonta

QLSYGYDEKSAGGISVPGPMGPSGPRGLPGPPGAPGPQGFQGPPGEPGEPGASGPMGPRG

PPGPPGKNGDDGEAGKPGRPGERGPPGPQGARGLPGTAGLPGMKGHRGFSGLDGAKGDAG

PAGPKGEPGSPGENGAPGQMGPRGLPGERGRPGAPGPAGARGNDGATGAAGPPGPTGPAG

PPGFPGAVGAKGEAGPQGARGSEGPQGVRGEPGPPGPAGAAGPAGNPGADGQPGAKGANG

APGIAGAPGFPGARGPAGPQGPSGAPGPKGNSGEPGAPGSKGDAGAKGEPGPVGIQGPPG

PAGEEGKRGARGEPGPTGLPGPPGERGGPGSRGFPGADGVAGPKGPAGERGSPGPAGPKG

SPGEAGRPGEAGLPGAKGLTGSPGSPGPDGKTGPPGPAGQDGRPGPPGPPGARGQAGVMG

FPGPKGAAGEPGKAGERGVPGPPGAVGAAGKDGEAGAQGPPGPAGPAGERGEQGPAGSPG

FQGLPGPAGPPGEAGKPGEQGVPGDLGAPGPSGARGERGFPGERGVQGPPGPAGPRGSNG

APGNDGAKGDAGAPGAPGSQGAPGLQGMPGERGAAGLPGPKGDRGDAGPKGADGSPGKDG

PRGLTGPIGPPGPAGAPGDKGEAGPSGPAGPTGARGAPGDRGEPGPPGPAGFAGPPGADG

QPGAKGEPGDAGAKGDAGPPGPAGPTGAPGPIGNVGAPGPKGARGSAGPPGATGFPGAAG

RVGPPGPSGNAGPPGPPGPAGKEGGKGPRGETGPAGRPGEVGPPGPPGPAGEKGSPGADG

PAGAPGTPGPQGIGGQRGVVGLPGQRGERGFPGLPGPSGEPGKQGPSGSSGERGPPGPAG

PPGLAGPPGESGREGAPGAEGSPGRDGSPGPKGDRGETGPSGPPGAPGAPGAPGPVGPAG

KSGDRGETGPAGPAGPAGPAGVRGPAGPQGPRGDKGETGEQGDRGiKGHRGFSGLQGPPG

PPGSPGEQGPSGASGPAGPRGPPGSAGAPGKDGLNGLPGPIGPPGPRGRTGDAGPVGPPG

PPGPPGPPGPPSGAFDFSFLPQPPQEKAHDGGRYYRARQYDAKGIGLGPGPMGLMGPRGP

PGATGPPGSPGFQGPPGEPGEPGQTGPAGSRGPAGPPGKAGEDGHPGKPGRPGERGVVGP

QGARGFPGTPGLPGFKGIRGHNGLDGLKGQPGAPGVKGEPGAPGENGTPGQIGARGLPGE

RGRVGGPGPAGARGSDGSVGPVGPAGPIGSAGPPGFPGAPGPKGELGPVGNPGPSGPAGP

RGEAGLPGVSGPVGPPGNPGANGLAGAKGAAGLPGVAGAPGLPGPRGIPGPVGAAGATGA

RGLVGEPGPAGSKGESGSKGEPGSAGPQGPPGPSGEEGKRGSSGEAGSAGPAGPPGLRGG

PGSRGLPGADGRAGVMGPPGSRGASGPAGVRGPSGDSGRPGEPGVMGPRGLPGSPGNVGP

AGKEGPAGLPGIDGRPGPIGPAGARGEPGNIGFPGPKGPAGDPGKNGDKGHAGLAGPRGA

PGPDGNNGAQGPPGLQGVQGGKGEQGPAGPPGFQGLPGPSGTAGEAGKPGERGIPGEFGL

PGPAGPRGERGPPGQSGAAGPTGPIGSRGPSGPPGPDGNKGEPGVVGAPGTAGPSGPGGL

PGERGAAGIPGGKGEKGETGLRGDTGNTGRDGARGAPGAVGAPGPAGATGDRGEAGPAGS

AGPAGPRGSPGERGEVGPAGPNGFAGPAGAAGQAGAKGERGTKGPKGENGPVGPTGPVGA

AGPAGPNGPPGPAGSRGDGGPPGATGFPGAAGRTGPPGPAGITGPPGPPGAAGKEGLRGP

RGDQGPVGRTGETGASGPPGFAGEKGSSGEPGTAGPPGTPGPQGILGPPGILGLPGSRGE

RGLPGVAGAVGEPGPLGIAGPPGARGPPGAVGSPGVNGAPGEAGRDGNPGSDGPPGRDGL

PGHKGERGYPGNAGPVGTAGAPGPQGPLGPAGKHGNRGEPGPAGSVGPVGAVGPRGPSGP

QGARGDKGEAGDKGPRGLPGFKGHNGLQGLPGLAGQHGDQGSPGSVGPAGPRGPAGPSGP

VGKDGRPGHAGAVGPAGVRGSQGSQGPSGPPGPPGPPGPPGPSGGGYDFGYDGDFYRA

>Canis

QMSYGYDEKSTGGISVPGPMGPSGPRGLPGPPGAPGPQGFQGPPGEPGEPGASGPMGPRG

PPGPPGKNGDDGEAGKPGRPGERGPPGPQGARGLPGTAGLPGMKGHRGFSGLDGAKGDAG

PAGPKGEPGSPGENGAPGQMGPRGLPGERGRPGAPGPAGARGNDGATGAAGPPGPTGPAG

PPGFPGAVGAKGEAGPQGARGSEGPQGVRGEPGPPGPAGAAGPAGNPGADGQPGAKGANG

APGIAGAPGFPGARGPSGPQGPSGPPGPKGNSGEPGAPGNKGDTGAKGEPGPTGIQGPPG

PAGEEGKRGARGEPGPTGLPGPPGERGGPGSRGFPGADGVAGPKGPAGERGSPGPAGPKG

SPGEAGRPGEAGLPGAKGLTGSPGSPGPDGKTGPPGPAGQDGRPGPPGPPGARGQAGVMG

FPGPKGAAGEPGKAGERGVPGPPGAVGPAGKDGEAGAQGPPGPAGPAGERGEQGPAGSPG

FQGLPGPAGPPGEAGKPGEQGVPGDLGAPGPSGARGERGFPGERGVQGPPGPAGPRGANG

APGNDGAKGDAGAPGAPGSQGAPGLQGMPGERGAAGLPGPKGDRGDAGPKGADGSPGKDG

VRGLTGPIGPPGPAGAPGDKGEAGPSGPAGPTGARGAPGDRGEPGPPGPAGFAGPPGADG

QPGAKGEPGDAGAKGDAGPPGPAGPTGPPGPIGNVGAPGPKGARGSAGPPGATGFPGAAG

RVGPPGPSGNAGPPGPPGPAGKEGGKGARGETGPAGRPGEVGPPGPPGPAGEKGSPGADG

PAGAPGTPGPQGIAGQRGVVGLPGQRGERGFPGLPGPSGEPGKQGPSGTSGERGPPGPMG

PPGLAGPPGESGREGAPGAEGSPGRDGSPGPKGDRGETGPAGPPGAPGAPGAPGPVGPAG

KNGDRGETGPAGPAGPIGPVGARGPAGPQGPRGDKGETGEQGDRGIKGHRGFSGLQGPPG

PPGSPGEQGPSGASGPAGPRGPPGSAGSPGKDGLNGLPGPIGPPGPRGRTGDAGPVGPPG

PPGPPGPPGPPSGGFDFSFLPQPPQEKAHDGGRYYRARQYDGKGVGLGPGPMGLMGPRGP

PGASGAPGPQGFQGPAGEPGEPGQTGPAGARGPPGPPGKAGEDGHPGKPGRPGERGVVGP

QGARGFPGTPGLPGFKGIRGHNGLDGLKGQPGAPGVKGEPGAPGENGTPGQTGARGLPGE

RGRVGAPGPAGARGSDGSVGPVGPAGPIGSAGPPGFPGAPGPKGELGPVGNPGPAGPAGP

RGEVGLPGVSGPVGPPGNPGANGLTGAKGAAGLPGVAGAPGLPGPRGIPGPVGAAGATGA

RGlVGEPGPAGSKGESGNKGEPGSAGAQGPPGPSGEEGKRGPNGEAGSAGPSGPPGLRGS

PGSRGLPGADGPAGVMGPPGPRGATGPAGVRGPNGDSGRPGEPGLMGPRGFPGAPGNVGP

AGKEGPMGLPGIDGRPGPIGPAGARGEPGNIGFPGPKGPTGDPGKNGDKGHAGLAGARGA

PGPDGNNGAQGPPGPQGVQGGKGEQGPAGPPGFQGLPGPAGTAGEVGKPGERGLPGEFGL

PGPAGPRGERGPPGESGAAGPSGPIGSRGPSGPPGPDGNKGEPGVLGAPGTAGASGPGGL

PGERGAAGIPGGKGEKGETGLRGEIGNPGRDGARGAPGAMGAPGPAGATGDRGEAGPAGP

AGPAGPRGTPGERGEVGPAGPNGFAGPAGAAGQPGAKGERGTKGPKGENGPVGPTGPIGS

AGPSGPNGPPGPAGSRGDGGPPGATGFPGAAGRTGPPGPSGITGPPGPPGAAGKEGLRGP

RGDQGPVGRTGETGASGPPGFTGEKGPSGEPGTAGPPGTPGPQGLLGAPGILGLPGSRGE

RGLPGVAGSVGEPGPLGIAGPPGARGPPGAVGAPGVNGAPGEAGRDGNPGNDGPPGRDGQ

AGHKGERGYPGNIGPVGAVGAPGPHGPVGPTGKHGNRGEPGPAGSVGPVGAVGPRGPSGP

QGIRGDKGEPGEKGPRGLPGLKGHNGLQGLPGLAGQHGDQGAPGSVGPAGPRGPAGPSGP

AGKDGRTGQPGTVGPAGIRGSQGSQGPAGPPGPPGPPGPPGPSGGGYDFGYEGDFYRA

>Pan

QLSYGYDEKSTGGISVPGPMGPSGPRGLPGPPGAPGPQGFQGPPGEPGEPGASGPMGPRG

PPGPPGKNGDDGEAGKPGRPGERGPPGPQGARGLPGTAGLPGMKGHRGFSGLDGAKGDAG

PAGPKGEPGSPGENGAPGQMGPRGLPGERGRPGAPGPAGARGNDGATGAAGPPGPTGPAG

PPGFPGAVGAKGEAGPQGPRGSEGPQGVRGEPGPPGPAGAAGPAGNPGADGQPGAKGANG

APGIAGAPGFPGARGPSGPQGPGGPPGPKGNSGEPGAPGSKGDTGAKGEPGPVGVQGPPG

PAGEEGKRGARGEPGPTGLPGPPGERGGPGSRGFPGADGVAGPKGPAGERGSPGPAGPKG

SPGEAGRPGEAGLPGAKGLTGSPGSPGPDGKTGPPGPAGQDGRPGPPGPPGARGQAGVMG

FPGPKGAAGEPGKAGERGVPGPPGAVGPAGKDGEAGAQGPPGPAGPAGERGEQGPAGSPG

FQGLPGPAGPPGEAGKPGEQGVPGDLGAPGPSGARGERGFPGERGVQGPPGPAGPRGANG

APGNDGAKGDAGAPGAPGSQGAPGLQGMPGERGAAGLPGPKGDRGDAGPKGADGSPGKDG

VRGLTGPIGPPGPAGAPGDKGESGPSGPAGPTGARGAPGDRGEPGPPGPAGFAGPPGADG

QPGAKGEPGDAGAKGDAGPPGPAGPAGPPGPIGNVGAPGAKGARGSAGPPGATGFPGAAG

RVGPPGPSGNAGPPGPPGPAGKEGGKGPRGETGPAGRPGEVGPPGPPGPAGEKGSPGADG

PAGAPGTPGPQGIAGQRGVVGLPGQRGERGFPGLPGPSGEPGKQGPSGASGERGPPGPMG

PPGLAGPPGESGREGAPGAEGSPGRDGSPGAKGDRGETGPAGPPGAPGAPGAPGPVGPAG

KSGDRGETGPAGPAGPVGPVGARGPAGPQGPRGDKGETGEQGDRGIKGHRGFSGLQGPPG

PPGSPGEQGPSGASGPAGPRGPPGSAGAPGKDGLNGLPGPIGPPGPRGRTGDAGPVGPPG

PPGPPGPPGPPSAGFDFSFLPQPPQEKAHDGGRYYRARQYDGKGVGLGPGPMGLMGPRGP

PGAAGAPGPQGFQGPAGEPGEPGQTGPAGARGPAGPPGKAGEDGHPGKPGRPGERGVVGP

QGARGFPGTPGLPGFKGIRGHNGLDGLKGQPGAPGVKGEPGAPGENGTPGQTGARGLPGE

RGRVGAPGPAGARGSDGSVGPVGPAGPIGSAGPPGFPGAPGPKGELGAVGNAGPAGPAGP

RGEVGLPGLSGPVGPPGNPGANGLTGAKGAAGLPGVAGAPGLPGPRGIPGPVGAAGATGA

RGLVGEPGPAGSKGESGNKGEPGSAGPQGPPGPSGEEGKRGPNGEAGSAGPPGPPGLRGS

PGSRGLPGADGRAGVMGPAGSRGASGPAGVRGPNGDAGRPGEPGLMGPRGLPGSPGNIGP

AGKEGPVGLPGIDGRPGPIGPAGARGEPGNIGFPGPKGPTGDPGKNGDKGHAGLAGARGA

PGPDGNNGAQGPPGPQGVQGGKGEQGPAGPPGFQGLPGPSGPTGEVGKPGERGLHGEFGL

PGPAGPRGERGPPGESGAAGPTGPIGSRGPSGPPGPDGNKGEPGVVGAVGTAGPSGPSGL

PGERGAAGIPGGKGEKGEPGLRGEIGNPGRDGARGAPGAVGAPGPAGATGDRGEAGAAGP

AGPAGPRGSPGERGEVGPAGPNGFAGPAGAAGQPGAKGERGAKGPKGENGVVGPTGPVGA

AGPAGPNGPPGPAGSRGDGGPPGMTGFPGAAGRTGPPGPSGISGPPGPPGPAGKEGLRGP

RGDQGPVGRTGEVGAVGPPGFAGEKGPSGEAGTAGPPGTPGPQGLLGAPGILGLPGSRGE

RGLPGVAGAVGEPGPLGIAGPPGARGPPGAVGSPGVNGAPGEAGRDGNPGNDGPPGRDGQ

PGHKGERGYPGNIGPVGAAGAPGPHGPVGPAGKHGNRGETGPSGPVGPAGAVGPRGPSGP

QGIRGDKGEPGEKGPRGLPGLKGHNGLQGLPGlAGHHGDQGAPGSVGPAGPRGPAGPSGP

AGKDGRTGHPGTVGPAGIRGPQGHQGPAGPPGPPGPPGPPGVSGGGYDFGYDGDFYRA

>Nomascus

QLSYGYDEKSAGGISVPGPMGPSGPRGLPGPPGAPGPQGFQGPPGEPGEPGASGPMGPRG

PPGPPGKNGDDGEAGKPGRPGERGPPGPQGARGLPGTAGLPGMKGHRGFSGLDGAKGDAG

PAGPKGEPGSPGENGAPGQMGPRGLPGERGRPGAPGPAGARGNDGATGAAGPPGPTGPAG

PPGFPGAVGAKGEAGPQGPRGSEGPQGVRGEPGPPGPAGAAGPAGNPGADGQPGAKGANG

APGIAGAPGFPGARGPSGPQGPGGPPGPKGNSGEPGAPGSKGDTGAKGEPGPVGVQGPPG

PAGEEGKRGARGEPGPTGLPGPPGERGGPGSRGFPGADGVAGPKGPAGERGSPGPAGPKG

SPGEAGRPGEAGLPGAKGLTGSPGSPGPDGKTGPPGPAGQDGRPGPPGPPGARGQAGVMG

FPGPKGAAGEPGKAGERGVPGPPGAVGPAGKDGEAGAQGPPGPAGPAGERGEQGPAGSPG

FQGLPGPAGPPGEAGKPGEQGVPGDLGAPGPSGARGERGFPGERGVQGPPGPAGPRGANG

APGNDGAKGDAGAPGAPGSQGAPGLQGMPGERGAAGLPGPKGDRGDAGPKGADGSPGKDG

VRGLTGPIGPPGPAGAPGDKGETGPSGPAGPTGARGAPGDRGEPGPPGPAGFAGPPGADG

QPGAKGEPGDAGAKGDAGPPGPAGPAGPPGPIGNVGAPGAKGARGSAGPPGATGFPGAAG

RVGPPGPSGNAGPPGPPGPAGKEGGKGPRGETGPAGRPGEVGPPGPPGPAGEKGSPGADG

PAGAPGTPGPQGIAGQRGVVGLPGQRGERGFPGLPGPSGEPGKQGPSGASGERGPPGPMG

PPGLAGPPGESGREGAPGAEGSPGRDGSPGPKGDRGETGPAGPPGAPGAPGAPGPVGPAG

KSGDRGETGPAGPAGPVGPVGARGPAGPQGPRGDKGETGEQGDRGIKGHRGFSGLQGPPG

PPGSPGEQGPSGASGPAGPRGPPGSAGAPGKDGLNGLPGPIGPPGPRGRTGDAGPVGPPG

PPGPPGPPGPPSAGFDFSFLPQPPQEKAHDGGRYYRARQYDGKGVGLGPGPMGLMGPRGP

PGAAGAPGPQGFQGPAGEPGEPGQTGPAGARGPAGPPGKAGEDGHPGKPGRPGERGVVGP

QGARGFPGTPGLPGFKGIRGHNGLDGLKGQPGAPGVKGEPGAPGENGTPGQTGARGLPGE

RGRVGAPGPAGARGSDGSVGPVGPAGPIGSAGPPGFPGAPGPKGELGAVGNAGPAGPAGP

RGEVGLPGLSGPVGPPGNPGANGLTGAKGAAGLPGVAGAPGLPGPRGIPGPVGAAGATGA

RGLVGEPGPAGSKGESGNKGEPGSAGPQGPPGPSGEEGKRGPNGEAGSAGPPGPPGLRGS

PGSRGLPGADGRAGVMGPPGSRGASGPAGVRGPNGDAGRPGEPGLMGPRGLPGSPGNIGP

AGKEGPVGLPGIDGRPGPIGPAGARGEAGNIGFPGPKGPTGDPGKSGDKGHAGLAGARGA

PGPDGNNGAQGPPGPQGVQGGKGEQGPAGPPGFQGLPGPSGPAGEVGKPGERGLHGEFGL

PGPAGPRGERGPPGESGAAGPTGPIGSRGPSGPPGPDGNKGEPGVVGAVGTAGPSGPSGL

PGERGAAGIPGGKGEKGEPGLRGEIGNPGRDGARGAPGAVGAPGPAGATGDRGEAGAAGP

AGPAGPRGSPGERGEVGPAGPNGFAGPAGAAGQPGAKGERGAKGPKGENGVVGPTGPVGA

AGPAGPNGPPGPAGSRGDGGPPGMTGFPGAAGRTGPPGPSGISGPPGPPGPAGKEGLRGP

RGDQGPVGRTGEVGAVGPPGFAGEKGPSGEAGTAGPPGTPGPQGLLGAPGILGLPGSRGE

RGLPGVAGAVGEPGPLGIAGPPGARGPPGAVGSPGVNGAPGEAGRDGNPGNDGPPGRDGQ

PGHKGERGYPGNIGPVGAAGAPGPHGPVGPAGKHGNRGETGPSGPVGPAGAVGPRGPSGP

QGIRGDKGEPGDKGPRGLPGLKGHNGLQGLPGlAGHHGDQGAPGSVGPAGPRGPAGPSGP

AGKDGRTGHPGTVGPAGIRGPQGHQGPAGPPGPPGPPGPPGVSGGGYDFGYDGDFYRA

>Macaca

QLSYGYDEKSTGGISVPGPMGPSGPRGLPGPPGAPGPQGFQGPPGEPGEPGASGPMGPRG

PPGPPGKNGDDGEAGKPGRPGERGPPGPQGARGLPGTAGLPGMKGHRGFSGLDGAKGDAG

PAGPKGEPGSPGENGAPGQMGPRGLPGERGRPGAPGPAGARGNDGATGAAGPPGPTGPAG

PPGFPGAVGAKGEAGPQGPRGSEGPQGVRGEPGPPGPAGAAGPAGNPGADGQPGAKGANG

APGIAGAPGFPGARGPSGPQGPGGPPGPKGNSGEPGAPGSKGDTGAKGEPGPVGVQGPPG

PAGEEGKRGARGEPGPTGLPGPPGERGGPGSRGFPGADGVAGPKGPAGERGSPGPAGPKG

SPGEAGRPGEAGLPGAKGLTGSPGSPGPDGKTGPPGPAGQDGRPGPPGPPGARGQAGVMG

FPGPKGAAGEPGKAGERGVPGPPGAVGPAGKDGEAGAQGPPGPAGPAGERGEQGPAGSPG

FQGLPGPAGPPGEAGKPGEQGVPGDLGAPGPSGARGERGFPGERGVQGPPGPAGPRGANG

APGNDGAKGDAGAPGAPGSQGAPGLQGMPGERGAAGLPGPKGDRGDAGPKGADGSPGKDG

VRGLTGPIGPPGPAGAPGDKGETGPSGPAGPTGARGAPGDRGEPGPPGPAGFAGPPGADG

QPGAKGEPGDAGAKGDAGPPGPAGPAGPPGPIGNVGAPGPKGARGSAGPPGATGFPGAAG

RVGPPGPSGNAGPPGPPGPAGKEGGKGPRGETGPAGRPGEVGPPGPPGPAGEKGSPGADG

PAGAPGTPGPQGIAGQRGVVGLPGQRGERGFPGLPGPSGEPGKQGPSGASGERGPPGPMG

PPGLAGPPGESGREGAPGAEGSPGRDGSPGAKGDRGETGPAGPPGAPGAPGAPGPVGPAG

KSGDRGETGPAGPAGPVGPVGARGPAGPQGPRGDKGETGEQGDRGIKGHRGFSGLQGPPG

PPGSPGEQGPSGASGPAGPRGPPGSAGTPGKDGLNGLPGPIGPPGPRGRTGDAGPVGPPG

PPGPPGPPGPPSGGFDFSFLPQPPQEKAHDGGRYYRARQYDGKGVGLGPGPMGLMGPRGP

PGAAGAPGPQGFQGPAGEPGEPGQTGPAGSRGPAGPPGKAGEDGHPGKPGRPGERGVVGP

QGARGFPGTPGLPGFKGIRGHNGLDGLKGQPGAPGVKGEPGAPGENGTPGQTGARGLPGE

RGRVGAPGPAGARGSDGSVGPVGPAGPIGSAGPPGFPGAPGPKGELGAVGNAGPAGPAGP

RGEVGLPGLSGPVGPPGNPGANGLTGAKGAAGLPGVAGAPGLPGPRGIPGPVGAAGATGA

RGLVGEPGPAGSKGESGNKGEPGSAGPQGPPGPSGEEGKRGPNGEVGSAGPPGPPGLRGS

PGSRGLPGADGRAGVMGPPGSRGASGPAGVRGPNGDAGRPGEPGLMGPRGLPGSPGNIGP

AGKEGPVGLPGIDGRPGPIGPAGARGEPGNIGFPGPKGPTGDPGKNGDKGHAGLAGARGA

PGPDGNNGAQGPPGPQGVQGGKGEQGPAGPPGFQGLPGPSGPAGEVGKPGERGLPGEFGL

PGPAGARGERGPPGESGAAGPTGPIGSRGPSGPPGPDGNKGEPGVVGAAGTAGPSGPSGL

PGERGAAGIPGGKGEKGEPGLRGEIGNPGRDGARGAPGAVGAPGPAGATGDRGEAGAAGP

AGPAGPRGSPGERGEVGPAGPNGFAGPAGAAGQPGAKGERGAKGPKGENGVVGPTGPVGA

AGPSGPNGPPGPAGSRGDGGPPGMTGFPGAAGRTGPPGPSGISGPPGPPGPSGKEGLRGP

RGDQGPVGRTGEVGAVGPPGFAGEKGPSGEAGTAGPPGTPGPQGLLGAPGILGLPGSRGE

RGLPGVAGVVGEPGPLGIAGPPGARGPPGAVGSPGVNGAPGEAGRDGNPGNDGPPGRDGQ

PGHKGERGYPGNNGPVGAAGAPGPHGPVGPAGKHGNRGETGPSGPVGPAGAVGPRGPSGP

QGIRGDKGEPGDKGPRGLPGLKGHNGLQGLPGLAGHHGDQGAPGSVGPAGPRGPAGPSGP

AGKDGRTGHPGTVGPAGIRGPQGHQGPAGPPGPPGPPGPPGVSGGGYDFGYDGDFYRA

>Otolemur

QMSYGYDEKSAGXISVPGPMGPSGPRGLPGPPGAPGPQGFQGPPGEPGEPGASGPMGPRG

PPGPPGKNGDDGEAGKPGRPGERGPPGPQGARGLPGTAGLPGMKGHRGFSGLDGAKGDAG

APGPKGEPGSPGENGAPGQMGPRGLPGERGRPGPxGPAGARGNDGATGAAGPPGPTGPAG

PPGFPGAAGAKGEAGPQGXRGSEGPQGVRGEPGPPGPAGAAGPAGNPGADGQPGAKGANG

APGIAGAPGFPGARGPSGPQGPGGXPGPKGNSGEPGAPGXKGDTGAKGEPGPVGVQGPPG

PAGEEGKRGARGEPGPXGLPGPPGERGGPGSRGFPGADGVAGPKGPAGERGSPGPAGPKG

SPGEAGRPGEAGLPGAKGLTGSPGSPGPDGKTGPPGPAGQDGRPGPPGPPGARGQAGVMG

FPGPKGAAGEPGKAGERGVPGPTGAVGPAGKDGEAGAQGPPGPAGPAGERGEQGPAGSPG

FQGLPGPAGPPGEAGKPGEQGVPGDLGAPGPSGARGERGFPGERGVQGPPGPAGPRGSNG

APGNDGAKGDAGAPGAPGSQGAPGLQGMPGERGAAGLPGXKGDRGDAGPKGADGXPGKDG

XRGLTGPIGPPGPAGAPGDKGEXGPSGPAGPTGARGAPGDRGEXGPPGPAGFAGPPGADG

QPGAKGEPGDAGXKGDAGPPGPAGPXGPPGPIGNVGAPGPKGARGSAGPPGATGFPGAAG

RVGPPGPSGNAGPPGPPGPAGKEGGKGPRGETGPAGRPGEVGPPGPPGPAGEKGSPGADG

PAGAPGTPGPQGIXGQRGXVGLPGQRGERGFPGLPGPSGEPGKQGPSGXSGERGPPGPMG

PPGLAGPPGEXGREGXPGAEGSPGRDGSPGPKGDRGETGPAGPPGAPGAPGAPGPVGPAG

KNGDRGETGPAGPAGPXGPVGXRGPXGPQGPRGDKGETGEQGDRGiKGHRGFSGLQGPPG

PPGSPGEQGPSGASGPAGPRGPXGSAGXPGKDGLNGLPGPIGPPGPRGRTGDAGPVGPPG

PPGPPGPPGPPSGGXDFSFLPQPPQEKXHDGGRYYRARQYDGKAAGLGPGPMGLMGPRGP

PGASGAPGPQGFQGPAGEPGEPGQTGPAGARGPAGAPGKAGEDGHPGKPGRPGERGVVGP

QGARGFPGTPGLPGFKGLRGHSGPDGLKGQAGLPGAKGEPGSPGENGTPGQTGARGLPGE

RGRVGAPGPSGARGSDGSVGPVGPAGPVGSAGPPGFPGAPGPKGELGPVGNPGPAGPAGP

RGEVGLPGLSGPVGPPGNPGANGLTGAKGAAGLPGVAGAPGLPGPRGIPGPVGAAGATGA

RGLVGEPGPAGSKGESGNKGEPGSAGPQGPPGPSGEEGKRGSNGEPGSAGPSGPPGLRGS

PGSRGLPGADGRxGVMGPPGNRGQSGPAGVRGPSGDSGRPGEPGLMGPRGLPGSPGNVGP

AGKEGPAGLPGVDGRPGPVGPAGARGEPGNIGFPGPKGPSGDPGKAGDKGHPGLAGARGA

PGPDGNNGAQGPPGPQGVQGGKGEQGPAGPPGFQGLPGPSGPAGEVGKPGERGLXGEFGL

PGPAGXRGERGPPGESGAAGPSGPLGSRGPSGPPGPDGNKGEPGVVGAPGTAGPSGPSGL

PGERGAAGMPGGKGEKGETGLRGEXGTTGRDGARGAPGAVGAPGPAGATGDRGEAGAAGP

AGPAGPRGSPGERGEVGPAGPNGFAGPAGAAGQPGAKGERGAKGPKGENGAVGPAGAVGP

AGPSGPNGPPGPAGSRGDGGPPGMTGFPGAAGRTGPPGPSGISGPPGPPGPAGKEGLRGP

RGDQGPVGRTGEXGAVGPPGFAGEKGPSGEAGTAGPPGXXGPQGlLGXPGXLGLPGSRGE

RGLPGXAGXXGEPGPLGVAGPPGARGPSGGVGNPGVNGAPGEAGRDGNPGNDGPPGRDGQ

PGHKGERGYPGNVGPAGAVGAPGSHGPVGPAGKHGNRGEPGAVGPVGPTGAVGPRGPSGA

QGVRGDKGEPGDKGPRGLPGLKGHxGLQGLPGlAGHHGDQGAPGSVGPAGPRGPAGPSGP

VGKDGRNGHPGTVGPAGVRGPQGHQGPAGPPGPPGPPGPPGASGGGYDFGYDGDFYRA

>Callithrix

QLSYGYDEKSTGGISVPGPMGPSGPRGLPGPPGSPGPQGFQGPPGEPGEPGASGPMGPRG

PPGPPGKNGDDGEAGKPGRPGERGPPGPQGARGLPGTAGLPGMKGHRGFSGLDGAKGDAG

PAGPKGEPGSPGENGAPGQMGPRGLPGERGRPGPPGPAGARGNDGATGAAGPPGPTGPAG

PAGFPGAVGAKGEAGPQGPRGSEGPQGVRGEPGPPGPAGAAGPAGNPGADGQPGAKGANG

APGIAGAPGFPGARGPSGPQGPSGPPGPKGNSGEPGAPGSKGDTGAKGEPGPVGVQGPPG

PAGEEGKRGARGEPGPTGLPGPPGERGGPGSRGFPGADGVAGPKGPAGERGSPGPAGPKG

SPGEAGRPGEAGLPGAKGLTGSPGSPGPDGKTGPPGPAGQDGRPGPPGPPGARGQAGVMG

FPGPKGAAGEPGKAGERGVPGPPGAVGPAGKDGEAGAQGPPGPAGPAGERGEQGPAGSPG

FQGLPGPAGPPGEAGKPGEQGVPGDLGAPGPSGARGERGFPGERGVQGPPGPAGPRGANG

APGNDGAKGDAGAPGAPGSQGAPGLQGMPGERGAAGLPGPKGDRGDAGPKGADGSPGKDG

VRGLTGPIGPPGPAGAPGDKGETGPSGPAGPTGARGAPGDRGEPGPPGPAGFAGPPGADG

QPGAKGEPGDAGAKGDAGPPGPAGPAGPPGPIGNVGAPGPKGARGSAGPPGATGFPGAAG

RVGPPGPSGNAGPPGPPGPAGKEGGKGPRGETGPAGRPGEVGPPGPPGPAGEKGSPGADG

PAGAPGTPGPQGIAGQRGVVGLPGQRGERGFPGLPGPSGEPGKQGPSGTSGERGPPGPMG

PPGLAGPPGESGREGAPGAEGSPGRDGSPGPKGDRGETGPAGPPGAPGAPGAPGPVGPAG

KSGDRGETGPAGPAGPIGPVGSRGPAGPQGPRGDKGETGEQGDRGIKGHRGFSGLQGPPG

PPGSPGEQGPSGASGPAGPRGPPGSAGAPGKDGLNGLPGPIGPPGPRGRTGDAGPVGPPG

PPGPPGPPGPPSGGLDFSFLPQPPQEKAHDGGRYYRARQYDGKGVGLGPGPMGLMGPRGP

PGAAGAPGPQGFQGPAGEPGEPGQTGPAGARGPPGPPGKAGEDGHPGKPGRPGERGVVGP

QGARGFPGTPGLPGFKGIRGHNGLDGLKGQPGAPGVKGEPGAPGENGTPGQTGARGLPGE

RGRVGAPGPAGARGSDGSVGPVGPAGPIGSAGPPGFPGAPGPKGELGAIGNPGIAGPAGP

RGEVGLPGLSGPVGPPGNPGANGLTGAKGAAGLPGVAGAPGLPGPRGIPGPVGAAGATGA

RGLVGEPGPAGSKGESGNKGEPGSAGPQGPPGPSGEEGKRGPNGEAGSAGPPGPPGLRGS

PGSRGLPGADGRAGVMGPAGSRGATGPAGVRGPNGDAGRPGEPGLMGPRGLPGSPGNIGP

AGKEGPVGLPGIDGRPGPIGPAGARGEPGSIGFPGPKGPTGDPGKNGDKGHAGLAGARGA

PGPDGNNGAQGPPGPQGVQGGKGEQGPAGPPGFQGLPGPSGPAGELGKPGERGLPGEFGL

PGPAGPRGERGPPGESGAAGPTGPIGSRGPSGPPGPDGNKGEPGVVGAAGTAGPSGPSGL

PGERGAAGIPGGKGEKGEPGLRGEIGNPGRDGARGAPGAVGAPGPAGATGDRGEAGAAGP

AGPAGPRGSPGERGEVGPAGPNGFAGPAGAAGQPGAKGERGAKGPKGENGXVGPTGPVGA

AGPAGPNGPPGPAGSRGDGGPPGMTGFPGAAGRTGPPGPSGISGPPGPPGPAGKEGLRGP

RGDQGPVGRTGETGAVGPPGFAGEKGPSGEAGTAGPPGTPGPQGLLGAPGILGLPGSRGE

RGLPGVAGAVGEPGPLGIAGPPGARGPPGAVGSPGVNGAPGEAGRDGNPGNDGPPGRDGQ

PGHKGERGYPGNIGPVGAAGAPGPHGPVGPAGKHGNRGETGPSGPVGPAGAVGPRGPSGP

QGIRGDKGEPGDKGPRGLPGLKGHNGLQGLPGLAGHHGDQGAPGSVGPAGPRGPAGPSGP

AGKDGRTGHPGTVGPAGIRGPQGHQGPAGPPGPPGPPGPPGVSGGGYDFGYDGDFFRA

>Tarsius

QMSYGYDEKSAG-ISVPGPMGPSGPRGLPGPPGAPGPQGFQGPPGEPGEPGASGPMGPRG

PPGPPGKNGDDGEAGKPGRPGERGPPGPQGARGLPGTAGLPGMKGHRGFSGLDGAKGDAG

PAGPKGEPGSPGENGAPGQMGPRGLPGERGRPGAPGPAGARGNDGATGAAGPPGPTGPAG

PPGFPGAVGAKGEAGPQGPRGSEGPQGVRGEPGPPGPAGAAGPAGNPGADGQPGAKGANG

APGIAGAPGFPGARGPSGPQGPGGPPGPKGNSGEPGAPGSKGDTGAKGEPGPVGVQGPPG

PAGEEGKRGARGEPGPTGLPGPPGERGGPGSRGFPGADGVAGPKGPAGERGSPGPAGPKG

SPGEAGRPGEAGLPGAKGLTGSPGSPGPDGKTGPPGPAGQDGRPGPPGPPGARGQAGVMG

FPGPKGAAGEPGKAGERGVPGPPGAVGPAGKDGEAGAQGPPGPAGPAGERGEQGPAGSPG

FQGLPGPAGPPGEAGKPGEQGVPGDLGAPGPSGARGERGFPGERGVQGPPGPAGPRGSNG

SPGNDGAKGDAGAPGAPGGQGAPGLQGMPGERGAPGLPGPKGDRGDAGPKGADGAPVKDG

VRGLTGPIGPPGPAGAPGDKGEXGPSGPAGPTGARGAPGDRGEPGPPGPAGFAGPPGADG

QPGAKGEPGDAGAKGDAGPPGPAGPAGPPGPIGNVGAPGAKGARGSAGPPGATGFPGAAG

RVGPPGPSGNAGPPGPPGPAGKEGGKGPRGETGPAGRPGEVGPPGPPGPAGEKGSPGADG

PAGAPGTPGPQGIAGQRGVVGLPGQRGERGFPGLPGPSGEPGKQGPSGXSGERGPPGPMG

PPGLAGPPGESGREGAPGAEGSPGRDGSPGAKGDRGETGPAGPPGAPGAPGAPGPVGPAG

KSGDRGETGPAGPAGPVGPVGARGPAGPQGPRGDKGETGEQGDRGIKGHRGFSGLQGPPG

PPGSPGEQGPSGASGPAGPRGPPGSAGAPGKDGLNGLPGPIGPPGPRGRTGDAGPXGPPG

PPGPPGPPGPPSXGXDFSFLPQPPQEKAHDGGRYYRARQYDGKGVGXGPGPMGLMGPRGP

PGASGAPGPQGFQGPAGEPGEPGQTGPAGARGPTGAPGKAGEDGHPGKPGRPGERGIVGP

QGARGFPGTPGLPGFKGPRGHSGPDGLKGQPGAAGVKGEPGAPGENGTPGQTGARGLPGE

RGRVGAPGPGGARGSDGSVGPVGPAGPIGSAGPPGFPGAPGPKGELGGVGNPGPSGPAGP

RGEVGLPGLSGPVGPPGNPGANGLTGAKGAAGLPGVAGAPGLPGPRGIPGPVGATGATGA

RGLVGEPGPAGTKGETGSKGEPGSAGAQGSPGPSGEEGKRGPNGESGSAGPTGPPGLRGS

PGSRGLPGADGRAGGMGLPGNRGATGPAGVRGPNGDSGRPGEPGLMGPRGLPGSPGNVGP

TGKEGPMGLPGIDGRPGAIGPAGARGEPGNIGFPGPKGPTGEPGKNGDKGHPGLAGARGA

PGPDGNNGAQGPPGPQGVQGGKGEQGPAGPPGFQGLPGPAGTAGEAGKPGERGLPGEFGL

PGPAGPRGERGPPGESGAAGPSGPIGSRGPSGPPGPDGNKGEPGAVGAPGTAGASGPGGL

PGERGAGGIPGGKGEKGEPGLRGEIGNPGRDGARGAPGAMGAPGPAGASGDRGEAGAAGP

AGPAGPRGSPGERGEVGPAGPNGFAGPAGAAGQPGAKGERGAKGPKGENGAVGPTGPVGA

AGPSGPNGPPGPAGSRGDGGPPGMTGFPGAGGRTGPPGPSGISGPPGPPGPAGKEGLRGP

RGDQGPVGRTGETGASGPPGFAGEKGPSGEAGTAGPPGTPGPQGLLGAPGILGLPGSRGE

RGLPGVAGALGEPGPLGISGPPGARGPPGAVGSPGVNGAPGEAGRDGNPGNDGPPGRDGL

PGHKGERGYPGNIGPVGAAGAPGPHGPVGPAGKHGNRGEPGPAGSVGPTGAAGPRGPSGA

QGARGDKGEGGDKGPRGLPGLKGHVGLLGLPGLAGQHGDQGSPGPVGPAGPRGPAGPSGP

PGKDGRSGHPGTVGPAGIRGQQGHQGPAGPAGPPGPPGPPGVSGGGYDFGXDGEFYRA

>Rattus

QMSYGYDEKSAG-VSVPGPMGPSGPRGLPGPPGAPGPQGFQGPPGEPGEPGASGPMGPRG

PPGPPGKNGDDGEAGKPGRPGERGPPGPQGARGLPGTAGLPGMKGHRGFSGLDGAKGDTG

PAGPKGEPGSPGENGAPGQMGPRGLPGERGRPGPPGSAGARGNDGAVGAAGPPGPTGPTG

PPGFPGAAGAKGEAGPQGARGSEGPQGVRGEPGPPGPAGAAGPAGNPGADGQPGAKGANG

APGIAGAPGFPGARGPSGPQGPSGAPGPKGNSGEPGAPGNKGDTGAKGEPGPAGVQGPPG

PAGEEGKRGARGEPGPSGLPGPPGERGGPGSRGFPGADGVAGPKGPAGERGSPGPAGPKG

SPGEAGRPGEAGLPGAKGLTGSPGSPGPDGKTGPPGPAGQDGRPGPAGPPGARGQAGVMG

FPGPKGTAGEPGKAGERGVPGPPGAVGPAGKDGEAGAQGAPGPAGPAGERGEQGPAGSPG

FQGLPGPAGPPGEAGKPGEQGVPGDLGAPGPSGARGERGFPGERGVQGPPGPAGPRGNNG

APGNDGAKGDTGAPGAPGSQGAPGLQGMPGERGAAGLPGPKGDRGDAGPKGADGSPGKDG

VRGLTGPIGPPGPAGAPGDKGETGPSGPAGPTGARGAPGDRGEPGPPGPAGFAGPPGADG

QPGAKGEPGDTGVKGDAGPPGPAGPAGPPGPIGNVGAPGPKGSRGAAGPPGATGFPGAAG

RVGPPGPSGNAGPPGPPGPVGKEGGKGPRGETGPAGRPGEVGPPGPPGPAGEKGSPGADG

PAGSPGTPGPQGIAGQRGVVGLPGQRGERGFPGLPGPSGEPGKQGPSGASGERGPPGPMG

PPGLAGPPGESGREGSPGAEGSPGRDGAPGAKGDRGETGPAGPPGAPGAPGAPGPVGPAG

KNGDRGETGPAGPAGPIGPAGARGPAGPQGPRGDKGETGEQGDRGIKGHRGFSGLQGPPG

SPGSPGEQGPSGASGPAGPRGPPGSAGSPGKDGLNGLPGPIGPPGPRGRTGDSGPAGPPG

PPGPPGPPGPPSGGYDFSFLPQPPQEKSQDGGRYYRARQYSDKGVSAGPGPMGLMGPRGP

PGAVGAPGPQGFQGPAGEPGEPGQTGPAGSRGPAGPPGKAGEDGHPGKPGRPGERGVVGP

QGARGFPGTPGLPGFKGIRGHNGLDGLKGQPGAQGVKGEPGAPGENGTPGQAGARGLPGE

RGRVGAPGPAGARGSDGSVGPVGPAGPIGSAGPPGFPGAPGPKGELGPVGNPGPAGPAGP

RGEAGLPGLSGPVGPPGNPGANGLTGAKGATGLPGVAGAPGLPGPRGIPGPVGAAGATGP

RGLVGEPGPAGSKGETGNKGEPGSAGAQGPPGPSGEEGKRGSPGEPGSAGPAGPPGLRGS

PGSRGLPGADGRAGVMGPPGNRGSTGPAGVRGPNGDAGRPGEPGLMGPRGLPGSPGNVGP

AGKEGPVGLPGIDGRPGPIGPAGPRGEAGNIGFPGPKGPSGDPGKPGEKGHPGLAGARGA

PGPDGNNGAQGPPGPQGVQGGKGEQGPAGPPGFQGLPGPSGTAGEVGKPGERGLPGEFGL

PGPAGPRGERGPPGESGAAGPSGPIGIRGPSGAPGPDGNKGEAGAVGAPGSAGASGPGGL

PGERGAAGIPGGKGEKGETGLRGEIGNPGRDGARGAPGAIGAPGPAGASGDRGEAGAAGP

SGPAGPRGSPGERGEVGPAGPNGFAGPAGSAGQPGAKGEKGTKGPKGENGIVGPTGPVGA

AGPSGPNGPPGPAGSRGDGGPPGMTGFPGAAGRTGPPGPSGITGPPGPPGAAGKEGlRGP

RGDQGPVGRTGEIGASGPPGFAGEKGPSGEPGTTGPPGTAGPQGLLGAPGILGLPGSRGE

RGQPGIAGALGEPGPLGIAGPPGARGPPGAVGSPGVNGAPGEAGRDGNPGSDGPPGRDGQ

PGHKGERGYPGNIGPTGAAGAPGPHGSVGPAGKHGNRGEPGPAGSVGPVGAVGPRGPSGP

QGIRGDKGEPGDKGARGLPGLKGHNGLQGLPGLAGLHGDQGAPGPVGPAGPRGPAGPSGP

IGKDGRSGHPGPVGPAGVRGSQGSQGPAGPPGPPGPPGPPGVSGGGYDFGFEGGFYRA

>Mus

QMSYGYDEKSAG-VSVPGPMGPSGPRGLPGPPGAPGPQGFQGPPGEPGEPGGSGPMGPRG

PPGPPGKNGDDGEAGKPGRPGERGPPGPQGARGLPGTAGLPGMKGHRGFSGLDGAKGDAG

PAGPKGEPGSPGENGAPGQMGPRGLPGERGRPGPPGTAGARGNDGAVGAAGPPGPTGPTG

PPGFPGAVGAKGEAGPQGARGSEGPQGVRGEPGPPGPAGAAGPAGNPGADGQPGAKGANG

APGIAGAPGFPGARGPSGPQGPSGPPGPKGNSGEPGAPGNKGDTGAKGEPGATGVQGPPG

PAGEEGKRGARGEPGPSGLPGPPGERGGPGSRGFPGADGVAGPKGPSGERGAPGPAGPKG

SPGEAGRPGEAGLPGAKGLTGSPGSPGPDGKTGPPGPAGQDGRPGPAGPPGARGQAGVMG

FPGPKGTAGEPGKAGERGLPGPPGAVGPAGKDGEAGAQGAPGPAGPAGERGEQGPAGSPG

FQGLPGPAGPPGEAGKPGEQGVPGDLGAPGPSGARGERGFPGERGVQGPPGPAGPRGNNG

APGNDGAKGDTGAPGAPGSQGAPGLQGMPGERGAAGLPGPKGDRGDAGPKGADGSPGKDG

ARGLTGPIGPPGPAGAPGDKGEAGPSGPPGPTGARGAPGDRGEAGPPGPAGFAGPPGADG

QPGAKGEPGDTGVKGDAGPPGPAGPAGPPGPIGNVGAPGPKGPRGAAGPPGATGFPGAAG

RVGPPGPSGNAGPPGPPGPVGKEGGKGPRGETGPAGRPGEVGPPGPPGPAGEKGSPGADG

PAGSPGTPGPQGIAGQRGVVGLPGQRGERGFPGLPGPSGEPGKQGPSGSSGERGPPGPMG

PPGLAGPPGESGREGSPGAEGSPGRDGAPGAKGDRGETGPAGPPGAPGAPGAPGPVGPAG

KNGDRGETGPAGPAGPIGPAGARGPAGPQGPRGDKGETGEQGDRGIKGHRGFSGLQGPPG

SPGSPGEQGPSGASGPAGPRGPPGSAGSPGKDGLNGLPGPIGPPGPRGRTGDSGPAGPPG

PPGPPGPPGPPSGGYDFSFLPQPPQEKSQDGGRYYRARQYSDKGVSSGPGPMGLMGPRGP

PGAVGAPGPQGFQGPAGEPGEPGQTGPAGPRGPAGSPGKAGEDGHPGKPGRPGERGVVGP

QGARGFPGTPGLPGFKGVRGHSGMDGLKGQPGAQGVKGEPGAPGENGTPGQAGARGLPGE

RGRVGAPGPAGARGSDGSVGPVGPAGPIGSAGPPGFPGAPGPKGELGPVGNPGPAGPAGP

RGEVGLPGLSGPVGPPGNPGTNGLTGAKGATGLPGVAGAPGLPGPRGIPGPAGAAGATGA

RGLVGEPGPAGSKGESGNKGEPGSVGAQGPPGPSGEEGKRGSPGEAGSAGPAGPPGLRGS

PGSRGLPGADGRAGVMGPPGNRGSTGPAGIRGPNGDAGRPGEPGLMGPRGLPGSPGNVGP

SGKEGPVGLPGIDGRPGPIGPAGPRGEAGNIGFPGPKGPSGDPGKPGERGHPGLAGARGA

PGPDGNNGAQGPPGPQGVQGGKGEQGPAGPPGFQGLPGPSGTTGEVGKPGERGLPGEFGL

PGPAGPRGERGTPGESGAAGPSGPIGSRGPSGAPGPDGNKGEAGAVGAPGSAGASGPGGL

PGERGAAGIPGGKGEKGETGLRGDTGNTGRDGARGIPGAVGAPGPAGASGDRGEAGAAGP

SGPAGPRGSPGERGEVGPAGPNGFAGPAGAAGQPGAKGEKGTKGPKGENGIVGPTGSVGA

AGPSGPNGPPGPVGSRGDGGPPGMTGFPGAAGRTGPPGPSGIAGPPGPPGAAGKEGlRGP

RGDQGPVGRTGETGASGPPGFVGEKGPSGEPGTAGAPGTAGPQGLLGAPGILGLPGSRGE

RGLPGIAGALGEPGPLGISGPPGARGPPGAVGSPGVNGAPGEAGRDGNPGSDGPPGRDGQ

PGHKGERGYPGSIGPTGAAGAPGPHGSVGPAGKHGNRGEPGPAGSVGPVGAVGPRGPSGP

QGIRGDKGEPGDKGHRGLPGLKGYSGLQGLPGLAGLHGDQGAPGPVGPAGPRGPAGPSGP

VGKDGRSGQPGPVGPAGVRGSQGSQGPAGPPGPPGPPGPPGVSGGGYDFGFEGDFYRA

>Oryctolagus

QMSYGYDEKSAG-VSVPGPMGPSGPRGLPGPPGSPGPQGFQGPPGEPGEPGASGPMGPRG

PPGAPGKNGDDGEAGKPGRPGERGPPGPQGARGLPGTAGLPGMKGHRGFSGLDGAKGDAG

PAGPKGEPGSPGENGAPGQMGPRGLPGERGRPGAPGPAGARGNDGATGAAGPPGPTGPAG

PPGFPGAVGAKGEAGPQGARGSEGPQGVRGEPGPPGPAGAAGPAGNPGADGQPGAKGANG

APGIAGAPGFPGARGPSGPQGPSGPPGPKGNSGEPGAPGNKGDTGAKGEPGPTGVQGPPG

PAGEEGKRGARGEPGPTGLPGPPGERGGPGSRGFPGADGVAGPKGPAGERGAPGPAGPKG

SPGEAGRPGEAGLPGAKGLTGSPGSPGPDGKTGPPGPAGQDGRPGPPGPPGARGQAGVMG

FPGPKGAAGEPGKAGERGVPGPPGAVGPAGKDGEAGAQGPPGPAGPAGERGEQGPAGSPG

FQGLPGPAGPPGEAGKPGEQGVPGDLGAPGPSGARGERGFPGERGVQGPPGPAGPRGSNG

APGNDGAKGDAGAPGAPGSQGAPGLQGMPGERGAAGLPGPKGDRGDAGPKGADGSPGKDG

VRGLTGPIGPPGPAGAPGDKGETGPSGPAGPTGARGAPGDRGEPGPPGPAGFAGPPGADG

QPGAKGEPGDAGAKGDAGPAGPAGPAGPPGPIGNVGAPGPKGARGSPGPPGATGFPGAAG

RVGPPGPSGNAGPPGPPGPVGKEGGKGPRGETGPAGRPGEVGPPGPPGPAGEKGSPGADG

PAGAPGTPGPQGIAGQRGVVGLPGQRGERGFPGLPGPSGEPGKQGPSGASGERGPPGPMG

PPGLAGPPGESGREGSPGAEGSPGRDGAPGPKGDRGETGPAGPPGAPGAPGAPGPVGPAG

KSGDRGETGPAGPAGPIGPAGARGPAGPQGPRGDKGETGEQGDRGIKGHRGFSGLQGPPG

PPGSPGEQGPSGASGPAGPRGPPGSAGAPGRDGLNGLPGPIGPPGPRGRTGDAGPVGPPG

PPGPPGPPGPPSGGFDFSFLPQPPQEKAGDGGRYYRARQFDAKGVGLGPGPMGLMGPRGP

PGAAGAPGPQGFQGPAGEPGEPGQTGPAGARGPPGPPGKAGEDGHPGKPGRPGERGVMGP

QGARGFPGTPGLPGFKGIRGHNGLDGLKGQPGAPGVKGEPGAPGENGTPGQTGARGLPGE

RGRVGAPGPAGARGSDGSVGPVGPAGPIGSAGPPGFPGAPGPKGELGPVGNPGPSGPAGP

RGEVGLPGVSGPVGPPGNPGANGLTGAKGAAGLPGVAGAPGLPGPRGIPGPVGAAGATGA

RGLVGEPGPAGTKGESGNKGEPGSAGPQGPPGPSGEEGKRGSPGEPGSAGPAGPPGLRGS

PGSRGLPGADGRAGVMGPPGSRGSTGPAGVRGPNGDSGRPGEPGLMGPRGLPGSPGNVGP

AGKEGPVGLPGIDGRPGPIGPAGARGEAGNIGFPGLKGLTGDPGKNGDKGHPGLAGARGA

PGPDGNNGAQGSPGPQGVQGGKGEQGPAGPPGFQGLPGPSGTAGEVGKPGERGLPGEFGL

PGPAGPRGERGAPGESGAAGPPGPIGSRGPSGPPGPDGNKGEPGVVGAPGTAGASGPGGF

PGERGAAGIPGGKGEKGETGLRGEIGNPGRDGARGAPGAVGAPGPAGATGDRGEAGAAGP

AGPAGPRGSPGERGEVGPAGPNGFAGPAGAAGQPGAKGEKGTKGPKGENGVVGPAGPVGA

AGPSGPNGPPGPAGSRGDGGPPGMTGFPGAAGRTGPPGPSGITGPPGPPGAAGKEGLRGP

RGDQGPVGRTGETGASGPPGFPGEKGPSGEAGTAGPPGTPGPQGLLGAPGILGLPGSRGE

RGLPGVAGALGEPGPLGIAGPPGARGPPGAVGSPGVNGAPGEAGRDGNPGSDGPPGRDGQ

PGHKGERGYPGNAGPVGAAGAPGPQGSVGPTGKHGNRGEPGPAGSIGPVGAAGPRGPSGP

QGIRGDKGEPGDKGPRGLPGlKGHNGLQGLPGLAGQHGDQGAPGAVGPAGPRGPAGPTGP

AGKDGRSGHPGTVGPAGIRGSQGSQGPAGPPGPPGPPGPPGASGGGYDFGYDGDFYRA

>Homo

QLSYGYDEKSTGGISVPGPMGPSGPRGLPGPPGAPGPQGFQGPPGEPGEPGASGPMGPRG

PPGPPGKNGDDGEAGKPGRPGERGPPGPQGARGLPGTAGLPGMKGHRGFSGLDGAKGDAG

PAGPKGEPGSPGENGAPGQMGPRGLPGERGRPGAPGPAGARGNDGATGAAGPPGPTGPAG

PPGFPGAVGAKGEAGPQGPRGSEGPQGVRGEPGPPGPAGAAGPAGNPGADGQPGAKGANG

APGIAGAPGFPGARGPSGPQGPGGPPGPKGNSGEPGAPGSKGDTGAKGEPGPVGVQGPPG

PAGEEGKRGARGEPGPTGLPGPPGERGGPGSRGFPGADGVAGPKGPAGERGSPGPAGPKG

SPGEAGRPGEAGLPGAKGLTGSPGSPGPDGKTGPPGPAGQDGRPGPPGPPGARGQAGVMG

FPGPKGAAGEPGKAGERGVPGPPGAVGPAGKDGEAGAQGPPGPAGPAGERGEQGPAGSPG

FQGLPGPAGPPGEAGKPGEQGVPGDLGAPGPSGARGERGFPGERGVQGPPGPAGPRGANG

APGNDGAKGDAGAPGAPGSQGAPGLQGMPGERGAAGLPGPKGDRGDAGPKGADGSPGKDG

VRGLTGPIGPPGPAGAPGDKGESGPSGPAGPTGARGAPGDRGEPGPPGPAGFAGPPGADG

QPGAKGEPGDAGAKGDAGPPGPAGPAGPPGPIGNVGAPGAKGARGSAGPPGATGFPGAAG

RVGPPGPSGNAGPPGPPGPAGKEGGKGPRGETGPAGRPGEVGPPGPPGPAGEKGSPGADG

PAGAPGTPGPQGIAGQRGVVGLPGQRGERGFPGLPGPSGEPGKQGPSGASGERGPPGPMG

PPGLAGPPGESGREGAPGAEGSPGRDGSPGAKGDRGETGPAGPPGAPGAPGAPGPVGPAG

KSGDRGETGPAGPAGPVGPVGARGPAGPQGPRGDKGETGEQGDRGIKGHRGFSGLQGPPG

PPGSPGEqGPSGASGPAGPRGPPGSAGAPGKDGLNGLPGPIGPPGPRGRTGDAGPVGPPG

PPGPPGPPGPPSAGFDFSFLPQPPQEKAHDGGRYYRARQYDGKGVGLGPGPMGLMGPRGP

PGAAGPPGPQGFQGPAGEPGEPGQTGPAGARGPAGPPGKAGEDGHPGKPGRPGERGVVGP

QGARGFPGTPGLPGFKGIRGHNGLDGLKGQPGAPGVKGEPGAPGENGTPGQTGARGLPGE

RGRVGAPGPAGARGSDGSVGPVGPAGPIGSAGPPGFPGAPGPKGELGAVGNAGPAGPAGP

RGEVGLPGLSGPVGPPGNPGANGLTGAKGAAGLPGVAGAPGLPGPRGIPGPVGAAGATGA

RGLVGEPGPAGSKGESGNKGEPGSAGPQGPPGPSGEEGKRGPNGEAGSAGPPGPPGLRGS

PGSRGLPGADGRAGVMGPPGSRGASGPAGVRGPNGDAGRPGEPGLMGPRGLPGSPGNIGP

AGKEGPVGLPGIDGRPGPIGPAGARGEPGNIGFPGPKGPTGDPGKNGDKGHAGLAGARGA

PGPDGNNGAQGPPGPQGVQGGKGEQGPPGPPGFQGLPGPSGPAGEVGKPGERGLHGEFGL

PGPAGPRGERGPPGESGAAGPTGPIGSRGPSGPPGPDGNKGEPGVVGAVGTAGPSGPSGL

PGERGAAGIPGGKGEKGEPGLRGEIGNPGRDGARGAPGAVGAPGPAGATGDRGEAGAAGP

AGPAGPRGSPGERGEVGPAGPNGFAGPAGAAGQPGAKGERGAKGPKGENGVVGPTGPVGA

AGPAGPNGPPGPAGSRGDGGPPGMTGFPGAAGRTGPPGPSGISGPPGPPGPAGKEGLRGP

RGDQGPVGRTGEVGAVGPPGFAGEKGPSGEAGTAGPPGTPGPQGLLGAPGILGLPGSRGE

RGLPGVAGAVGEPGPLGIAGPPGARGPPGAVGSPGVNGAPGEAGRDGNPGNDGPPGRDGQ

PGHKGERGYPGNIGPVGAAGAPGPHGPVGPAGKHGNRGETGPSGPVGPAGAVGPRGPSGP

QGIRGDKGEPGEKGPRGLPGLKGHNGLQGLPGlAGHHGDQGAPGSVGPAGPRGPAGPSGP

AGKDGRTGHPGTVGPAGIRGPQGHQGPAGPPGPPGPPGPPGVSGGGYDFGYDGDFYRA

>Bos

QLSYGYDEKSTG-ISVPGPMGPSGPRGLPGPPGAPGPQGFQGPPGEPGEPGASGPMGPRG

PPGPPGKNGDDGEAGKPGRPGERGPPGPQGARGLPGTAGLPGMKGHRGFSGLDGAKGDAG

PAGPKGEPGSPGENGAPGQMGPRGLPGERGRPGAPGPAGARGNDGATGAAGPPGPTGPAG

PPGFPGAVGAKGEGGPQGPRGSEGPQGVRGEPGPPGPAGAAGPAGNPGADGQPGAKGANG

APGIAGAPGFPGARGPSGPQGPSGPPGPKGNSGEPGAPGSKGDTGAKGEPGPTGIQGPPG

PAGEEGKRGARGEPGPAGLPGPPGERGGPGSRGFPGADGVAGPKGPAGERGAPGPAGPKG

SPGEAGRPGEAGLPGAKGLTGSPGSPGPDGKTGPPGPAGQDGRPGPPGPPGARGQAGVMG

FPGPKGAAGEPGKAGERGVPGPPGAVGPAGKDGEAGAQGPPGPAGPAGERGEQGPAGSPG

FQGLPGPAGPPGEAGKPGEQGVPGDLGAPGPSGARGERGFPGERGVQGPPGPAGPRGANG

APGNDGAKGDAGAPGAPGSQGAPGLQGMPGERGAAGLPGPKGDRGDAGPKGADGAPGKDG

VRGLTGPIGPPGPAGAPGDKGEAGPSGPAGPTGARGAPGDRGEPGPPGPAGFAGPPGADG

QPGAKGEPGDAGAKGDAGPPGPAGPAGPPGPIGNVGAPGPKGARGSAGPPGATGFPGAAG

RVGPPGPSGNAGPPGPPGPAGKEGSKGPRGETGPAGRPGEVGPPGPPGPAGEKGAPGADG

PAGAPGTPGPQGIAGQRGVVGLPGQRGERGFPGLPGPSGEPGKQGPSGASGERGPPGPMG

PPGLAGPPGESGREGAPGAEGSPGRDGSPGAKGDRGETGPAGPPGAPGAPGAPGPVGPAG

KSGDRGETGPAGPAGPIGPVGARGPAGPQGPRGDKGETGEQGDRGIKGHRGFSGLQGPPG

PPGSPGEQGPSGASGPAGPRGPPGSAGSPGKDGLNGLPGPIGPPGPRGRTGDAGPAGPPG

PPGPPGPPGPPSGGYDLSFLPQPPQEKAHDGGRYYRARQFDAKG-G-GPGPMGLMGPRGP

PGASGAPGPQGFQGPPGEPGEPGQTGPAGARGPPGPPGKAGEDGHPGKPGRPGERGVVGP

QGARGFPGTPGLPGFKGIRGHNGLDGLKGQPGAPGVKGEPGAPGENGTPGQTGARGLPGE

RGRVGAPGPAGARGSDGSVGPVGPAGPIGSAGPPGFPGAPGPKGELGPVGNPGPAGPAGP

RGEVGLPGLSGPVGPPGNPGANGLPGAKGAAGLPGVAGAPGLPGPRGIPGPVGAAGATGA

RGLVGEPGPAGSKGESGNKGEPGAVGQPGPPGPSGEEGKRGSTGEIGPAGPPGPPGLRGN

PGSRGLPGADGRAGVMGPAGSRGATGPAGVRGPNGDSGRPGEPGLMGPRGFPGSPGNIGP

AGKEGPVGLPGIDGRPGPIGPAGARGEPGNIGFPGPKGPSGDPGKAGEKGHAGLAGARGA

PGPDGNNGAQGPPGLQGVQGGKGEQGPAGPPGFQGLPGPAGTAGEAGKPGERGLPGEFGL

PGPAGARGERGPPGESGAAGPTGPIGSRGPSGPPGPDGNKGEPGVVGAPGTAGPSGPSGL

PGERGAAGIPGGKGEKGETGLRGDIGSPGRDGARGAPGAIGAPGPAGANGDRGEAGPAGP

AGPAGPRGSPGERGEVGPAGPNGFAGPAGAAGQPGAKGERGTKGPKGENGPVGPTGPVGA

AGPSGPNGPPGPAGSRGDGGPPGATGFPGAAGRTGPPGPSGISGPPGPPGPAGKEGLRGP

RGDQGPVGRSGETGASGPPGFVGEKGPSGEPGTAGPPGTPGPQGLLGAPGFLGLPGSRGE

RGLPGVAGSVGEPGPLGIAGPPGARGPPGNVGNPGVNGAPGEAGRDGNPGNDGPPGRDGQ

PGHKGERGYPGNAGPVGAAGAPGPQGPVGPVGKHGNRGEPGPAGAVGPAGAVGPRGPSGP

QGIRGDKGEPGDKGPRGLPGLKGHNGLQGLPGLAGHHGDQGAPGAVGPAGPRGPAGPSGP

AGKDGRIGQPGAVGPAGIRGSQGSQGPAGPPGPPGPPGPPGPSGGGYEFGFDGDFYRA

>Ovis

QLSYGYDEKSTG-ISVPGPMGPSGPRGLPGPPGAPGPQGFQGPPGEPGEPGASGPMGPRG

PPGPPGKNGDDGEAGKPGRPGERGPPGPQGARGLPGTAGLPGMKGHRGFSGLDGAKGDAG

PAGPKGEPGSPGENGTPGQMGPRGLPGERGRPGAPGPAGARGNDGATGAAGPPGPTGPAG

PPGFPGAVGAKGEAGPQGPRGSEGPQGVRGEPGPPGPAGAAGPAGNPGADGQPGAKGANG

APGIAGAPGFPGARGPSGPQGPSGPPGPKGNSGEPGAPGSKGDTGAKGEPGPTGIQGPPG

PAGEEGKRGARGEPGPAGLPGPPGERGGPGSRGFPGADGVAGPKGPAGERGAPGPAGPKG

SPGEAGRPGEAGLPGAKGLTGSPGSPGPDGKTGPPGPAGQDGRPGPPGPPGARGQAGVMG

FPGPKGAAGEPGKAGERGVPGPPGAVGPAGKDGEAGAQGPPGPAGPAGERGEQGPAGSPG

FQGLPGPAGPPGEAGKPGEQGVPGDLGAPGPSGARGERGFPGERGVQGPPGPAGPRGANG

APGNDGAKGDAGAPGAPGSQGAPGLQGMPGERGAAGLPGPKGDRGDAGPKGADGAPGKDG

VRGLTGPIGPPGPAGAPGDKGETGPSGPAGPTGARGAPGDRGEPGPPGPAGFAGPPGADG

QPGAKGEPGDAGAKGDAGPPGPAGPAGPPGPIGNVGAPGPKGARGSAGPPGATGFPGAAG

RVGPPGPSGNAGPPGPPGPAGKEGSKGPRGETGPAGRAGEVGPPGPPGPAGEKGAPGADG

PAGAPGTPGPQGIAGQRGVVGLPGQRGERGFPGLPGPSGEPGKQGPSGASGERGPPGPMG

PPGLAGPPGESGREGAPGAEGSPGRDGAPGAKGDRGETGPAGPPGAPGAPGAPGPVGPAG

KSGDRGETGPAGPAGPIGPVGARGPAGPQGPRGDKGETGEQGDRGIKGHRGFSGLQGPPG

PPGSPGEQGPSGASGPAGPRGPPGSAGTPGKDGLNGLPGPIGPPGPRGRTGDAGPAGPPG

PPGPPGPPGPPSGGYDLSFLPQPPQEKAHDGGRYYRARQFDGKG-G-GPGPMGLMGPRGP

PGASGAPGPQGFQGPPGEPGEPGQTGPAGARGPPGPPGKAGEDGHPGKPGRPGERGVVGP

QGARGFPGTPGLPGFKGIRGHNGLDGLKGQPGAPGVKGEPGAPGENGTPGQTGARGLPGE

RGRVGAPGPAGARGSDGSVGPVGPAGPIGSAGPPGFPGAPGPKGELGPVGNPGPAGPAGP

RGEVGLPGLSGPVGPPGNPGANGLPGAKGAAGLPGVAGAPGLPGPRGIPGPVGAAGATGA

RGLVGEPGPAGSKGESGNKGEPGAVGQPGPPGPSGEEGKRGSTGEIGPAGPPGPPGLRGN

PGSRGLPGADGRAGVMGPAGSRGATGPAGVRGPNGDSGRPGEPGLMGPRGFPGSPGNIGP

AGKEGPAGLPGIDGRPGPIGPAGARGEPGNIGFPGPKGPTGDPGKAGEKGHAGLAGPRGA

PGPDGNNGAQGPPGLQGVQGGKGEQGPAGPPGFQGLPGPAGTAGEAGKPGERGLPGEFGL

PGPAGARGERGPPGESGAAGPTGPIGSRGPSGPPGPDGNKGEPGVVGAPGTAGPSGPSGL

PGERGAAGIPGGKGEKGETGLRGDVGSPGRDGARGAPGAVGAPGPAGANGDRGEAGPAGP

AGPAGPRGSPGERGEVGPAGPNGFAGPAGAAGQPGAKGERGTKGPKGENGPVGPTGPVGA

AGPSGPNGPPGPAGSRGDGGPPGATGFPGAAGRTGPPGPAGISGPPGPPGPAGKEGLRGP

RGDQGPVGRTGEPGAAGPPGFVGEKGPSGEPGTAGPPGTPGPQGLLGAPGFLGLPGSRGE

RGLPGVAGSVGEPGPLGIAGPPGARGPPGNVGNPGVNGAPGEAGRDGNPGNDGPPGRDGQ

PGHKGERGYPGNAGPVGAAGAPGPQGPVGPTGKHGNRGEPGPVGAVGPAGAVGPRGPSGP

QGIRGDKGEPGDKGPRGLPGLKGHNGLQGLPGLAGHHGDQGAPGAVGPAGPRGPAGPTGP

AGKDGRTGQPGAVGPAGIRGSQGSQGPAGPPGPPGPPGPPGPSGGGYDFGFDGDFYRA

>Sus

QLSYGYDEKSAG-ISVPGPMGPSGPRGLPGPPGAPGPQGFQGPPGEPGEPGASGPMGPRG

PPGPPGKNGDDGEAGKPGRPGERGPPGPQGARGLPGPAGLPGMKGHRGFSGLDGAKGDAG

PAGPKGEPGSPGENGAPGQMGPRGLPGERGRPGPPGTAGARGNDGATGAAGPPGPTGPAG

PPGFPGAVGAKGEAGPQGARGSEGPQGVRGEPGPPGPAGAAGPAGNPGADGQPGAKGANG

APGIAGAPGFPGARGPSGPQGPSGPPGPKGNSGEPGAPGSKGDTGAKGEPGPTGVQGPPG

PAGEEGKRGARGEPGPAGLPGPPGERGGPGSRGFPGADGVAGPKGPAGERGSPGPAGPKG

SPGEAGRPGEAGLPGAKGLTGSPGSPGPDGKTGPPGPAGQDGRPGPPGPPGARGQAGVMG

FPGPKGAAGEPGKAGERGVPGPPGAVGPAGKDGEAGAQGPPGPAGPAGERGEQGPAGSPG

FQGLPGPAGPPGEAGKPGEQGVPGDLGAPGPSGARGERGFPGERGVQGPPGPAGPRGANG

APGNDGAKGDAGAPGAPGSQGAPGLQGMPGERGAAGLPGPKGDRGDAGPKGADGAPGKDG

VRGLTGPIGPPGPAGAPGDKGETGPSGPAGPTGARGAPGDRGEPGPPGPAGFAGPPGADG

QPGAKGEPGDAGAKGDAGPPGPAGPTGPPGPIGSVGAPGPKGARGSAGPPGATGFPGAAG

RVGPPGPSGNAGPPGPPGPAGKEGSKGPRGETGPAGRPGEVGPPGPPGPAGEKGSPGADG

PAGAPGTPGPQGIAGQRGVVGLPGQRGERGFPGLPGPSGEPGKQGPSGPSGERGPPGPMG

PPGLAGPPGESGREGAPGAEGSPGRDGAPGPKGDRGESGPAGPPGAPGAPGAPGPVGPAG

KSGDRGETGPAGPAGPVGPVGARGPAGPQGPRGDKGETGEQGDRGIKGHRGFSGLQGPPG

PPGSPGEQGPSGASGPAGPRGPPGSAGAPGKDGLNGLPGPIGPPGPRGRTGDAGPVGPPG

PPGPPGPPGPPSGGFDFSFLPQPPQEKAHDGGRYYRARQYDGKGVGAGPGPMGLMGPRGP

PGAVGAPGPQGFQGPAGEPGEPGQTGPAGARGPPGPPGKAGEDGHPGKPGRPGERGVVGP

QGARGFPGTPGLPGFKGIRGHNGLDGLKGQPGAPGVKGEPGAPGENGTPGQTGARGLPGE

RGRVGAPGPAGARGNDGSVGPVGPAGPIGSAGPPGFPGAPGPKGELGPVGNPGPAGPAGP

RGEVGLPGVSGPVGPPGNPGANGLPGAKGAAGLPGVAGAPGLPGPRGIPGPAGAAGATGA

RGLVGEPGPAGSKGESGNKGEPGAAGPQGPPGPSGEEGKRGPNGEVGSAGPPGPPGLRGN

PGSRGLPGADGRAGVMGPPGSRGPTGPAGVRGPNGDSGRPGEPGLMGPRGFPGSPGNVGP

AGKEGPAGLPGIDGRPGPIGPAGARGEPGNIGFPGPKGPTGDPGKNGEKGHAGLAGARGA

PGPDGNNGAQGPPGPQGVQGGKGEQGPAGPPGFQGLPGPAGTAGEVGKPGERGLPGEFGL

PGPAGPRGERGPPGESGAAGPAGPIGSRGPSGPPGPDGNKGEPGVLGAPGTAGPSGPSGL

PGERGAAGIPGGKGEKGETGLRGDVGSPGRDGARGAPGAVGAPGPAGANGDRGEAGPAGP

AGPAGPRGSPGERGEVGPAGPNGFAGPAGAAGQPGAKGERGTKGPKGENGPVGPTGPVGA

AGPAGPNGPPGPAGSRGDGGPPGATGFPGAAGRIGPPGPSGISGPPGPPGPAGKEGLRGP

RGDQGPVGRTGETGASGPPGFAGEKGPSGEPGTAGPPGTPGPQGlLGAPGFLGLPGSRGE

RGLPGVAGSVGEPGPLGIAGPPGARGPPGAVGNPGVNGAPGEAGRDGNPGSDGPPGRDGQ

AGHKGERGYPGNPGPAGAAGAPGPQGAVGPAGKHGNRGEPGPAGSVGPAGAVGPRGPSGP

QGIRGDKGEPGDKGPRGLPGLKGHNGLQGLPGLAGHHGDQGAPGPVGPAGPRGPAGPSGP

AGKDGRTGQPGAVGPAGIRGSQGSQGPAGPPGPPGPPGPPGPSGGGYDFGYEGDFYRA

>Ochotona

QMSYGYDEKSAG-VSVPGPMGPSGPRGLPGPPGSPGPQGFQGPPGEPGEPGASGPMGPRG

PPGPPGKNGDDGEAGKPGRPGERGPPGPQGARGLTGTAGLPGMKGHRGFSGLDGAKGDAG

PAGPKGEPGSPGENGAPGQMGPRGLPGERGRPGPPGTAGARGNDGATGAAGPPGPTGPAG

PPGFPGAVGAKGEAGPQGARGSEGPQGIRGEPGPPGPAGAAGPAGNPGADGQPGAKGANG

APGIAGAPGFPGARGPSGPQGPSGPPGPKGNSGEPGAPGNKGDTGAKGEPGPAGVQGPPG

PAGEEGKRGARGEPGPAGLPGPPGERGGPGSRGFPGADGVAGPKGPAGERGAPGPAGPKG

SPGEAGRPGEAGLPGAKGLTGSPGSPGPDGKTGPPGPAGQDGRPGPPGPPGARGQAGVMG

FPGPKGAAGEPGKAGERGVPGPPGAVGAPGKDGEAGAQGPPGPAGPAGERGEQGPAGSPG

FQGLPGPAGPPGEAGKPGEQGVPGDLGAPGPSGARGERGFPGERGVQGPPGPAGPRGSNG

APGNDGAKGDAGAPGAPGSQGAPGLQGMPGERGAAGLPGPKGDRGDAGPKGADGSPGKDG

VRGLTGPIGPPGPAGAPGDKGETGPSGPAGPTGARGAPGDRGEPGPPGPAGFAGPPGADG

QPGAKGEPGDAGAKGDAGPPGPAGPAGPPGPIGNVGAPGPKGARGSAGPPGATGFPGAAG

RVGPPGPSGNAGPPGPPGPAGKEGGKGPRGETGPAGRPGEVGPPGPPGPAGEKGSPGADG

PAGAPGTPGPQGITGQRGVVGLPGQRGERGFPGLPGPSGEPGKQGPSGASGERGPPGPMG

PPGLAGPPGESGREGSPGAEGSPGRDGSPGPKGDRGETGPAGPPGAPGAPGAPGPVGPAG

KSGDRGETGPAGPAGPIGPAGARGPAGPQGPRGDKGETGEQGDRGIKGHRGFSGLQGPPG

PPGSPGEQGPSGASGPAGPRGPPGSAGAPGKDGLNGLPGPIGPPGXXXXXXXXXXXGPPG

PPGPPGPPGPPSGGFDFIFLPQPPQEKAHDGGRYYRARQYDXKGXXXXXXXMGLMGPRGP

PGAAGAPGPQGFQGPAGEPGEPGQTGPAGARGPPGAPGKAGEDGHPGKPGRPGERGIMGP

QGARGFPGTPGLPGFKGIRGHNGLDGLKGQPGAPGVKGEPGAPGENGTPGQTGARGLPGE

RGRVGAPGPAGARGSDGSVGPVGPAGPIGSAGPPGFPGAPGPKGELGPVGNPGPSGPAGP

RGEVGLPGVSGPVGPPGNPGTNGLTGAKGAAGLPGVAGAPGLPGPRGLPGPVGAAGATGA

RGLVGEPGPAGSKGESGNKGEPGSAGPQGPPGPSGEEGKRGSTGEPGSAGPPGPPGLRGS

PGSRGLPGADGRAGVMGPPGSRGSTGPAGVRGPNGDSGRPGEPGLVGPRGLPGSPGNVGP

AGKEGPVGLPGIDGRPGPIGPAGARGEPGNIGFPGPKGPSGDAGKSGDKGHPGLAGARGA

PGPDGNNGAQGPPGPQGVQGGKGEQGPAGPPGFQGLPGPSGPAGEVGKPGERGLPGEFGL

PGPAGARGERGPPGESGAAGPPGPIGSRGPSGPPGPDGNKGEPGAVGAPGNAGASGPGGL

PGERGAAGIPGGKGEKGETGLRGEVGNPGRDGARGAPGAVGAPGPAGATGDRGEAGAAGP

AGPAGPRGSPGERGEVGPAGPNGFAGPAGAAGQPGAKGERGTKGPKGENGVVGPTGPVGA

AGPSGPNGPPGPVGGRGDGGPPGMTGFPGAAGRTGPPGPSGITGPPGPPGAAGKEGLRGP

RGDQGPVGRTGEPGAAGPPGFAGEKGPSGEAGTAGPPGTPGPQGLLGPPGILGLPGTRGE

RGLPGVAGALGEPGPLGVAGPPGARGPPGAVGSPGVNGAPGEAGRDGNPGSDGPPGRDGQ

PGHKGERGYPGNAGPAGAAGAPGPQGSVGPTGKHGNRGEPGPAGSVGPVGAVGPRGPSGP

QGIRGDKGEPGDKGPRGLPGLKGHNGLQGLPGLAGQHGDQGAPGAVGPAGPRGPAGPTGP

AGKDGRSGHPGTVGPAGVRGSQGSQGPAGPPGPPGPPGPPGASGGGYDFGYDGDFYRA

>Equus

QLSYGYDEKSAG-ISVPGPMGPSGPRGLPGPPGAPGPQGFQGPPGEPGEPGASGPMGPRG

PPGPPGKNGDDGEAGKPGRPGERGPPGPQGARGLPGTAGLPGMKGHRGFSGLDGAKGDAG

PAGPKGEPGSPGENGAPGQMGPRGLPGERGRPGAPGPAGARGNDGATGAAGPPGPTGPAG

PPGFPGAVGAKGEAGPQGARGSEGPQGVRGEPGPPGPAGAAGPAGNPGADGQPGAKGANG

APGIAGAPGFPGARGPSGPQGPSGPPGPKGNSGEPGAPGNKGDTGAKGEPGPTGIQGPPG

PAGEEGKRGARGEPGPTGLPGPPGERGGPGARGFPGADGVAGPKGPAGERGAPGPAGPKG

SPGEAGRPGEAGLPGAKGLTGSPGSPGPDGKTGPPGPAGQDGRPGPPGPPGARGQAGVMG

FPGPKGAAGEPGKAGERGVPGPPGAVGPAGKDGEAGAQGPPGPAGPAGERGEQGPAGSPG

FQGLPGPAGPPGEAGKPGEQGVPGDLGAPGPSGARGERGFPGERGVQGPPGPAGPRGSNG

APGNDGAKGDAGAPGAPGSQGAPGLQGMPGERGAAGLPGPKGDRGDAGPKGADGSPGKDG

VRGLTGPIGPPGPAGAPGDKGETGPSGPAGPTGARGAPGDRGEPGPPGPAGFAGPPGADG

QPGAKGEPGDAGAKGDAGPPGPAGPAGPPGPIGNVGAPGPKGARGSAGPPGATGFPGAAG

RVGPPGPSGNAGPPGPPGPVVKEGGKGPRGETGPAGRPGEVGPPGPPGPAGEKGSPGADG

PAGAPGTPGPQGIAGQRGVVGLPGQRGERGFPGLPGPSGEPGKQGPSGASGERGPPGPVG

PPGLAGPPGESGREGSPGAEGSAGRDGSPGPKGDRGETGPAGPPGAPGAPGAPGPVGPAG

KSGDRGEAGPAGPAGPIGPVGARGPAGPQGPRGDKGETGEQGDRGIKGHRGFSGLQGPPG

PPGSPGEQGPSGASGPAGPRGPPGSAGAPGKDGLNGLPGPIGPPGPRGRTGDAGPVGPPG

PPGPPGPPGPPSGGFDFSFLPQPPQEKSHDGGRYYRARQFDAKGXGLGPGPMGLMGPRGP

PGASGAPGPQGFQGPAGEPGEPGQTGPAGARGPPGPPGKAGEDGHPGKPGRPGERGVVGP

QGARGFPGTPGLPGFKGIRGHNGLDGLKGQPGAPGVKGEPGAPGENGTPGQAGARGLPGE

RGRVGAPGPAGARGSDGSVGPVGPAGPIGSAGPPGFPGAPGPKGELGPVGNPGPAGPAGP

RGEVGLPGLSGPVGPPGNPGANGLTGAKGAAGLPGVAGAPGLPGPRGIPGPAGAAGATGA

RGLVGEPGPAGSKGESGNKGEPGAAGPQGPPGPSGEEGKRGPNGEPGSTGPAGPPGLRGS

PGSRGLPGADGRAGVMGPAGSRGASGPAGVRGPNGDSGRPGEPGLMGPRGFPGSPGNIGP

AGKEGPVGLPGIDGRPGPIGPAGARGEPGNIGFPGPKGPSGEPGKPGDKGHAGLAGARGA

PGPDGNNGAQGPPGPQGVQGGKGEQGPAGPPGFQGLPGPAGTAGEVGKPGERGLPGEFGL

PGPAGARGERGPPGESGAAGPAGPIGSRGPSGPPGPDGNKGEPGVLGAPGTAGPSGPSGL

PGERGAAGIPGGKGEKGETGLRGEIGNPGRDGARGAPGAVGAPGPAGANGDRGEAGAAGP

AGPAGPRGSPGERGEVGPAGPNGFAGPAGAAGQPGAKGERGTKGPKGENGPVGPTGPVGA

AGPSGPNGPPGPAGSRGDGGPPGVTGFPGAAGRTGPPGPSGISGPPGPPGAAGKEGLRGP

RGDQGPVGRTGETGASGPPGFAGEKGPSGEPGTAGPPGTPGPQGLLGAPGILGLPGSRGE

RGLPGVAGSLGEPGPLGIAGPPGARGPPGAVGAPGVNGAPGEAGRDGNPGSDGPPGRDGQ

PGHKGERGYPGNAGPVGAVGAPGPHGPVGPTGKHGNRGEPGPVGSVGPVGAVGPRGPSGP

QGVRGDKGEPGDKGPRGLPGlKGHNGLQGLPGLAGQHGDQGAPGSVGPAGPRGPAGPTGP

VGKDGRSGQPGTVGPAGVRGSQGSQGPAGPPGPPGPPGPPGPSGGGYDFGYDGDFYRA

>Sarcophilus

QMSYGYDEKSGGGMSVPGPMGPSGPRGLPGPPGSPGPQGFQGPPGEPGEPGASGPMGPRG

PAGPPGKNGDDGEAGKPGRPGERGPPGPQGARGLPGTAGLPGMKGHRGFSGLDGAKGDSG

PAGPKGEPGSPGENGAPGQMGPRGLPGERGRPGPPGPAGARGNDGATGAAGPPGPTGPAG

PPGFPGAVGAKGEAGPQGSRGSEGPQGVRGEPGPPGPAGSPGPSGNPGADGQPGAKGANG

APGIAGAPGFPGARGPSGPQGPSGAPGPKGNSGEPGTPGNKGDPGAKGEPGPVGVQGPPG

PAGEEGKRGSRGEPGPAGLPGPAGERGGPGSRGFPGADGVAGPKGAPGERGAPGPAGPKG

SPGESGRPGEAGLPGAKGLTGSPGSPGPDGKTGPPGPAGQDGRPGPPGPPGARGQAGVMG

FPGPKGAAGEPGKAGERGVPGPPGAVGPAGKDGEAGAQGAPGPAGPAGERGEQGPAGSPG

FQGLPGPAGPPGEAGKPGEQGVPGDAGAPGPSGARGERGFPGERGVQGPPGPQGPRGANG

APGNDGAKGDAGAPGAPGGQGPPGLQGMPGERGAAGLPGAKGDRGDAGPKGADGAPGKDG

VRGLTGPIGPPGPAGPSGDKGESGPSGPAGPTGARGAPGERGEPGPPGPAGFAGPPGADG

QPGAKGEPGDAGAKGDAGPPGPAGPTGAPGPAGNVGAPGPKGARGSAGPPGATGFPGAAG

RVGPPGPSGNAGPPGPPGPAGKEGGKGPRGETGPIGRPGEVGPPGPPGPSGEKGSPGADG

PAGAPGTPGPQGIAGQRGVVGLPGQRGERGFPGLPGPSGEPGKQGPSGVSGERGPPGPAG

PPGLAGPPGESGREGSPGAEGSPGRDGAPGPKGDRGETGPAGPPGAPGAPGAPGPVGPAG

KAGDRGETGPSGPAGPAGPTGARGPAGPQGPRGDKGETGEQGDRGMKGHRGFSGLQGPPG

PPGSPGEQGPSGASGPAGPRGPPGSAGAAGKDGLNGLPGPIGPPGPRGRTGDAGPAGPPG

PPGPPGPPGPPSGGFDFSFLPQPPQEKAHDSGRYYRARQYDAKGLDMGPGPMGLMGPRGP

PGASGPPGAQGFQGPAGEPGEPGQTGPAGARGPPGPPGKSGEDGHPGKPGRPGERGIVGP

QGARGFPGTPGLPGFKGIRGHNGLDGLKGQAGAPGVKGEPGAPGENGTPGQAGARGLPGE

RGRIGGPGPAGARGSDGSVGPVGPAGPIGSAGPPGFPGAPGPKGELGPVGNPGPAGPAGP

RGELGLPGMTGPVGPAGNPGANGLTGAKGAAGLPGVAGAPGLPGPRGIPGPAGAAGASGP

RGLAGEPGPAGSKGESGNKGEPGSAGPQGPPGPNGEEGKRGPNGEPGSTGPMGPPGLRGV

PGSRGLPGADGRAGGMGPPGNRGPSGPAGARGPNGDAGRPGEPGLMGPRGLPGSPGNVGP

TGKEGPAGLPGIDGRPGPTGPAGNRGEPGNIGFPGPKGPNGDPGKAGEKGHAGLAGARGA

PGPDGNNGAQGPPGPTGVQGGKGEQGPAGPPGFQGLPGPSGPAGEGGKVGERGLPGEFGL

PGPAGPRGERGPPGESGAVGPTGSIGSRGPSGPPGPDGNKGEPGVVGAPGNAGPAGSGGV

PGERGAAGVPGGKGEKGETGPRGEFGNPGRDGARGAPGAMGAPGPAGATGERGEAGPAGP

VGPTGNRGAPGDRGEAGPAGPNGFAGPPGAAGQAGAKGERGTKGPKGENGIVGPTGPVGA

AGPAGPNGPPGPVGGRGDGGPPGXTGFPGAAGRPGXPGPXGLEGMTGFPGAAGKEGPRGP

RGDQGPLGRAGETGAVGPPGFAGEKGPPGEAGASGPPGSSGPQGLLGAPGILGLPGSRGE

RGLPGVSGSLGEPGPLGISGPPGARGPPGAVGSPGVNGAPGEAGRDGNPGNDGPPGRDGL

AGHKGERGYPGNPGAVGNAGAPGPHGTVGPAGKPGNRGEPGPVGSVGPVGPFGARGPSGP

QGPRGDKGEVGDKGPRGMNGFKGHNGFQGLPGlSGQHGDQGAPGSTGPAGPRGPAGPSGP

PGKDGRPGHAGAVGPAGLRGSQGSQGPAGPPGPPGLPGPPGPSGGGYDFGYEGDFYRA

>Ailuropoda

QMSYGYDEKSTGGISVPGPMGPSGPRGLPGPPGAPGPQGFQGPPGEPGEPGASGPMGPRG

PPGPPGKNGDDGEAGKPGRPGERGPPGPQGARGLPGTAGLPGMKGHRGFSGLDGAKGDAG

PAGPKGEPGSPGENGAPGQMGPRGLPGERGRPGAPGPAGARGNDGATGAAGPPGPTGPAG

PPGFPGAVGAKGEAGPQGARGSEGPQGVRGEPGPPGPAGAAGPAGNPGADGQPGAKGANG

APGIAGAPGFPGARGPSGPQGPSGPPGPKGNSGEPGAPGNKGDTGAKGEPGPTGIQGPPG

PAGEEGKRGARGEPGPTGLPGPPGERGGPGSRGFPGADGVAGPKGPAGERGSPGPAGPKG

SPGEAGRPGEAGLPGAKGLTGSPGSPGPDGKTGPPGPAGQDGRPGPPGPPGARGQAGVMG

FPGPKGAAGEPGKAGERGVPGPPGAVGPAGKDGEAGAQGPPGPAGPAGERGEQGPAGSPG

FQGLPGPAGPPGEAGKPGEQGVPGDLGAPGPSGARGERGFPGERGVQGPPGPAGPRGANG

APGNDGAKGDAGAPGAPGSQGAPGLQGMPGERGAAGLPGPKGDRGDAGPKGADGSPGKDG

VRGLTGPIGPPGPAGAPGDKGEAGPSGPAGPTGARGAPGDRGEPGPPGPAGFAGPPGADG

QPGAKGEPGDAGAKGDAGPPGPAGPTGPPGPIGNVGAPGPKGARGSAGPPGATGFPGAAG

RVGPPGPSGNAGPPGPPGPAGKEGGKGPRGETGPAGRPGEVGPPGPPGPAGEKGSPGADG

PAGAPGTPGPQGIAGQRGVVGLPGQRGERGFPGLPGPSGEPGKQGPSGASGERGPPGPMG

PPGLAGPPGESGREGSPGAEGSPGRDGSPGPKGDRGETGPAGPPGAPGAPGAPGPVGPAG

KSGDRGETGPAGPAGPIGPVGARGPAGPQGPRGDKGETGEQGDRGIKGHRGFSGLQGPPG

PPGSPGEQGPSGASGPAGPRGPPGSAGSPGKDGLNGLPGPIGPPGPRGRTGDAGPVGPPG

PPGPPGPPGPPSGGFDFSFLPQPPQEKAHDGGRYYRARQYDGKGVGLGPGPMGLMGPRGP

PGASGAPGPQGFQGPAGEPGEPGQTGPAGARGPPGPPGKAGEDGHPGKPGRPGERGVVGP

QGARGFPGTPGLPGFKGIRGHNGLDGLKGQPGAPGVKGEPGAPGENGTPGQTGARGLPGE

RGRVGAPGPAGARGSDGSVGPVGPAGPIGSAGPPGFPGAPGPKGELGPVGNPGPAGPAGP

RGEVGLPGVSGPVGPPGNPGANGLTGAKGAAGLPGVAGAPGLPGPRGIPGPVGAAGATGA

RGLVGEPGPAGSKGESGNKGEPGSAGXQGPPGPSGEEGKRGPNGEAGSAGPSGPPGLRGS

PGSRGLPGADGRAGVMGPPGPRGSTGPAGVRGPNGDSGRPGEPGLMGPRGFPGAPGNVGP

AGKEGPMGLPGIDGRPGPIGPAGARGEPGNIGFPGPKGPTGDPGKAGEKGHAGLAGARGA

PGPDGNNGAQGPPGPQGVQGGKGEQGPAGPPGFQGLPGPAGTAGEVGKPGERGLPGEFGL

PGPAGPRGERGPPGESGAAGPSGPIGSRGPSGPPGPDGNKGEPGVLGAPGTAGPSGPGGL

PGERGAAGVPGGKGEKGETGLRGEVGNPGRDGARGAPGAVGAPGPAGATGDRGEAGPAGP

AGPAGPRGSPGERGEVGPAGPNGFAGPAGAAGQPGAKGERGTKGPKGENGPVGPTGPVGS

AGPSGPNGPPGPAGSRGDGGPPGATGFPGAAGRTGPPGPSGITGPPGPPGAAGKEGLRGP

RGDQGPVGRTGETGAHGPPGFAGEKGPSGEPGTAGPPGTAGPQGLLGAPGILGLPGSRGE

RGLPGVSGSVGEPGPLGIAGPPGARGPPGAVGAPGVNGAPGEAGRDGNPGNDGPPGRDGQ

PGHKGERGYPGNIGPVGTVGAPGPHGPVGPTGKHGNRGEPGPAGAVGPVGAVGPRGPSGP

QGVRGDKGEPGDKGPRGLPGLKGHNGLQGLPGLAGQHGDQGAPGSVGPAGPRGPAGPSGP

AGKDGRTGHPGTVGPAGVRGSQGSQGPAGPPGPPGPPGPPGPSGGGYDFGYEGDFYRA

>Myotis

QMSYGYDEKSAG-VSVPGPMGPSGPRGLPGPPGSPGPQGFQGPPGEPGEPGASGPMGPRG

PPGPPGKNGDDGEAGKPGRPGERGPPGPQGARGLPGTAGLPGMKGHRGFSGLDGAKGDAG

PAGPKGEPGSPGENGVPGQMGPRGLPGERGRPGAPGPAGARGNDGATGAAGPPGPTGPAG

PPGFPGAVGAKGEAGPQGSRGSEGPQGVRGEPGPPGPAGAAGPAGNPGADGQPGAKGANG

APGIAGAPGFPGARGPSGPQGPSGAPGPKGNSGEPGAPGNKGDTGAKGEPGPTGIQGPPG

PAGEEGKRGARGEPGPAGLPGPPGERGGPGSRGFPGADGVAGPKGPAGERGSPGPAGPKG

SPGEAGRPGEAGLPGAKGLTGSPGSPGPDGKTGPTGPAGQDGRPGPPGPPGARGQAGVMG

FPGPKGAAGEPGKAGERGVPGPPGAVGPAGKDGEAGAQGAPGPAGPAGERGEQGPAGSPG

FQGLPGPAGPPGEAGKPGEQGAPGDLGAPGPSGARGERGFPGERGVQGPPGPAGPRGSNG

APGNDGAKGDAGAPGAPGSQGAPGLQGMPGERGAAGLPGPKGDRGDAGPKGADGAPGKDG

VRGLTGPIGPPGPAGAPGDKGETGPSGPAGPTGARGAPGDRGEPGPPGPAGFAGPPGADG

QPGAKGEPGDAGAKGDAGPAGPAGPAGPPGPIGNVGAPGPKGARGSAGPPGATGFPGAAG

RVGPPGPSGNAGPPGPPGPAGKEGGKGPRGETGPAGRPGEVGPPGPPGPAGEKGSPGSDG

PAGSPGTPGPQGIAGQRGVVGLPGQRGERGFPGLPGPSGEPGKQGPSGSSGERGPPGPMG

PPGLAGPPGESGREGSPGAEGSPGRDGSPGPKGDRGETGPAGPPGAPGAPGAPGPVGPAG

KSGDRGETGPAGPAGPIGPAGARGPAGPQGPRGDKGETGEQGDRGIKGHRGFSGLQGPPG

PPGSPGxQGPSGASGPAGPRGPPGSPGAAGKDGLNGLAGPIGPPGPRGRTGDAGPVGPPG

PPGPPGPPGPPSGGFDFSFLPQPPQEKAHDGGRYYRARQFDGKGVGGGPGPMGLMGPRGP

PGAAGAPGPQGFQGPAGEPGEPGQTGPAGSRGPAGPPGKAGEDGHPGKPGRPGERGVVGP

QGARGFPGTPGLPGFKGIRGHNGLDGLKGQPGAPGIKGEPGAPGENGTPGQTGARGLPGE

RGRVGAPGPAGARGSDGSVGPVGPAGPiGSAGPPGFPGAPGPKGELGPVGNPGPSGPAGP

RGEVGLPGLSGPVGPPGNPGANGLAGAKGAAGLPGVAGAPGLPGPRGIPGPPGAAGAAGP

RGLIGEPGPAGSKGETGNKGEPGSAGAQGPPGPSGEEGKRGTAGEAGPAGPPGPAGLRGN

PGSRGLPGADGRAGVMGPAGPRGATGPAGARGPNGDAGRPGEPGLMGPRGFPGSPGNVGP

AGKEGPVGLPGIDGRPGPIGPAGARGEPGNIGFPGPKGPTGDAGKPGERGHAGLAGARGA

PGPDGNNGAQGPPGPQGVQGGKGEQGPAGPPGFQGLPGPAGTAGEAGKPGERGLPGEFGL

PGPAGPRGERGPPGESGAVGPSGPIGSRGPSGPPGPDGNKGEPGSVGAPGSAGAPGPGGL

PGERGAAGIPGGKGDKGEPGLRGEMGTTGRDGARGAPGAMGAPGPSGASGDRGEAGAAGP

AGPAGPRGSPGERGEVGPAGPNGFAGPAGAAGQPGAKGERGTKGPKGENGVVGPTGPVGA

AGPSGPNGPPGPAGTRGDGGPPGMTGFPGAAGRTGPPGPSGITGPPGPPGASGKEGlRGP

RGDQGPVGRTGETGATGPPGFVGEKGPSGEPGAAGPPGTPGPQGLLGAPGILGLPGSRGE

RGLPGVSGSVGEPGPLGIAGPPGARGPPGAVGSPGVNGAPGEAGRDGNPGSDGPPGRDGQ

PGHKGDRGYPGNAGPVGTVGAPGPHGPVGPTGKHGNRGEPGPAGSVGPTGAVGPRGPSGA

QGIRGDKGEPGEKGPRGLPGLKGHNGLQGLPGLAGHHGDQGAPGTVGPAGPRGPAGPSGP

PGKDGRNGHPGVVGPAGIRGTQGSQGPAGPPGPPGPPGPPGISGGGYDFGFDGDFYRA

>Pteropus

QMSYGYDEKSAG-VSVPGPMGPSGPRGLPGPPGAPGPQGFQGPPGEPGEPGASGPMGPRG

PPGPPGKNGDDGEAGKPGRPGERGPPGPQGARGLPGTAGLPGMKGHRGFSGLDGAKGDSG

PAGPKGEPGSPGENGAPGQMGPRGLPGERGRPGAPGPAGARGNDGATGAAGPPGPTGPAG

PPGFPGAVGAKGEAGPQGSRGSEGPQGVRGEPGPPGPAGAAGPAGNPGADGQPGAKGANG

APGIAGAPGFPGARGPSGPQGPxGPPGPKGNSGEPGAPGNKGDxGAKGEPGPTGIQGPPG

PAGEEGKRGARGEPGPSGLPGPPGERGGPGSRGFPGADGVAGPKGPAGERGSPGPAGPKG

SPGEAGRPGEAGLPGAKGLTGSPGSPGPDGKTGPAGPAGQDGRPgPPGPPgARGQAGVMG

FPGpkgXaGEPGKAGERGVPGPPGAVGAAGKDGEAGAQGPPGPAGPAGERGEQGPAGSPG

FQGLPGPxGPPGEAGKPGEQGVPGDLGAPGPSGARGERGFPGERGVQGPPGPAGPRGANG

APGNDGAKGDAGAPGAPGSQGAPGLQGMPGERGAAGLPGPKGDRGDAGPKGADGAPGKDG

VRGLTGPIGPPGPAGAPGDKGESGPSGPAGPTGARGAPGDRGEPGPPGPAGFAGPPGADG

QPGAKGEPGDAGAKGDAGPAGPAGPAGPPGPIGNVGAPGPKGARGSAGPPGATGFPGAAG

RVGPPGPSGNAGPPGPPGPVGKEGGKGPRGETGPAGRPGEAGPPGPPGPAGEKGSPGADG

PAGAPGTPGPQGIAGQRGVVGLPGQRGERGFPGLPGPSGEPGKQGPSGTSGERGPPGPMG

PPGLAGPPGESGREGSPGAEGSPGRDGSPGPKGDRGETGPAGxPGAPGAPGAPGPVGPAG

KSGDRGETGPAGPAGPVGPVGARGPTGPQGPRGDKGETGEQGDRGIKGHRGFSGLQGPPG

PPGSPGxQGPSGASGPAGPRGPPGSAGAAGKDGLNGLPGPIGPPGPRGRTGDAGPVGPPG

PPGPPGPPGPPSGGFDFSFLPQPPQEKAHDGGRYYRARQYDGKGVGLGPGPMGLMGPRGP

PGAAGAPGPQGFQGPAGEPGEPGQTGPAGARGPTGPPGKAGEDGHPGKPGRPGERGVVGP

QGARGFPGTPGLPGFKGIRGHNGLDGLKGQPGAPGIKGEPGAPGENGTPGQXGARGLPGE

RGRVGAPGPAGARGSDGSVGPVGPAGPiGSAGPPGFPGAPGPKGElGPVGNPGPAGPAGP

RGEVGLPGLSGPVGPPGNPGANGLTGAKGAAGLPGVAGAPGLPGPRGIPGPPGAVGATGA

RGLVGEPGPAGSKGESGNKGEPGSAGAQGPPGPSGEEGKRGSNGEAGSAGPPGPPGLRGS

PGSRGLPGADGRAGVMGPAGSRGATGPAGVRGPSGDSGRPGEPGLMGPRGFPGSPGNVGP

AGKEGPMGLPGIDGRPGPIGPAGARGEPGNIGFPGPKGPTGDPGKSGEKGHAGLAGPRGA

PGPDGNNGAQGPPGLQGVQGGKGEQGPAGPPGFQGLPGPAGTTGEVGKPGERGLPGEFGL

PGPAGPRGERGPPGESGAVGPSGPIGSRGPSGPPGPDGNKGEPGGVGAPGTAGASGSGGL

PGERGAAGIPGGKGEKGETGLRGEVGSTGRDGARGAPGAIGAPGPAGATGDRGEAGPAGP

AGPAGPRGSPGERGEVGPAGPNGFAGPAGAAGQPGAKGERGTKGPKGENGPVGPTGPAGS

SGPAGPNGPPGPAGSRGDGGPPgXtgfpGAAGRTGPSGPSGITGPPGPPGAAGKEGVRGP

RGDQGPVGRTGETGAGGPPGFTGEKGPSGEPGTAGPPGTPGPQGLLGAPGILGLPGSRGE

RGLPGVAGSVGEPGPLGISGPPGARGPPGAVGNPGVNGAPGEAGRDGNPGNDGPPGRDGQ

PGHKGERGYPGNPGPVGALGAPGPHGPVGPTGKHGNRGEPGPAGSVGPTGAVGPRGPSGP

QGIRGDKGEPGDKGPRGLPGLKGHNGLQGLPGLAGHHGDQGSPGSVGPAGPRGPAGPSGP

AGKDGRTGHPGTVGPAGIRGSQGSQGPAGPPGPPGPPGPPGVSGGGYDFGFDGDFYRA

>Erinaceus

QMSYGYDEKSTGGXSVPGPMGPSGPRGLPGPPGXPGPQGFQGPPGEPGEPGASGPMGPRG

PXGPPGKNGDDGEAGKPGRPGERGPPGPQGARGLPGTAGLPGMKGHRGFSGLDGAKGDSG

PAGPKGEPGSPGENGAPGQMGPRGLPGERGRPGAxGPAGARGNDGATGAAGPPGPTGPXG

PPGFPGAVGAKGEAGPQGXRGSEGPQGXRGEPGPPGPAGAAGPAGNPGADGQPGAKGANG

APGIAGAPGFPGARGPSGPQGPXGPPGPKGNSGEPGAPGNKGDTGAKGEPGPAGVQGPPG

PAGEEGKRGARGEPGPTGLPGPPGERGGPGSRGFPGxDGxAGPKGPAGERGSPGPAGPKG

SPGEAGRPGEAGLPGAKGLTGSPGSPGPDGKTGPPGPAGQDGRPGPPGPPGARGQAGVMG

FPGPKGAAGEPGKAGERGVPGPXGAVGXAGKDGEAGAQGXPGPAGPAGERGEQGPAGSPG

FQGLPGPAGPXGEAGKPGEQGXPGDLGAPGPSGARGERGFPGERGVQGPPGPAGPRGXNG

APGNDGAKGDAGAPGAPGSQGAPGLQGMPGERGAAGLPGPKGDRGDAGPKGADGXPGKDG

XRGLTGPIGPPGPAGAPGDKGESGPSGPAGPTGARGAPGDRGEPGPPGPAGFAGPPGADG

QPGAKGEPGDAGAKGDxGPPGPAGPTGPPGPIGNVGAPGPKGARGAAGPPGATGFPGAAG

RVGPPGPSGNAGPPGPPGPVGKEGGKGPRGETGPAGRPGEAGPPGPPGPAGEKGSPGADG

PAGSPGTPGPQGIAGQRGVVGLPGQRGERGFPGLPGPSGEPGKQGPSGXSGERGPPGPXG

PPGLAGPPGESGREGSPGAEGSPGRDGXPGXKGDRGETGPAGPPGAPGAPGAPGPVGPAG

KXGDRGEXGPAGPAGPXGPXGXRGPXGPQGPRGDKGETGEQGDRGiKGHRGFSGLQGPPG

PPGsPGEQGPSGASGPAGPRGPXGSAGXPGKDGLNGLPGPIGPPGPRGRTGDXGPXGPPG

PPGPPGPPGPPSGGXDxxFLPQPPQEKAHDGGRYYRARQYDGKGVGLGPGPMGLMGPRGP

PGASGAPGPxGFQGPAGEPGEPGQTGPAGARGPTGPPGKAGEDGHPGKPGRPGERGVVGP

QGARGFPGTPGLPGFKGXRGHNGLDGLKGQPGAPGVKGEPGAPGENGTPGQTGARGLPGE

RGRVGAPGPAGARGSDGSVGPVGPAGPIGSAGPPGFPGAPGPKGELGPVGNPGPSGPAGx

RGEVGLPGVSGPVGPPGNPGANGLTGAKGAAGLPGVAGAPGLPGPRGIPGPVGAAGASGA

RGLVGEPGPAGSKGETGNKGEPGSAGAQGxPGPSGEEGKRGQNGEAGSAGPAGPPGLRGS

PGSRGLPGADGRxGVMGPPGSRGASGPAGVRGPSGDSGRPGEPGLMGPRGFPGSPGNVGP

AGKEGPSGLPGIDGRPGPIGPAGARGEPGNIGFPGPKGPSGDPGKSGDKGHAGLAGARGA

PGPDGNNGAQGPPGAQGVQGGKGEQGPAGPPGFQGLPGPAGTTGEXGKPGERGLPGEFGL

PGPAGXRGERGPPGQSGAAGPSGPIGSRGPSGSPGPDGNKGEPGVLGAPGTAGPSGPGGL

PGERGAAGVPGGKGEKGETGLRGEXGXXGRDGARGAPGAXGAPGPAGAXGDRGEAGPAGP

AGPAGPRGSPGERGEVGPAGPNGFAGPAGAAGQPGAKGERGTKGPKGENGIVGPTGPVGA

AGPSGPNGPPGPXGSRGDXGPPGATGFPGAAGRTGPPGPSGITGPPGPPGAAGKEGLRGP

RGDQGPVGRTGETGASGPPGFTGEKGPxGEPGTAGPPGTAGPQGLLGAPGILGLPGSRGE

RGLPGVFGSVGEPGPLGIAGPPGARGPPGAVGNPGVNGAPGEAGRDGNPGSDGPPGRDGQ

PGHKGERGYPGNAGSVGAAGAPGPHGSVGPAGKHGNRGEPGPAGAVGPVGAFGPRGPSGP

QGIRGDKGEPGDKGPRGLPGLKGHNGLQGLPGLAGQHGDQGAPGSVGPAGPRGPAGPSGP

AGKDGRXGQPGTVGPAGIRGSQGNQGPAGPAGPPGPPGPPGPSGGGYDFGYEGDFYRA
